# Extended DNA damage–induced G2/M arrests lead to aberrant mitoses through accumulation of securin

**DOI:** 10.1083/jcb.202511106

**Published:** 2026-11-02

**Authors:** Anton Dubenko, Marah Jnied, Oleksii Kotenko, Svetlana Makovets

**Affiliations:** 1 Institute of Cell Biology, University of Edinburgh, Edinburgh, UK

## Abstract

The stochastic nature of DNA damage dictates various scenarios of stress survival among different cells. We used time-lapse microscopy to perform single-cell level analyses of yeast populations with double-stranded DNA breaks and nonfunctional telomeres. Activation of the DNA damage signalling resulted in a broad distribution in the duration of G2/M arrests. Strikingly, the longer arrests correlated with aberrant mitoses caused by miscoordination of nuclear division and cytokinesis, leading to cell death. Chk1-dependent phosphorylation of Pds1 (securin), an inhibitor of sister chromatid separation in mitosis, was responsible for this phenomenon. Securin progressively accumulated during G2/M arrests, leading to grossly delayed or missed nuclear divisions. This phenotype could be suppressed by slowing down the progression of mitotic exit via *LTE1* deletion or by lowering securin levels. Our results demonstrate that in cells taking too long to complete DNA repair, the DNA damage checkpoint promotes aberrant mitoses and cell death, thereby eliminating cells with a higher chance of genomic instability.

## Teaser

Securin stabilized in response to DNA damage signalling accumulates to excessively high levels during longer G2/M arrests. Upon commitment to mitosis, the completion of securin degradation in these cells is delayed, leading to aberrant nuclear divisions, followed by cell death.

## Introduction

DNA damage signalling is highly conserved in eukaryotes (Lanz et al., 2019). The DNA damage sensor kinases Mec1 and Tel1 in yeast and their homologues ATM and ATR in mammals are recruited to stalled replication forks or DNA breaks, triggering a protein phosphorylation cascade, where Mrc1 and Rad9 mediators facilitate activation of the downstream effector kinases Chk1 and Rad53 (CHK1 and CHK2 in mammals). The effector kinases phosphorylate multiple proteins required to stop cell cycle progression and activate DNA repair.

In yeast, the Pds1 protein, called securin, is one such target for Chk1 kinase (Sanchez et al., 1999; Wang et al., 2001). Securin is an inhibitor of separase Esp1 (Ciosk et al., 1998), which catalyzes proteolytic cleavage of cohesin, a protein physically holding sister chromatids together following DNA replication (Uhlmann et al., 1999). This step is essential for nuclear division and accurate chromosome segregation. The Mec1-Rad9-Chk1–dependent phosphorylation of Pds1 stabilizes securin and delays sister chromatid separation (Cohen-Fix and Koshland, 1997; Sanchez et al., 1999; Wang et al., 2001). This regulatory pathway plays an important role in holding cells with DNA damage in G2/M while DNA repair takes place. At the metaphase-to-anaphase transition, Pds1 is dephosphorylated and degraded by the proteasome (Agarwal et al., 2003; Cohen-Fix and Koshland, 1997). As a result, separase is no longer inhibited, and the sister chromatids can be segregated to daughter cells.

Nonfunctional telomeres resemble DNA breaks (Sandell and Zakian, 1993). If they are too short to recruit enough telomere-binding proteins, the DNA damage sensor kinases recognize them as broken DNA and activate the damage signalling pathway (d’Adda di Fagagna et al., 2003; Enomoto et al., 2002). Telomeres are extended by the enzyme telomerase, which compensates for the telomere shortening during replication (Greider and Blackburn, 1985). Either an insufficiency or a complete loss of telomerase function results in gradual telomere shortening after every replication cycle (Harley et al., 1990; Lundblad and Blackburn, 1990; Lundblad and Szostak, 1989; Yu et al., 1990). This process is called replicative senescence and eventually leads to critically short, nonfunctional telomeres, which activate DNA damage signalling and cell cycle arrest.

In humans, the expression of the catalytic subunit of telomerase, hTERT, is transcriptionally downregulated during embryonic development (Holt et al., 1996; Roake and Artandi, 2020; Sharma et al., 1995). The resultant telomerase insufficiency (TI) is important for inhibiting carcinogenesis by limiting cell proliferation through replicative senescence. However, telomerase becomes reactivated in 85–90% of cancers, and the remaining 10–15% switch to alternative lengthening of telomeres (ALT), a recombination-dependent mechanism for telomere maintenance (Artandi and DePinho, 2010; O’Sullivan and Greenberg, 2025). Similarly, a loss of the telomerase catalytic subunit Est2 in yeast leads to replicative senescence, activation of the DNA damage signalling and G2/M arrest, followed by cell death of the majority of the cell population (Lendvay et al., 1996). However, rare survivors emerge. They are similar to ALT cancer cells in using recombination for telomere maintenance (Lendvay et al., 1996; Lundblad and Blackburn, 1993). Therefore, the telomere dynamics and their interplay with the DNA damage signalling pathway share a lot of similarities in yeast and human cells.

Cell populations experiencing either DNA breaks or telomere maintenance problems are heterogeneous because of the probabilistic nature of DNA damage and telomere dynamics: not every cell in the population experiences the same number of breaks or nonfunctional telomeres in every cell cycle. In addition, the progress of the repair could be different too. The combination of both variabilities would then result in variable, highly individualized cellular response and consequent fate. Often, the dynamics of the DNA damage response are analyzed at a population level, thus producing results that average potentially an extremely wide variety of responses and outcomes at the level of individual cells. Here, we used time-lapse microscopy in combination with fluorescently tagged proteins to investigate budding yeast with TI, telomerase deficiency, and phleomycin-induced DSBs at the single-cell level. In all these settings, we observed a variety of different cell behaviors, from no cell cycle arrest in some cells to a very long time spent in G2 by others. Importantly, the longer cell cycle arrests often resulted in aberrant mitoses leading to cell death, thereby eliminating the cells with hard-to-repair or extensive DNA damage from the populations. Co-visualization of several cellular components combined with genetic analyses allowed us to gain molecular insights into the mechanisms of this pathway, where the amount of Pds1 accumulated during G2/M arrests might be differentiating the cells with shorter and longer arrests.

## Results

### Yeast TI model system

**Figure 1.**
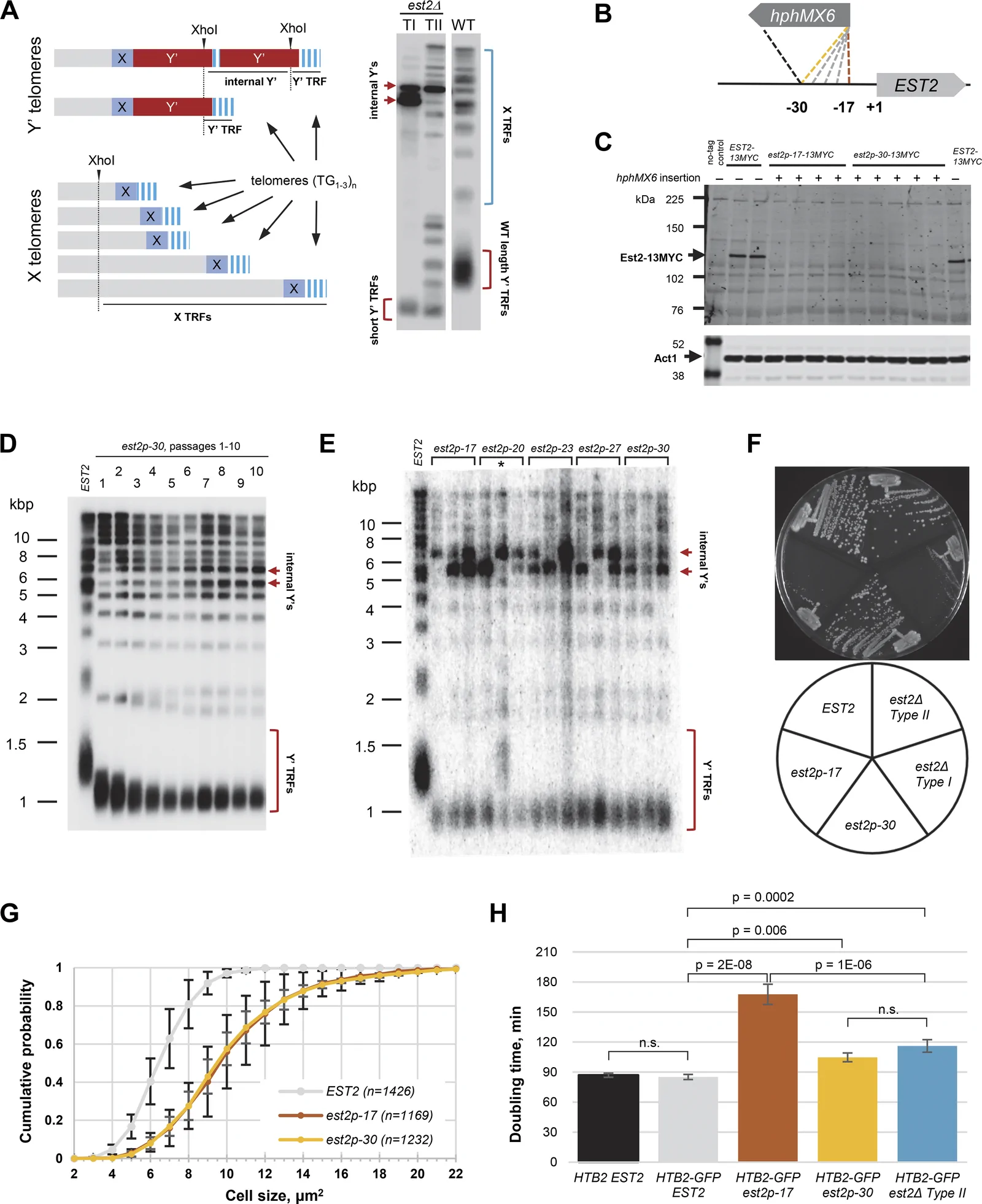
**Construction of yeast strains with TI. (A)** On the left—a schematic of yeast telomeres with the subtelomeric repetitive elements shown. All telomeres contain subtelomeric X-elements (blue) and the actual telomeric (TG_1–3_)_n_ repeats (blue-white stripes). Y’ telomeres also include one or more Y’ repeats (dark red) separated by short stretches of (TG_1–3_)_n_, while the X-telomeres lack Y’s. A restriction digest of genomic DNA with XhoI (dotted lines, cut sites shown by arrows) generates terminal restriction fragments (TRFs), which run on a gel as smears due to the variable length of (TG_1–3_)_n_. The XhoI site in Y’ generates similar TRFs in all the Y’ telomeres. Because XhoI sites are outside of the repetitive elements in X-telomeres, each X telomere generates a unique TRF. On the right—an example of a Southern blot showing telomere pattern changes in *est2Δ* type I (TI) and type II (TII) survivors relative to the WT cells. In WT, TRFs from the X-telomeres run as multiple smeary bands, all above the Y’ TRFs. In type I survivors, all the telomeres gain Y’ repeats (the X TRFs disappear on the blot) and remain short and stable (see short Y’TRFs). Internal Y’s are amplified. In type II survivors, telomeric (TG_1–3_)_n_ repeats are amplified to a different extent at different telomeres, forming a pattern of random-length TRFs on the blot. The internal Y’s are amplified to a lesser extent than in type I survivors. Here and in D and E, an oligonucleotide hybridizing to the telomeric (TG_1–3_)_n_ sequence was used as a probe to visualize telomeres. **(B)** Schematic for mutating the *EST2* promoter via integration of *hphMX6* upstream of the coding region. The position of the first coding nucleotide (A in the ATG) is marked as +1. The positions of the *hphMX6* insertions in the promoter region are indicated relative to the *EST2* coding region. **(C)** Western blot analysis of Est2-13MYC levels in cells with different *hphMX6* promoter insertions. Cells were analyzed immediately after inserting *hphMX6* prior to the manifestation of the growth defect caused by short telomeres. The bottom panel shows an Act1 blot as a control for sample loading. The same protein samples were loaded in the corresponding lanes for both blots, except in the leftmost lane, where the no-MYC-tag control was omitted, and the marker lane is shown instead on the Act1 blot image. **(D)** Southern blot analysis of telomere length through 10 sequential passages after introducing *est2p-30*. Each passage corresponds to ∼ 20 cell doublings. Fragments corresponding to internal Y’ repeats and Y’ TRFs are shown by red arrows and a red bracket, respectively. The rest of the detected bands represent TRFs originating from X telomeres. **(E)** Telomere length and structure analysis of *est2p* mutants by Southern blotting. Three independent clones for each genotype were analyzed after 10 passages (∼200 generations). Parental strain with the native *EST2* promoter is shown in the first lane. One of the clones (marked with *) generated type II survivors. **(F)** Comparative analysis of cell growth on YPD plates. The set of isogenic strains includes the parental *EST2* yeast, the TI mutants *est2p-17* and *est2p-30* with equilibrated telomeres (after 10 passages), as well as telomerase-deficient type I and type II survivors. Cells were streaked from fresh patches onto YPD plates and incubated for 2 days at 30°C before the image was taken. **(G)** Cumulative distribution of the average cell size among cell populations with the normal amount of telomerase and with TI. **(H)** Population doubling time of TI and telomerase-deficient type II survivors in the *HTB2-GFP* strain background. Averages of at least three biological repeats are plotted. The error bars represent standard deviation. Here and in other plots below, p values for different pairwise comparisons are shown above the corresponding square brackets indicating the pairs. p values were calculated using an appropriate post hoc test after a significant ANOVA result for multiple groups or an appropriate variance of the Student’s two-sample two-tailed *t* test for two groups, as described in Materials and Methods. n.s. stands for nonsignificant, i.e., p > 0.05. Source data are available for this figure: SourceData F1.

In a haploid state, *Saccharomyces cerevisiae* carries 16 chromosomes and 32 telomeres. All the telomeres contain ∼350 bps of (TG_1–3_)_n_ telomeric repeats, as well as repetitive X-elements in the subtelomeric regions (Fig. 1 A). About two-thirds of all the telomeres also contain one or more Y’ repeats inserted between (TG_1–3_)_n_ and X-elements (Wellinger and Zakian, 2012). The telomeres with Y’ repeats are called Y’-telomeres, and the rest are X-telomeres (Fig. 1 A). Upon telomerase loss, the (TG_1–3_)_n_ telomeric sequences shorten with every replication cycle, and eventually most cells in the population die due to nonfunctional telomeres. However, a small fraction of cells survives by switching to one of the two recombination-dependent mechanisms of telomere maintenance (reviewed in Wellinger and Zakian [2012]). In type I survivors, all the X-telomeres acquire Y’ repeats and use homologous recombination involving Y’s to maintain short but stable (TG_1–3_)_n_ telomeric tracts, while amplifying Y’ repeats. In type II survivors, recombination operates on the (TG_1–3_)_n_ telomeric tracts, which become very long as recombination amplifies these repeats primarily.

Most work on how yeast deal with nonfunctional telomeres has been done using cells with a complete telomerase loss due to a deletion of a telomerase gene. To engineer a model system to study TI in yeast, we mimicked the effects of the developmentally programmed downregulation of hTERT in humans by weakening the *EST2* promoter. A gene cassette encoding a hygromycin resistance gene (*hphMX6*) was inserted into the promoter in the orientation opposite to that of *EST2* (Fig. 1 B). The terminator end of the cassette was integrated 30 bp away from the *EST2* translation start site (the −30 locus), while the position of the *hphMX6* promoter end varied in different mutants, from −30 to −17, to create a set of *est2p* alleles (for *p*romoter). Analysis of *est2p-17-13MYC* and *est2p-30-13MYC* cells by western blotting showed a decrease in Est2-13MYC protein to undetectable levels (Fig. 1 C). Multiple isolates from independent integration events were passaged 10 times (∼200 generations) on YPD plates, and their telomeres were analyzed by Southern blotting (Fig. 1, D and E). The telomeres shortened during the first few passages and then stayed short and preserved their subtelomeric structure with the exception of gaining more internal Y’ repeats (Fig. 1 D). Although the *est2p* mutants showed a noticeable amplification of Y’ repeats characteristic of telomerase-deficient type I survivors, in most isolates the X-telomeres resembled those of the telomerase-positive parental strain, i.e., retained all the X-telomeres without amplifying the (TG_1–3_)_n_ telomeric repeats, strongly indicating telomerase-dependent telomere maintenance (Fig. 1 E). This was consistent with the lack of a viability crisis normally present during *est2Δ* strain passaging.

Although the *est2p-30* cells grew significantly better on agar plates than the *est2p-17* mutants (Fig. 1 F), both promoter mutations led to an increase in the fraction of large cells in the populations (Fig. 1 G). This is consistent with a frequent activation of the DNA damage checkpoint when the arrested cells experience excessive growth during prolonged G2, as expected from the cells with the telomerase function not sufficient to maintain normal cell cycle progression.

### TI leads to a significant increase in aberrant mitoses, accompanied by loss of nucleolus and cell death

To study TI at the single-cell level using time-lapse microscopy, we introduced the *est2p-17* and *est2p-30* alleles into the strain background with the GFP-tagged histone H2B (Crane et al., 2019; Huh et al., 2003), passaged cells on YPD plates 10–15 times (∼200–300 cell doublings) to establish cultures with equilibrated short telomeres, and confirmed they retained their original telomere structure by Southern blotting (short telomeres, but X-telomeres retained), as described above (Fig. 1 E). As expected, both *est2p-17* and *est2p-30* mutations increased the *HTB2-GFP* population doubling time in YPD broth (Fig. 1 H), with the former causing a more pronounced effect, consistent with the growth defect observed on agar plates (Fig. 1 F). The *HTB2-GFP* allele did not cause any growth defect in the *EST2* background (Fig. 1 H). The *HTB2-GFP EST2* strain is referred to as WT hereafter.

**Figure 2.**
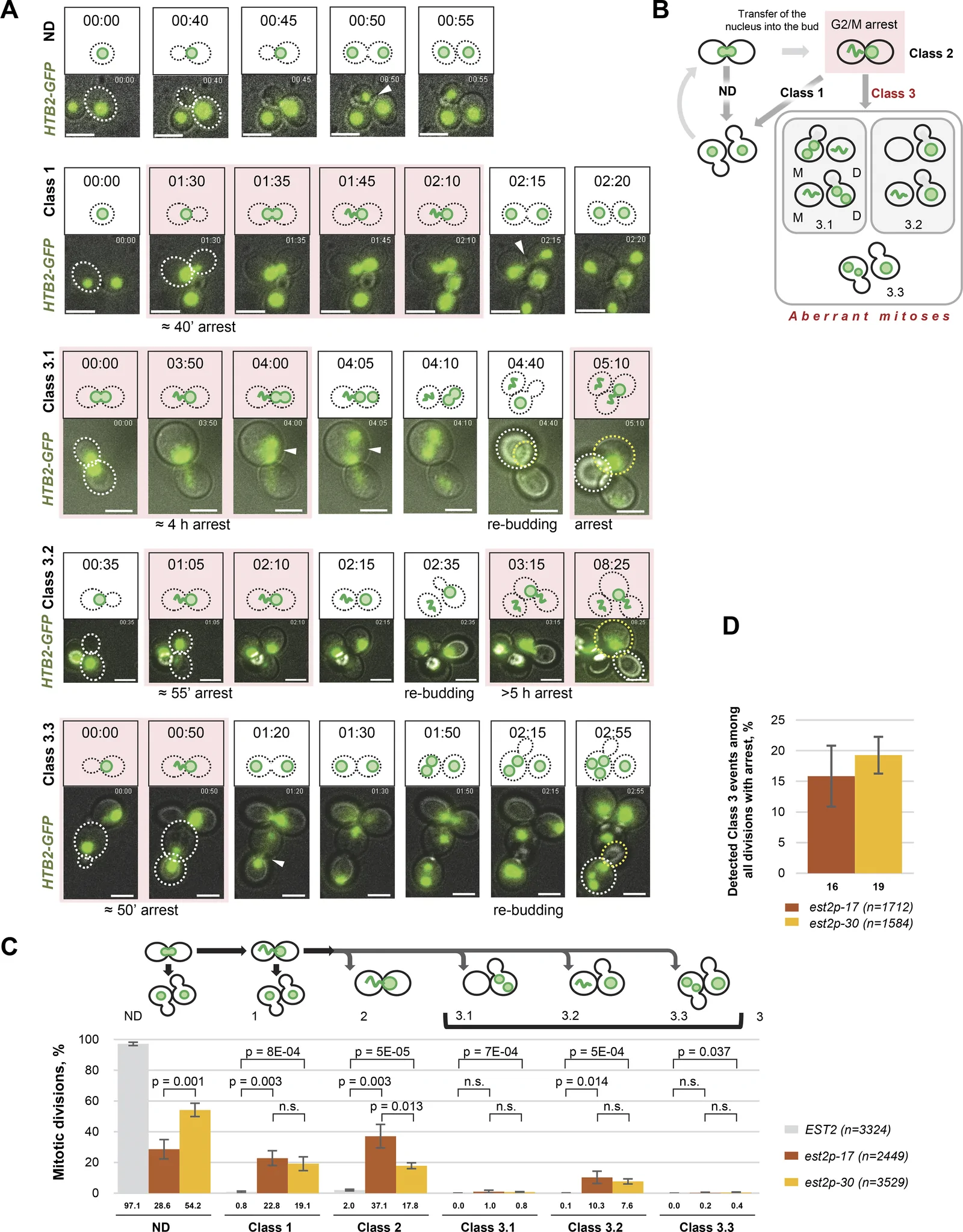
**TI leads to aberrant mitoses. (A and B)** Representative examples of mitotic events (ND, class 1, and class 3.1–3.3, see B) observed using time-lapse microscopy in *est2p-30* cells with Htb2-GFP. Consecutive images of the same field are shown in each set. For each image, a schematic depicting nuclear dynamics (in green) is shown above the image. The time (hours: minutes) shown above each schematic is relative to the start of the imaging. It was used to estimate the length of cell cycle arrests (shown by pink background) and other cell cycle events. White arrows point at nuclear divisions. **(B)** Schematic describing the classification of mitotic divisions in time-lapse microscopy experiments. See text for explanations. **(C)** TI in *est2p-17* and *est2p-30* leads to an increase in aberrant mitoses. Both mutations cause a significant reduction in normal divisions, with *est2p-17* having a stronger effect than *est2p-30*. Most of the aberrant mitoses are of class 3.2, i.e., nuclear division is not detected. Averages (shown below each bar) of at least three biological repeats are plotted. The error bars represent standard deviation. A total number of mitotic events (*n*) scored for each genotype in all the repeats is stated in the legend. **(D)** Probability of aberrant mitoses in *est2p-17* and *est2p-30* cells undergoing G2/M arrest. Percentage of class 3 mitoses among all cells with a G2/M arrest (class 1 + class 2 + class 3) plotted. n values correspond to the sum of class 1 + class 2 + class 3 mitoses in C.

We followed cell divisions over an extended period of time (4–8 h) using time-lapse microscopy, with an image taken every 5 min. Most of the *EST2* cells completed mitoses normally. As expected, they first moved their nuclei to the bud neck and underwent nuclear divisions 5–10 min later (1 or 2 frames). We called these mitotic events Normal Divisions (see ND in Fig. 2, A and B). In contrast, a large fraction of cells with TI experienced cell cycle arrests characterized by extended time spent with the nucleus held at the bud neck. In addition, they showed a distinctly abnormal nuclear morphology, where the majority of the genome was transferred into the bud while a thread-shaped Htb2-GFP signal corresponding to the nucleolus (see below) remained in the mother cell (Fig. 2 B, pink rectangle). There were 3 distinct outcomes following the arrests. Each was categorized into a specific class, 1 through 3. Class 1 included arrested cells which eventually managed to undergo seemingly symmetrical nuclear divisions, and both mother and daughter cells were able to proceed through the subsequent cell cycles (Fig. 2, A and B, class 1). Class 2 cells remained arrested at the time of imaging termination. Thus, these were G2-arrested cells with unclear outcomes of their mitoses, either normal (class 1) or aberrant (class 3), at the end of imaging. Keeping track of these events was important for the overall analysis of the populations, as the class 2 events reflected the overall fraction of cells undergoing cell cycle arrest, as well as the duration of the arrests. Class 3 included cells that went through aberrant mitoses with clearly observed nuclear missegregations (Fig. 2, A and B, class 3). These aberrant mitoses were the main focus of this study.

Depending on the observed nuclear dynamics during aberrant mitoses, we defined 3 subclasses in class 3, from 3.1 to 3.3 (Fig. 2, A and B). Class 3.1 included mitotic events with either a completed or an attempted and reversed nuclear division within a single cell compartment (normally inside the bud, Fig. 2 A, class 3.1, frame 4:05), resulting in one cell retaining the majority of the DNA in the form of a single nucleus or two nuclei, while the other cell inherited very little or no visible DNA. Class 3.2 divisions were characterized by a completely skipped nuclear division, resulting in a cell with the majority of the duplicated genome, predominantly ending up in daughter cells, and an anuclear cell (Fig. 2 A, class 3.2, frames 2:15 and 2:35). In both classes, 3.1 and 3.2, the genome-containing daughters re-budded, arrested in the next cell cycle again (Fig. 2 A: class 3.1 frames 4:40 and 5:10; class 3.2 frames 2:35, 3:15, and 8:25), and in a vast majority of cases died, whereas the mothers remained cadavers with little DNA content. Class 3.3 included any cell divisions with >2 nuclei generated during a single mitosis (Fig. 2 A, class 3.3 frame 2:55).

Analysis of the frequencies of different mitotic events revealed that there were very few cells experiencing detectable arrests and nuclear missegregations in the populations with the normal amount of telomerase, with 97.1% of mitoses classified as ND (Fig. 2 C). In contrast, *est2p-17* and *est2p-30* mutants showed a great reduction in the frequency of normal divisions (28.6% and 54.2% ND, respectively), suggesting that a high fraction of mitoses in the cells with TI involved G2/M arrests prior to cell division. Although most of those resulted in symmetrical nuclear divisions (class 1), a striking increase in aberrant mitoses was also observed (class 3). The majority of these mitoses belonged to the class 3.2 events, where nuclear divisions were not observed (Fig. 2 C). While the frequencies of aberrant mitoses were at 11.5% and 8.8% of all the scored mitotic events in *est2p-17* and *est2p-30* populations respectively, the probabilities of class 3 events following a G2/M arrest (calculated as $\frac{Class\;3}{Class\;1+Class\;2+Class\;3}$ ) were significantly higher (Fig. 2 D). It should be pointed out that these values are underestimated, as some of the class 2 cells would have resolved in aberrant mitoses. Because *est2p-30* had an easily detectable aberrant mitosis phenotype while showing a milder growth defect in comparison with *est2p-17*, we used *est2p-30* for further experiments.

**Figure 3.**
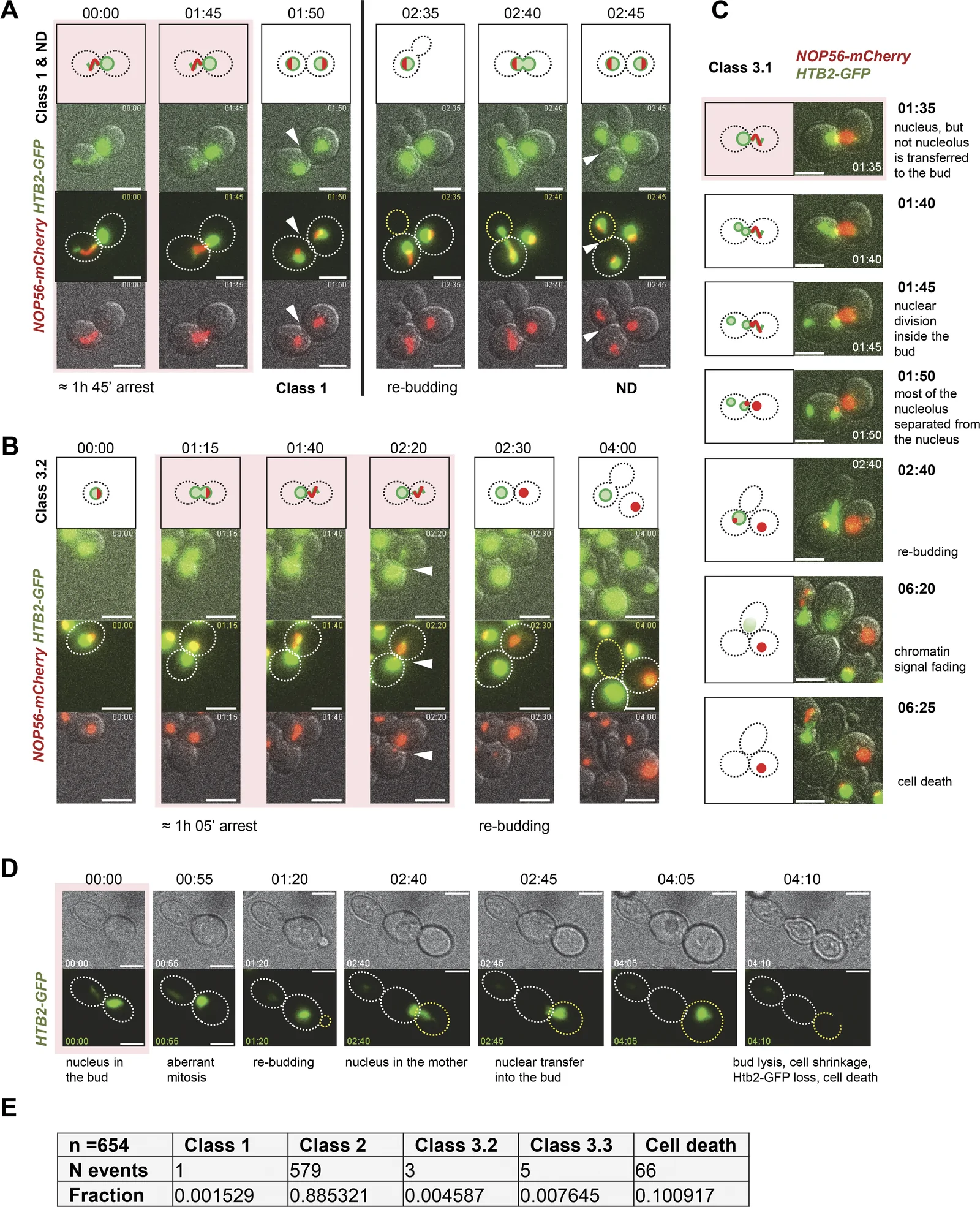
**TI leads to a significant increase in aberrant mitoses, accompanied by loss of nucleolus and cell death. (A–C)** Representative examples of mitotic events in cells with TI: class 1 and ND in A; class 3.2 in B and class 3.1 in C, with co-visualized nucleus (Htb2-GFP) and nucleolus (Nop56-mCherry). Consecutive images of the same field during time-lapse microscopy are shown in each set. For each image, a schematic depicting nuclear (in green) and nucleolar (in red) dynamics is shown above (A and B) or next to the image (C). The time (hours: minutes) shown above each schematic (A and B) or on the left of each image (C) is relative to the start of the imaging. This time was used to estimate the length of cell cycle arrests (shown by pink background) and other events. Notice that in A, class 1 and ND events come from imaging two consecutive divisions of the same cell. Arrows point at nuclear divisions. Here and in the figures below, white cell outlines are used for the initial cell divisions and yellow outlines for the later emerging buds (re-budding) and the cells derived from them. **(D)** A representative example of cell death with clearly observed bud lysis following an aberrant mitosis. Labelling around images as in A–C. **(E)** Analysis of cell fate following aberrant mitoses. 654 *est2p-30* cells, which underwent aberrant mitoses, were analyzed in the following cell cycle, and the observed results were summarized in the table.

Following class 3.1 and 3.2 aberrant mitoses, cells often died, most likely as a result of gross missegregation of the nucleolus from the rest of the nucleus. We found that, indeed, the thread-shaped Htb2-GFP signal often remained in the anuclear cell co-localized with the nucleolar protein Nop56 (Fig. 3, A–C). Despite inheriting most of the genome, the sibling cell eventually died (see Fig. 3, C and D for representative examples). We followed 654 *est2p-30* cells after either class 3.1 or 3.2 mitosis. All of the nucleus-inherited cells budded and underwent another G2/M arrest in the following cell cycle (Fig. 3 E). Around 90% of them remained arrested by the end of the imaging (class 2). Of the remaining 75 cells, only 1 underwent a seemingly symmetrical nuclear division, 8 experienced another aberrant mitosis, and 66 cells died. Therefore, TI results in a vastly increased probability of aberrant mitoses and cell death.

### Aberrant mitoses result from DNA damage-induced G2/M arrests

**Figure S1.**
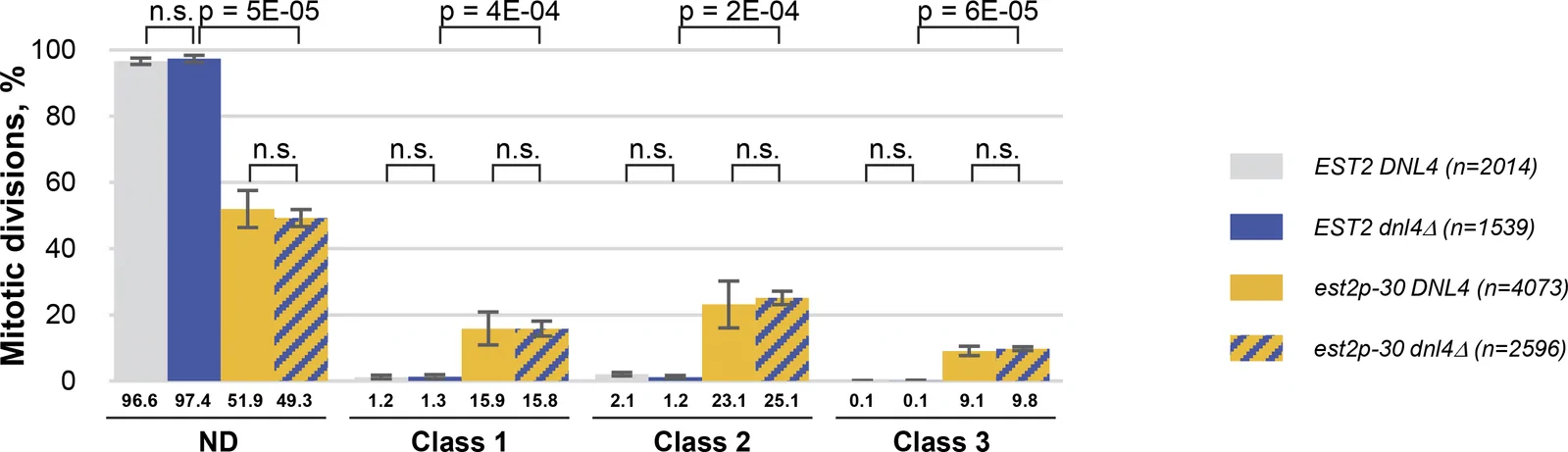
**The effect of *dnl4Δ* on genome segregation in cells with TI.** Averages (shown below each bar) of at least three biological repeats are plotted. The error bars represent the standard deviation. A total number of mitotic events (*n*) scored for each genotype in all the repeats is stated in the legend.

Short telomeres are known to fuse through nonhomologous end joining (NHEJ), which might then impair chromosome segregation (Mieczkowski et al., 2003). To test this hypothesis, we inactivated NHEJ by deleting *DNL4*, the gene coding for the DNA ligase responsible for telomere fusions. However, the loss of *DNL4* had no effect on chromosome segregation in cells with TI (Fig. S1). Therefore, the increase in aberrant mitoses in response to TI was not due to telomere–telomere fusions.

**Figure 4.**
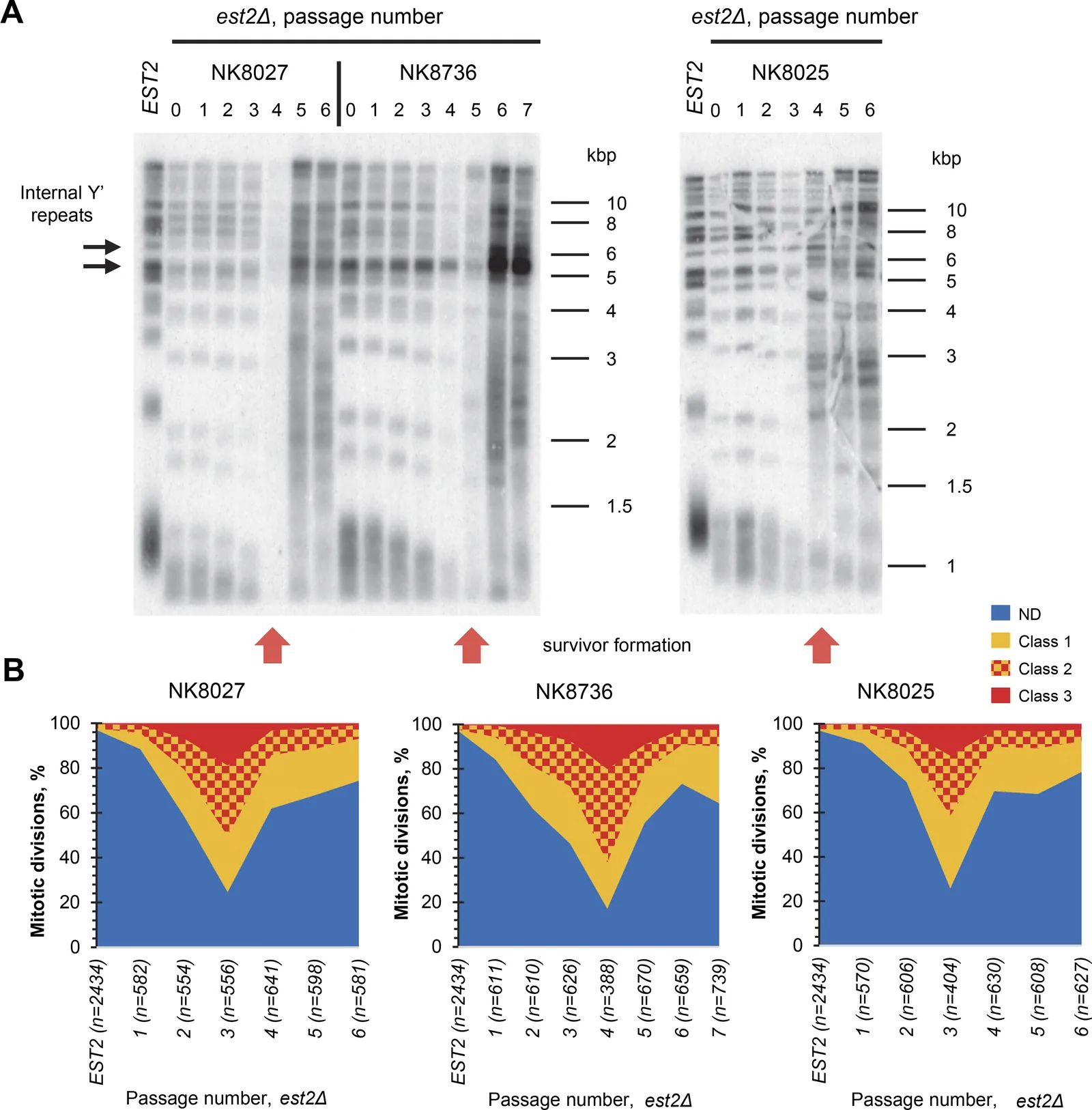
**Comparative analysis of aberrant mitoses during replicative senescence of three independent *est2Δ* isolates. (A)** Southern blot analysis of telomeric DNA isolated from the cell samples taken during serial passaging of *est2Δ* mutants upon *EST2* deletion. The parental strain sample is in the first lane of each blot (*EST2*). Passage 0 corresponds to the *est2Δ* colony after the gene deletion transformation. Red arrows indicate a survivor formation stage. All three *est2Δ* isolates formed type II survivors, as evident from long telomeres forming a pattern of random-size smears through each lane. Each passage corresponds to ∼ 20 cell divisions. **(B)** Mitotic events analyzed in the samples of the senescing cells used for the telomere analysis in A. Source data are available for this figure: SourceData F4.

We next asked if the residual telomerase in the *est2p-30* mutants played any role in the aberrant mitoses or if this phenotype was due to their short telomeres. *EST2* was deleted in *HTB2-GFP* cells, and the telomerase-deficient populations were assayed through replicative senescence. Unlike the cells with TI, telomerase-deficient cells cannot maintain their telomeres short and stable. Instead, they undergo a cell viability crisis followed by a switch to recombination-dependent telomere maintenance (Lundblad and Blackburn, 1993). As the telomeres shortened (Fig. 4 A), the fraction of normal divisions decreased, reaching its lowest percentage at the point of the viability crisis (Fig. 4 B). At the same time, the fractions of the cells undergoing G2/M arrests increased, and the aberrant mitoses paralleled this trajectory, reaching the maximum frequency at the point of the cell viability crisis when the telomeres were the shortest. As the populations generated type II survivors, the normal divisions became more frequent again, and the aberrant mitoses decreased. Thus, the aberrant mitoses were not specific to the cells with TI but correlated with the presence of short telomeres.

**Figure 5.**
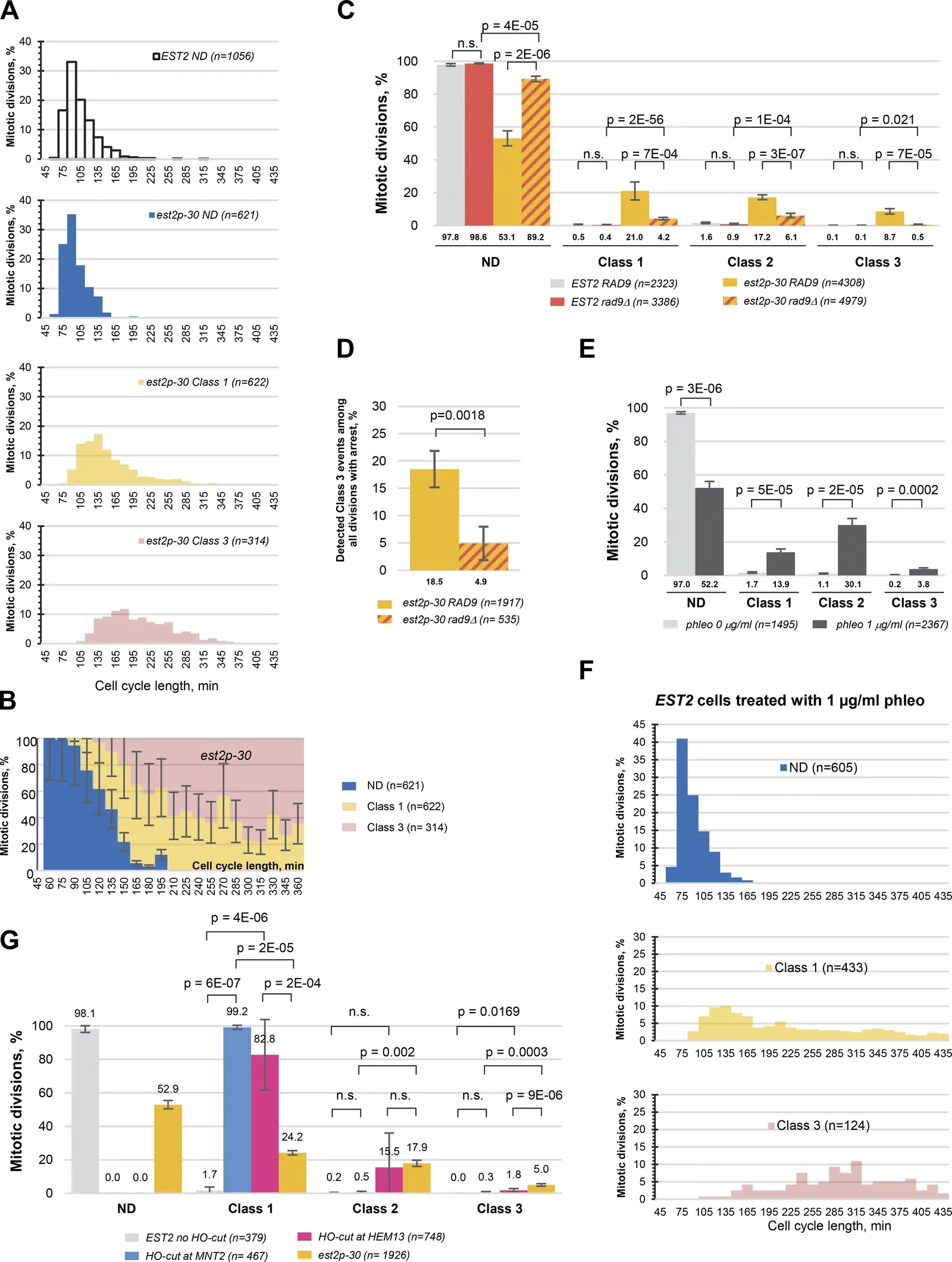
**Aberrant mitoses result from DNA damage–induced G2/M arrests. (A)** Distribution of completed mitoses in *est2p-30* cells according to the cell cycle duration. **(B)** The probability of aberrant mitoses in *est2p-30* cells increases with the cell cycle length. For each bin, including mitotic events with the same duration of the cell cycle (see A), fractions of ND, class 1, and class 3 events of the total mitoses with the same duration of the cell cycle (shown on the X-axis) were calculated independently for each of the three biological repeats. Average values are plotted, and the error bars represent standard deviation. **(C)***RAD9* deletion suppresses the high frequency of aberrant mitoses in response to TI. **(D)** Probability of aberrant mitoses in *est2p-30* cells undergoing G2/M arrest depends on *RAD9*. Percentage of class 3 mitoses among all cells with a G2/M arrest (class 1 + class 2 + class 3) plotted. *n* values correspond to the sum of class 1 + class 2 + class 3 mitoses in C. **(E)** Addition of phleomycin to the growth medium results in an increase in aberrant mitoses. **(F)** Distribution of completed mitoses in WT cells grown in the presence of phleomycin according to the cell cycle duration; see E. **(G)** A single unrepairable DSB increases the probability of aberrant mitoses. Only cells with detected Rad52-mNeonGreen foci in response to HO-induced DSBs were included in the analyses. *est2p-30* data are included in the chart for a side-by-side comparison. **(A, B, and F)** Blue, yellow, and pink colors represent ND, class 1, and class 3 mitoses, respectively. **(C–E, and G)** Averages (shown either below or above each bar) of at least three biological repeats are plotted for each genotype. The error bars represent standard deviation. A total number of mitotic events (*n*) scored for each genotype in all the repeats is stated in the legend.

Telomerase-deficient cells undergo longer cell cycle arrests during the later stages of senescence (Xu et al., 2015). Therefore, the increase in aberrant mitoses during senescence observed in Fig. 4 might be due to the increase in the duration of G2/M arrests. To test this hypothesis, we reanalyzed the microscopy data for *est2p-30* mutants (Fig. 2 C) by binning the mitotic events according to the duration of the cell cycle, which was calculated as the time between nuclear segregations in consecutive cell cycles in ND and class 1 events or between the preceding nuclear segregation and cutting off the thread-shaped nucleolar Htb2-GFP signal in class 3 events (Fig. 5, A and B). Indeed, the aberrant mitoses correlated with the longer cell cycles, pointing toward the duration of the G2/M arrests being an important factor in determining the outcome of cell divisions.

Lack of the key component of the DNA damage checkpoint Rad9 in telomerase-negative cells leads to a dramatic reduction in the fraction of large G2 arrested cells (IJpma and Greider, 2003; Enomoto et al., 2002; Sandell and Zakian, 1993). Loss of *RAD9* in *est2p-30* cells resulted in a suppression of aberrant mitoses (Fig. 5 C). This suppression could not be attributed solely to the decrease in the fraction of cells undergoing G2/M arrests, because when the frequency of aberrant mitoses was calculated only among the cells undergoing G2/M arrests (>10 min G2), a significant decrease in aberrant mitoses remained (Fig. 5 D). Therefore, the DNA damage checkpoint activation increases the probability of an aberrant mitosis.

If the DNA damage-induced G2/M arrests contribute to aberrant mitoses, then a similar phenotype might be observed in response not only to short telomeres but also to other signals triggering the DNA damage signalling pathway. *HTB2-GFP EST2* cells were grown in the presence of phleomycin, a drug causing DNA breaks, and their mitotic behavior was analyzed using time-lapse microscopy (Fig. 5 E). In the presence of phleomycin, more cells underwent G2 arrest (lower NDs). More importantly, the fraction of aberrant mitoses also increased in response to phleomycin (Fig. 5 E, class 3), and the aberrant mitoses correlated with longer cell cycles (Fig. 5 F).

We next used a single unrepairable DSB introduced by galactose-inducible expression of HO-endonuclease as a cause of DNA damage. The HO recognition sequence was positioned either next to *HEM13* located in the middle part of chromosome IV (Makovets and Blackburn, 2009) or at a subtelomere on chromosome VII (*MNT2-HOsite-URA3-telomere*). These cells also contained Rad52-mNeonGreen, allowing analysis of only the cells with induced and resected breaks as judged by the appearance of Rad52 foci (Fig. 5 G). Cells with subtelomeric breaks divided multiple times, with the Rad52-mNeonGreen focus re-establishing after each division. This behavior could be explained by weaker checkpoint activation from DNA resection generating significant stretches of ssDNA only on one side of the break: the DNA fragment on the other side of the HO cut is very short and degraded completely. A single aberrant mitosis out of the 467 cells analyzed was detected during the first cell divisions following a subtelomeric break induction. In contrast, the cells with the breaks in the middle of chromosome IV could complete only the first division, with 1.8% of them undergoing aberrant mitoses. All these cells arrested again in the following cell cycle and died, presumably due to the loss of essential DNA as a result of excessive resection. Therefore, not only short telomeres but also DNA damage in non-telomeric regions of the genome can cause genome missegregations. Taken together, all the experiments presented in Figs. 4 and 5 suggest that prolonged G2/M arrests in response to DNA damage signalling triggered by either short telomeres or DSBs result in increased frequencies of aberrant mitoses.

### Accumulation of Pds1 during prolonged G2/M arrests leads to aberrant mitoses

**Figure 6.**
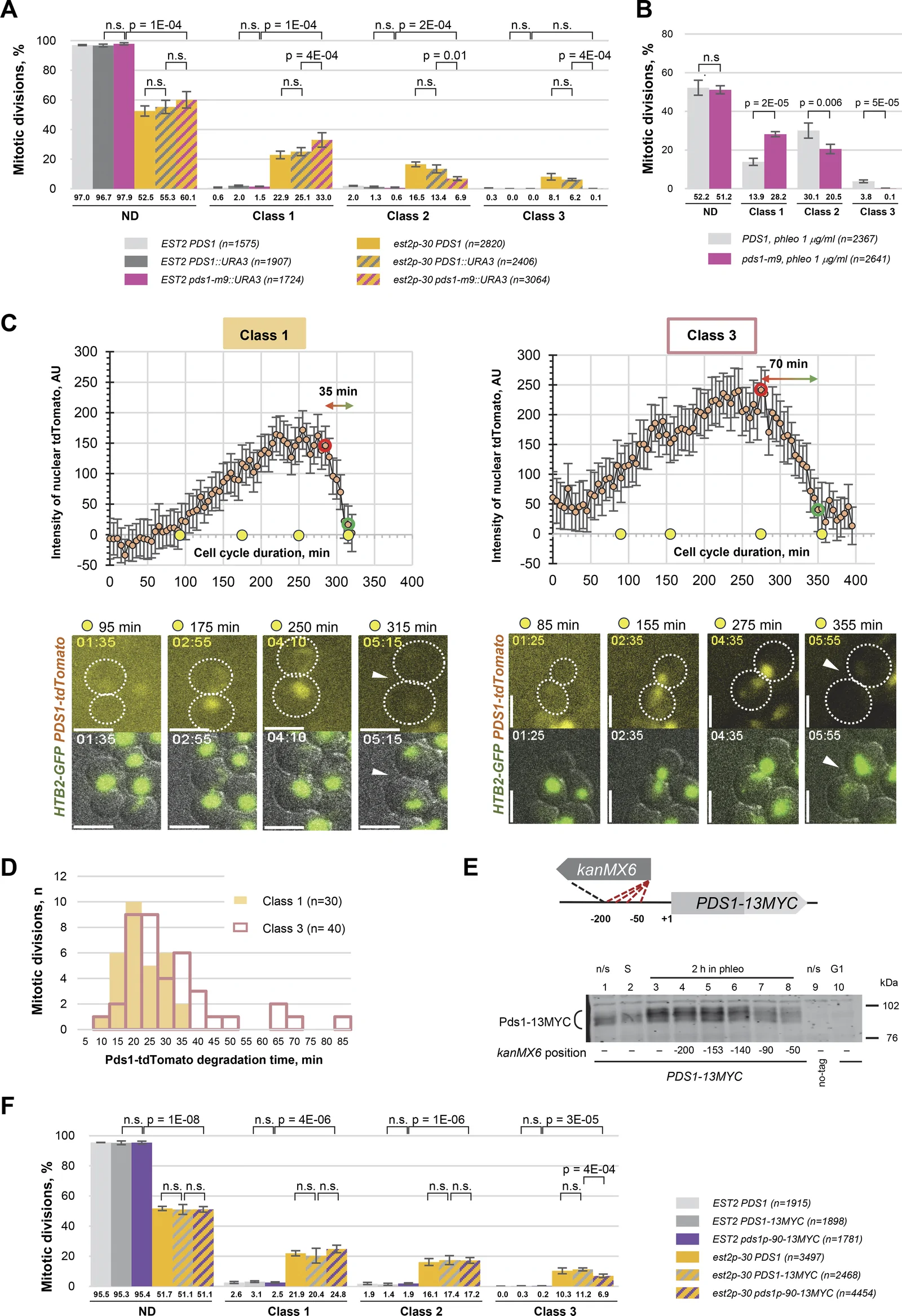
**Accumulation of Pds1 during prolonged arrests leads to aberrant mitoses. (A)** *pds1-m9* suppresses aberrant mitoses in *est2p-30* cells. **(B)***pds1-m9* suppresses aberrant mitoses caused by cell growth in the presence of phleomycin. **(C)** Representative examples of analyses of Pds1-tdTomato dynamics observed using time-lapse microscopy in class 1 and class 3 mitoses. The intensity of the nuclear tdTomato signal over background during the observed cell cycle is plotted. Cell images for four time points indicated by yellow circles on the X-axis are shown below the plot. White arrows point to nuclear divisions. Error bars show the standard deviation of pixel intensity. Here and in D, time 0 is defined as the maximum separation of the nuclei following the cell division in the previous cell cycle. 43 class 1 events and 42 class 3 events (class 3.1 and class 3.2) in *est2p-30* cells were analyzed. The majority of them showed similar Pds1-tdTomato dynamics of an upside-down U shape, where the levels of tdTomato signal were significantly higher than the background during the arrest: 30/43 of class 1 events, 40/42 of class 3 events. Pds1 degradation time (red-green double-headed arrow) was determined as the period between the latest time point at the plateau on the top of the curve (red circle) and the earliest time point reaching the same Pds1 levels as before its accumulation began (green circle). **(D)** Pds1 degradation time prior to class 1 and class 3 mitoses in *est2p-30* cells. Individual cell data from the analyses described in C (*n* = 30 for class 1; *n* = 40 for class 3) were binned according to the duration of Pds1 degradation estimated as shown in C. According to the Mann–Whitney U test, the distributions of the two groups are statistically significantly different (p = 0.0043). **(E)** Construction of *PDS1* alleles with decreased gene expression. Top—schematic for mutating the *PDS1* promoter via *kanMX6* integration upstream of the protein-coding region. The position of the first coding nucleotide is marked as +1. The positions of the *kanMX6* insertions in the promoter region are indicated relative to the coding region. The terminator end of *kanMX6* in all the constructs is anchored 200 bp upstream of the *PDS1* coding region, while the positions of the *kanMX6* promoter end varied in five different constructs: −200, −153, −140, −90 and −50. Bottom—western blot analysis of Pds1-13MYC levels during G2/M arrests. Cells were grown in YPD, synchronized in G1 by α-factor for 2 h, and then released into YPD with 2 µg/ml phleomycin. Aliquots for the *PDS1-13MYC* strain were taken right before (G1, lane 10) and immediately after (S, lane 2) the G1 release, as well as after 2 h of incubation (lane 3). The *PDS1* promoter mutants are shown only after 2-h incubation in phleomycin, following the G1 release (lanes 4–8). Lanes showing control samples of nonsynchronous *PDS1-13MYC* (lane 1) and *PDS1* (no tag control, lane 9) cells are labelled n/s. Protein size marker positions are shown on the left. **(F)***pds1p-90* suppresses aberrant mitoses in *est2p-30* cells. In A, B, and F, averages of at least three biological repeats are plotted. The error bars represent the standard deviation. A total number of mitotic events (*n*) scored for each genotype in all the repeats is stated in the legend. Source data are available for this figure: SourceData F6.

What is the molecular mechanism linking the length of the G2/M arrest with the frequency of missegregation events? Strikingly, most of the aberrant mitoses are represented by the majority of the chromosomes being pulled all together into the same cell (class 3.2), rather than random genome missegregation. This suggests the possibility that the sister chromatids remain connected. Pds1 (securin) is one of the downstream targets of the DNA damage signalling. It is phosphorylated by the DNA damage checkpoint kinase Chk1 to stabilize Pds1 in order to delay cohesin cleavage by separase, thereby prolonging G2/M arrests in response to DNA damage (Wang et al., 2001). We hypothesized that the stabilization of Pds1 might lead to its excessive accumulation with the increasing length of the G2/M arrest and interfere with the timely activation of separase. To test if Pds1 phosphorylation played a role in the increase in aberrant mitoses in cells with TI, a previously characterized phosphorylation-deficient allele *pds1-m9* (nine serine/threonines are replaced with alanines as described by Wang et al. [2001]) was combined with *est2p-30* and the double mutants along with the appropriate isogenic control strains were analyzed by time-lapse microscopy, as described above. The *pds1-m9* mutation did not affect the probability of a G2/M arrest in response to TI (ND: 55.3% in *PDS1* vs. 60.1% in *pds1-m9*), but it slightly decreased the duration of the arrests (lower Class 2) and, more importantly, drastically reduced the probability of an aberrant mitosis, from 6.2% in *PDS1* control to 0.1% in *pds1-m9* (Fig. 6 A). The reduction in the class 3 events was accompanied by an increase in the class 1 events, i.e., more postarrest cells with the *pds1-m9* allele segregated their genomes with symmetrical nuclear divisions instead of going through aberrant mitoses. Similar results were obtained using phleomycin as a DNA damage signalling trigger instead of TI (Fig. 6 B). Therefore, the phosphorylation of Pds1 in response to DNA damage plays an important role in aberrant mitoses in cells with G2/M arrests.

Next, we followed the levels of Pds1-tdTomato at the single-cell level in *est2p-30* mutants with prolonged G2/M arrests (Fig. 6, C and D). The Pds1-tdTomato signal was below the detectable levels at the earlier stages of the arrests, but the protein gradually accumulated and became easily detectable before disappearing again (Fig. 6 C). Therefore, Pds1 progressively accumulates over the duration of G2/M arrests, and the more securin is accumulated, the longer it may take to degrade it. We compared class 1 and class 3 mitoses by the time it took them to bring the accumulated Pds1 to the levels before the accumulation began (Fig. 6 C) and binned all the analyzed individual events to make the comparison (Fig. 6 D). Although the two classes had a significant overlap at the lower Pds1 degradation intervals, the statistical analysis revealed that aberrant mitoses (class 3) correlated with a longer time required to degrade Pds1.

Our hypothesis proposes that excessive accumulation of Pds1 during prolonged arrests might be responsible for aberrant mitoses by interfering with separase activation. Consequently, we reasoned that lowering the excessive Pds1 levels might suppress aberrant mitoses caused by TI. To decrease *PDS1* expression in general, we used the same strategy as the one described above for *EST2* (Fig. 1, B and C). *kanMX6* cassette coding for a kanamycin resistance gene was inserted at different positions in the *PDS1* promoter in a strain with a *PDS1-13MYC* allele (Fig. 6 E, top). Cells carrying a *kanMX6* insertion in different positions were grown in the presence of phleomycin to induce G2/M arrests, and the levels of Pds1-13MYC were analyzed using anti-MYC antibodies (Fig. 6 E, bottom). *pds1p-90* and *pds1p-50* resulted in noticeable reductions in the Pds1-13MYC levels. *pds1p-90-13MYC* was combined with *est2p-30,* and the mitotic behavior of the double mutant strains, as well as of the isogenic control yeast, was analyzed using time-lapse microscopy (Fig. 6 F). Reducing the levels of *PDS1* expression did not decrease the detectable fraction of the cells experiencing G2/M arrests, but it led to a partial suppression of aberrant mitoses. Thus, excessive Pds1 in the cells with DNA damage–induced G2/M arrests is responsible for more frequent aberrant mitoses.

### Aberrant mitoses stem from a loss of coordination between nuclear divisions and cytokinesis

**Figure 7.**
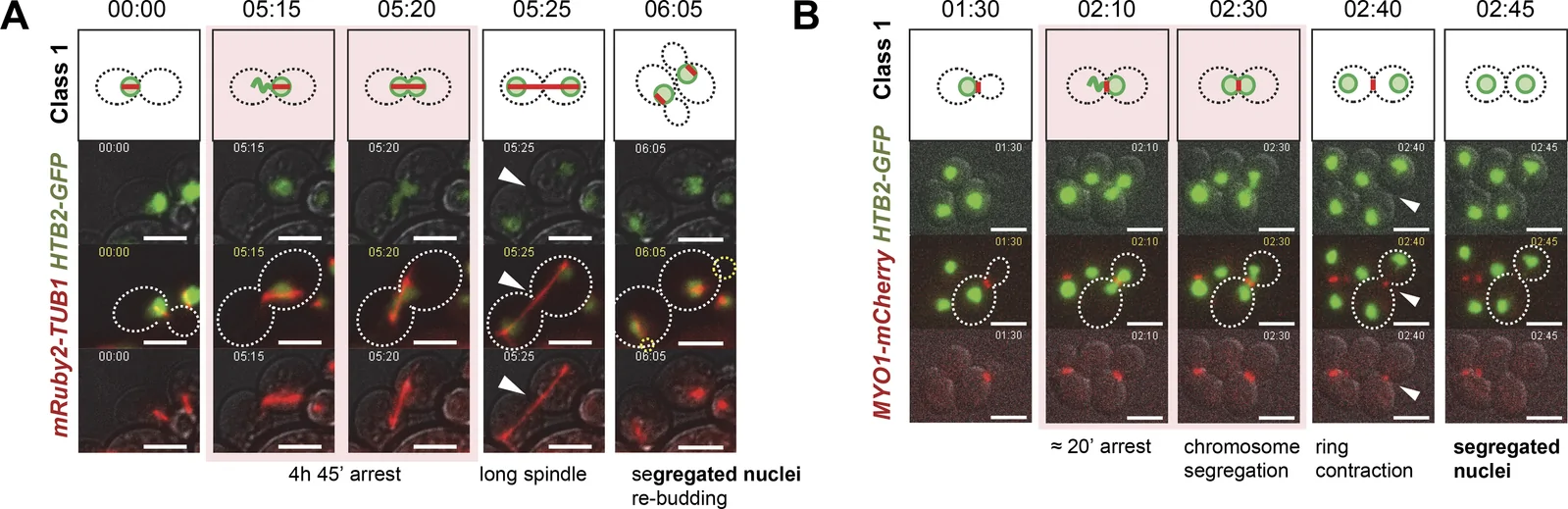
**Coordination between** **nuclear division** **and cytokinesis during class 1 mitoses. (A and B)** Representative examples of mitotic events observed using time-lapse microscopy. Consecutive images of the same field are shown in each set, as explained in the legend to Fig. 2. White arrows point to nuclear divisions. **(A)** Co-visualization of mitotic spindle (mRuby2-Tub1) and chromatin (Htb2-GFP) during a class 1 mitosis. **(B)** Co-visualization of actomyosin ring (Myo1-mCherry) and chromatin (Htb2-GFP) during a class 1 mitosis.

**Figure 8.**
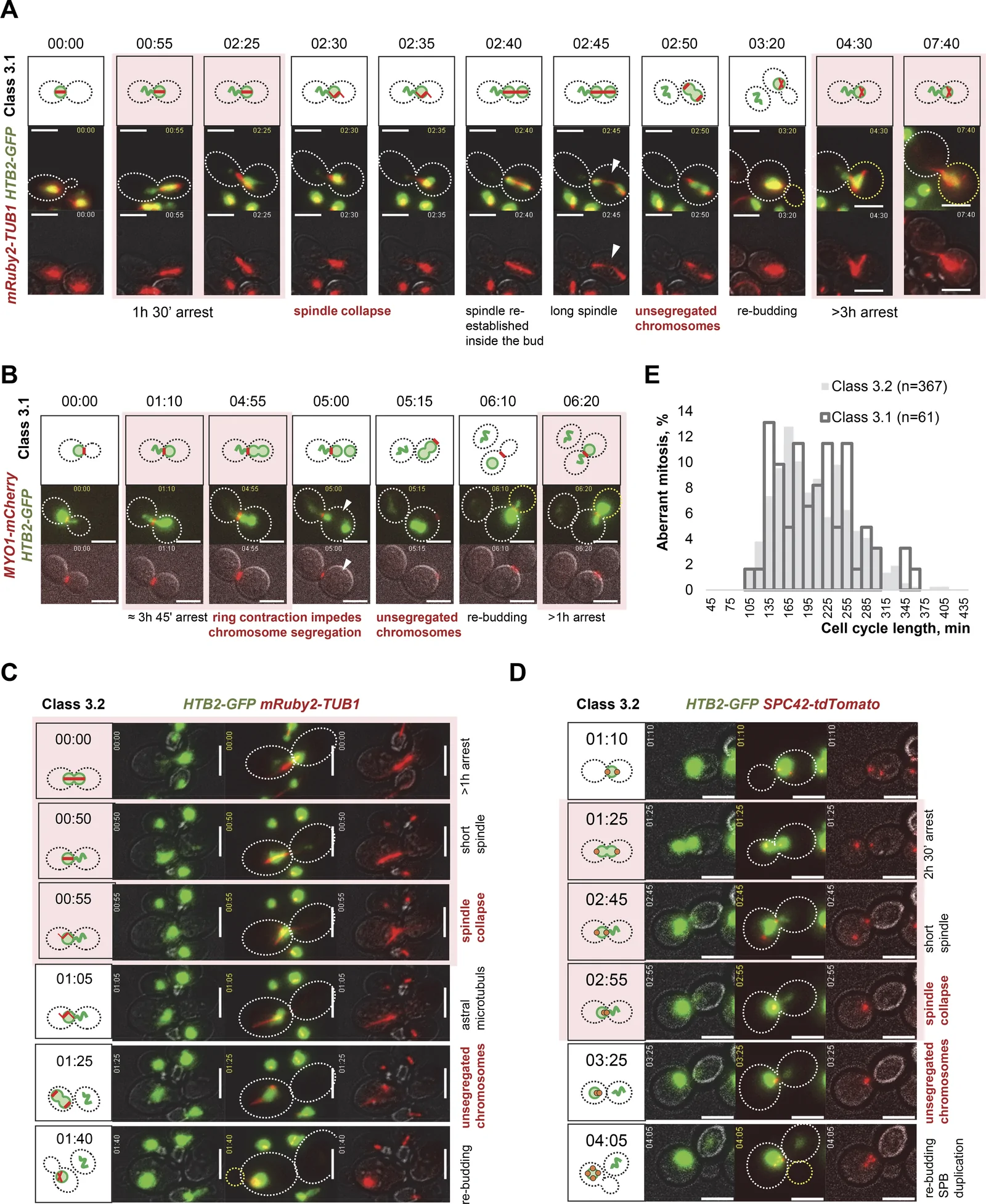
**TI leads to a loss of coordination between** **nuclear division** **and cytokinesis. (A–D)** Representative examples of mitotic events observed using time-lapse microscopy in class 3 mitoses. Consecutive images of the same field are shown in each set, as explained in the legend to Fig. 2. White arrows point to nuclear divisions. **(A)** Co-visualization of mitotic spindle (mRuby2-Tub1) and chromatin (Htb2-GFP) during a class 3.1 aberrant mitosis. **(B)** Co-visualization of actomyosin ring (Myo1-mCherry) and chromatin (Htb2-GFP) during a class 3.1 aberrant mitosis. 148 class 3 events in *est2p-30* cells were examined (128 class 3.2, 20 class 3.1), and all showed similar cytokinesis dynamics to the presented example. **(C)** Co-visualization of mitotic spindle (mRuby2-Tub1) and chromatin (Htb2-GFP) during a class 3.2 aberrant mitosis. 73 class 3 events in *est2p-30* cells were examined (50 class 3.2, 23 class 3.1), and all showed similar spindle dynamics to the presented example. **(D)** Co-visualization of spindle pole bodies (Spc42-tdTomato) and chromatin (Htb2-GFP) during a class 3.2 aberrant mitosis. 81 class 3 events in *est2p-30* cells were examined (71 class 3.2, 10 class 3.1), and all showed similar spindle pole body dynamics to the presented example. **(E)** Cell cycle length does not determine a particular outcome of aberrant mitosis. Distributions of completed aberrant mitoses in *est2p-30* cells according to the cell cycle duration. Class 3.2 and class 3.1 events are compared. These distributions are not normal. According to the Mann–Whitney U test, there is no statistically significant difference between the two populations.

To gain mechanistic insights into the aberrant mitoses, we co-visualized Htb2-GFP with one of the components of the mitotic machinery, such as spindle microtubules (mRuby2-Tub1), spindle pole bodies (SPB, Spc42-tdTomato), and the actomyosin ring (Myo1-mCherry) involved in cytokinesis, and compared the dynamics of cellular components in *est2p-30* cells during class 1 and class 3 mitoses (Figs. 7 and 8). In both cases, the cells were dividing after a G2/M arrest but underwent either seemingly normal or aberrant mitoses. In class 1 cells, shorter spindles extended into long spindles characteristic of normal mitoses (Fig. 7 A), and the actomyosin ring contracted following chromosome segregation into mother and daughter cells (Fig. 7 B). In contrast, during class 3.1 mitoses short spindles collapsed (became short) first and then re-established and elongated inside the buds/daughter cells during attempted nuclear divisions, which occurred within a single cell (Fig. 8 A, frames 2:40 and 2:45). These nuclear divisions either reversed before completion and resulted in a single undivided nucleus or went all the way to the end, yielding binuclear cells.

Why did the nuclear divisions happen inside the bud? We hypothesized that premature cytokinesis, which occurs in yeast via actomyosin ring contraction at the bud neck, might have created a physical barrier by separating the mother and daughter cells before the nuclear division took place. Indeed, in class 3.1 divisions, the ring contraction began before the genetic material segregated into daughter cells (Fig. 8 B, frame 5:00) and could be seen as an obstacle to genome segregation. Therefore, the premature ring contraction might be responsible for physically separating the nucleolus from the rest of the genome during aberrant mitoses (Fig. 3, B and C).

During class 3.2 mitotic events, short spindles collapsed, as evident by (a) mRuby2-Tub1 signal converting into a single spot with radiating astral microtubules (Fig. 8 C) and (b) merging SPBs (Fig. 8 D). As nuclear division did not take place, the SPB reduplication resulted in four bright Spc42-tdTomato spots within a single cell (Fig. 8 D). Taken together, these observations suggest that the aberrant mitoses might come from either delayed or completely missed nuclear divisions, where cytokinesis might happen before the nucleus could divide.

We asked if the class 3.1 and class 3.2 mitoses were different in the duration of the preceding cell cycle arrests. However, no such difference was observed (Fig. 8 E). Therefore, some other molecular factors might be contributing to the particular outcomes of the aberrant mitoses.

**Figure 9.**
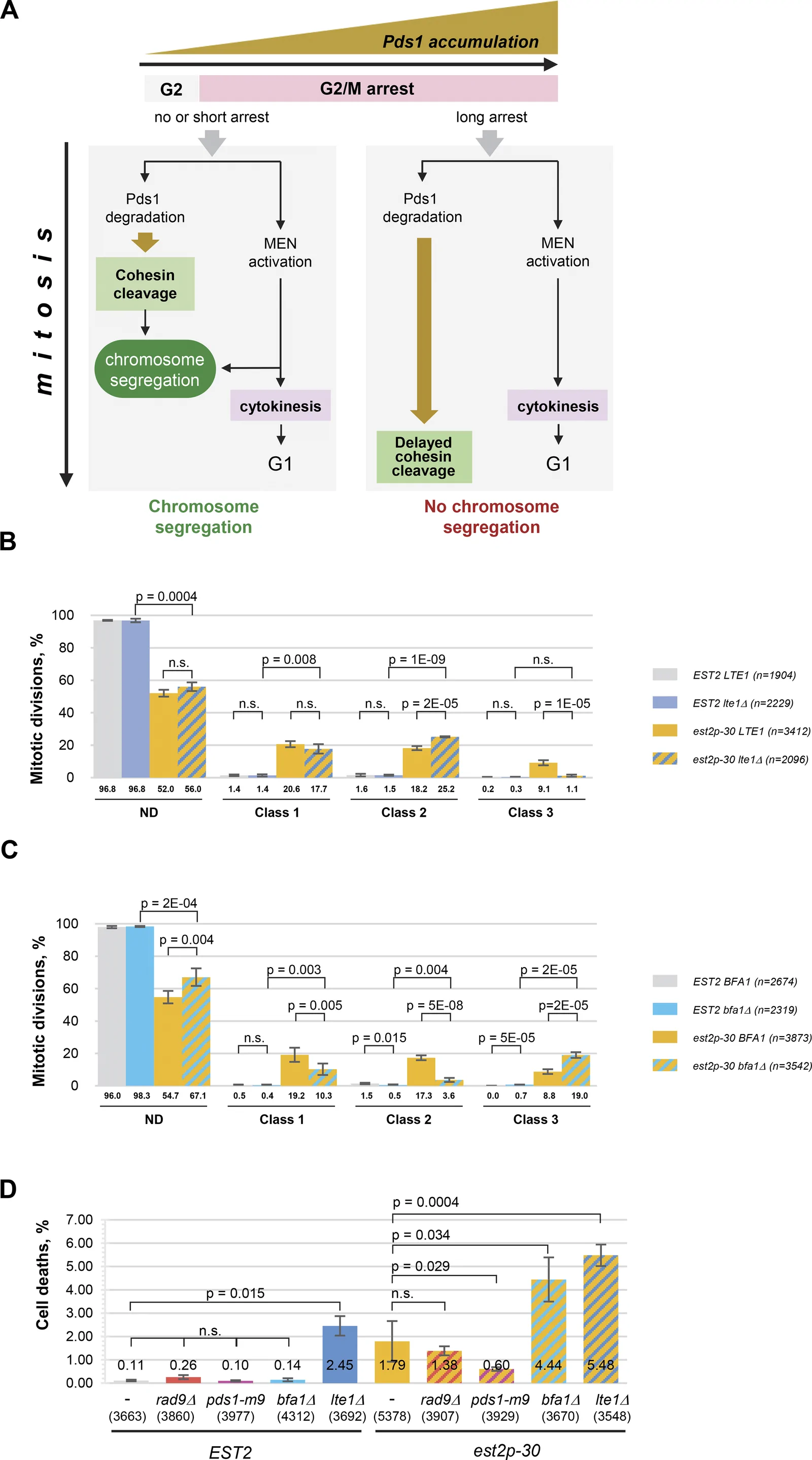
**Activation of MEN affects aberrant mitoses and cell death. (A)** Model for aberrant mitoses. Miscoordinated cohesin cleavage and MEN activation lead to skipped chromosome segregation and an aberrant mitosis (on the right). This miscoordination stems from accumulation of excessive Pds1 during extended G2/M arrests (top), which leads to a delay in completing the degradation of Pds1 and the inability of the cells to segregate chromosomes before cytokinesis takes place. **(B)** Probability of aberrant mitoses in *est2p-30* cells undergoing G2/M arrest depends on *LTE1*. **(C)***BFA1* deletion exacerbates the high frequency of aberrant mitoses in response to TI. **(D)** Frequency of mitotic events resulting in cell deaths analyzed by time-lapse microscopy imaging. In B–D, averages of at least three biological repeats are plotted. The error bars represent standard deviation. A total number of mitotic events (*n*) scored for each genotype in all the repeats is stated in the legend.

Based on the findings presented above, we propose a model where excessive accumulation of Pds1 during extended G2/M arrests results in aberrant cell divisions (Fig. 9 A). Upon metaphase-to-anaphase transition, Pds1 degradation is induced, but because of the Pds1 accumulation during the arrests, it takes much longer than usual to reduce Pds1 to levels low enough to activate the separase Esp1 and allow sister chromatids to separate. Meanwhile, the mitotic entry also leads to activation of the MEN pathway, which governs cytokinesis. Due to the delay in Pds1 removal, cohesin cleavage and cytokinesis become miscoordinated.

If this hypothesis is correct, then manipulating the activation or progress of MEN may affect the probability of aberrant mitoses. To slow down MEN, we constructed TI strains lacking Lte1, an important but not essential activator of the MEN cascade (Falk et al., 2016). Deletion of *LTE1* in the WT cells had no significant effect on the fractions of different mitotic events, but the *est2p-30 lte1Δ* double mutants experienced significantly fewer aberrant mitoses than the single *est2p-30* mutants (Fig. 9 B). Also, the vast majority of these class 3 events belonged to class 3.1 (0.8% of the total of 1.1% in class 3), i.e., a nuclear division was detected, hinting at the possibility of Lte1 also playing a role in determining the outcomes of aberrant mitoses. These results are consistent with the loss of Lte1 delaying mitotic exit and therefore cytokinesis, thereby allowing more time for the degradation of excessive Pds1 and suppressing aberrant mitoses in cells with TI.

**Figure S2.**
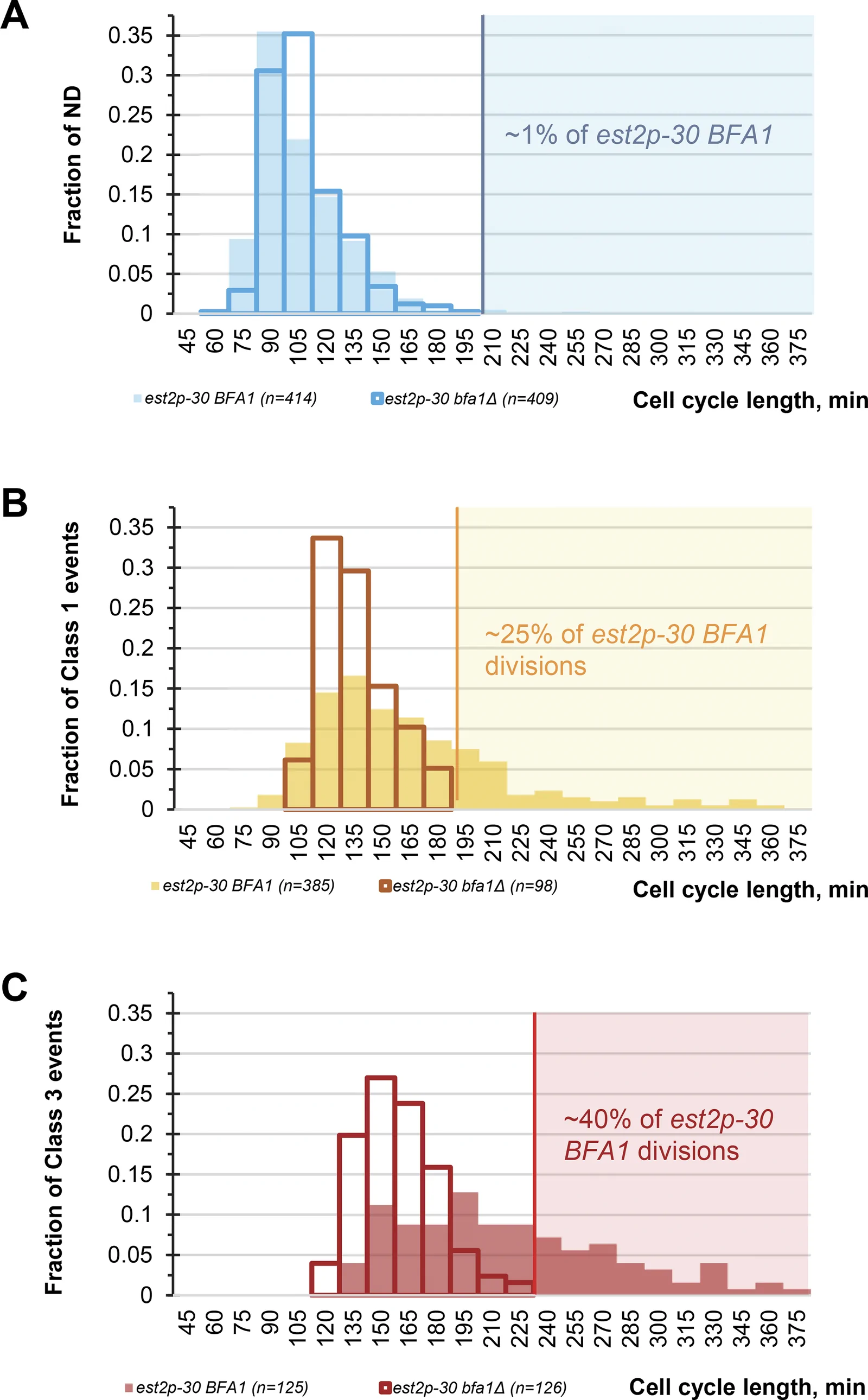
**Loss of Bfa1 shortens cell cycle in cells with TI.** Distribution of cell cycle durations in the populations of *est2p-30 BFA1* and *est2p-30 bfa1Δ* cells analyzed by time-lapse microscopy. **(A–C)** Cell cycles with NDs, class 1 mitoses, and class 3 aberrant mitoses in *HTB2-GFP est2p-30* and *HTB2-GFP est2p-30 bfa1Δ* cells, observed using time-lapse microscopy, were binned according to the cell cycle duration and plotted separately for each class in A–C. **(A)** Comparative analysis of NDs (no G2/M arrest). Only ∼1% of *est2p-30 BFA1* cells have longer cell cycles than the longest cell cycle in *est2p-30 bfa1Δ*. **(B)** Comparative analysis of class 1 divisions. ∼25% of *est2p-30 BFA1* cells have longer cell cycles than the longest cell cycle in *est2p-30 bfa1Δ*. **(C)** Comparative analysis of aberrant mitosis. ∼40% of *est2p-30 BFA1* cells have longer cell cycles than the longest cell cycle in *est2p-30 bfa1Δ*.

It has been reported that the inhibition of MEN through Bfa1 is essential to maintain the G2/M arrest in response to DNA damage signalling due to compromised telomeres (Valerio-Santiago et al., 2013). Consistent with the previous observations on premature MEN activation in cells with telomere damage, the *est2p-30 bfa1Δ* yeast were not able to maintain prolonged arrests (Fig. S2, A–C). More importantly, the frequency of aberrant mitoses in these populations was dramatically increased (Fig. 9 C), consistent with the premature MEN activation promoting aberrant mitoses. Therefore, the cohesin cleavage and MEN activation are highly coordinated, but this coordination is at least partially lost during prolonged G2/M arrests due to excessive accumulation of securin.

We next asked if the mutations that affected the frequency of aberrant mitoses in cells with TI would also have an effect on cell death following cell divisions. Loss of *LTE1* resulted in a significant increase in cell death in the populations with normal telomerase and had an additive effect when combined with *est2p-30* (Fig. 9 D). Loss of *BFA1* did not affect cell death in *EST2* cells but also led to an increase in cell death in the double *est2p-30 bfa1Δ* mutants. Deletion of *RAD9* had no effect on the rates of cell death in either *EST2* or *est2p-30* background. In contrast, *pds1-m9* partially suppressed the phenotype of *est2p-30*. Thus, the genetic relationships between *est2p-30* and the different components of the checkpoints and mitotic progression machineries vary depending on whether chromosome segregation or post-mitotic cell death is analyzed. This could be explained by underscoring chromosome missegregations where some of the class 1 events appearing as equal segregation of chromatin might in fact result in aneuploidy. Because any of the 16 chromosomes in *S. cerevisiae* is essential, missing even a single chromosome would result in cell death.

### Reducing the levels of securin leads to an increase in aneuploidy

**Figure 10.**
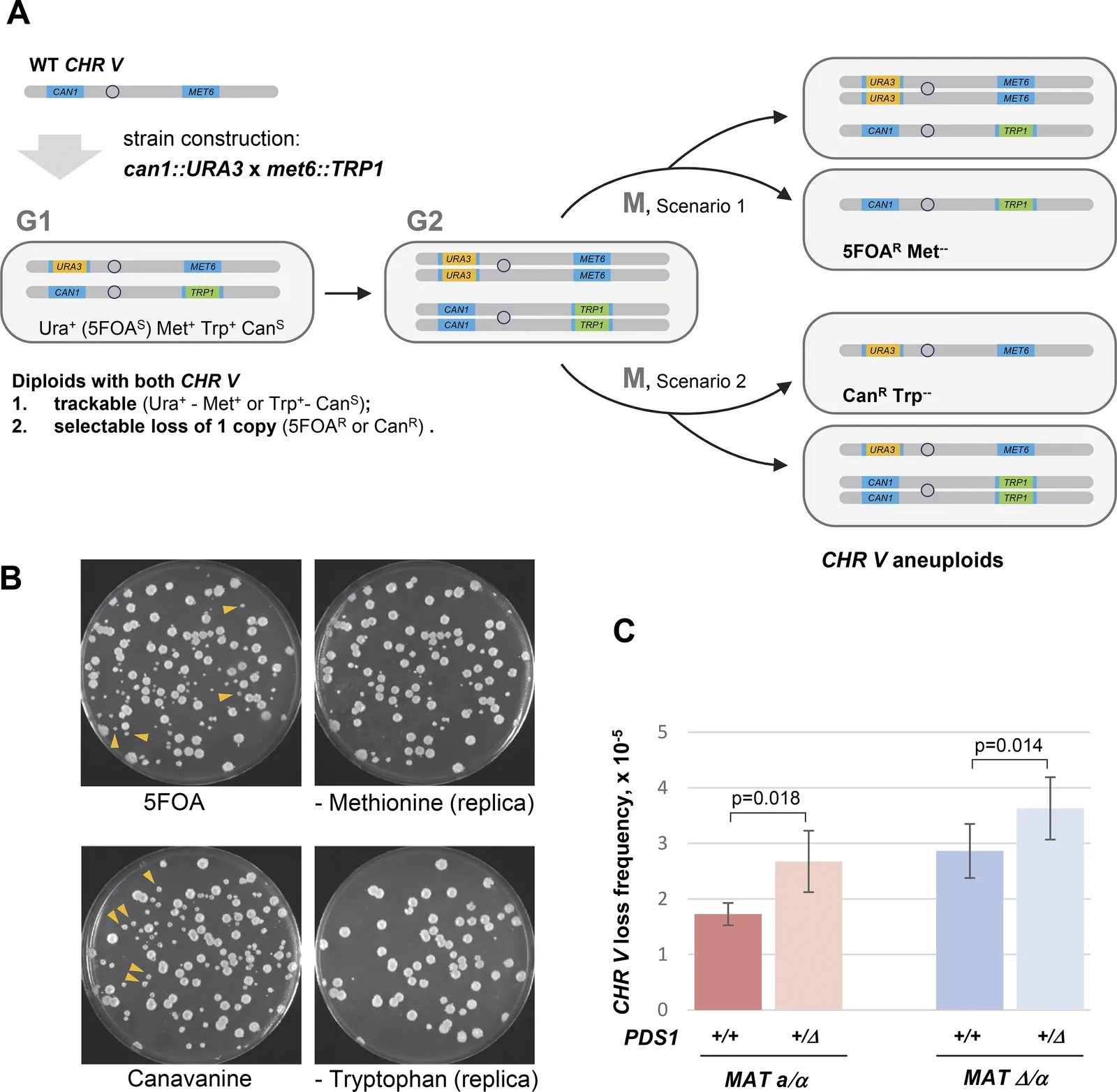
***PDS1* heterozygosity results in an increase in aneuploidy. (A)** Schematic of the experimental setup for quantitative analysis of single chromosome loss in diploid cells. Both copies of *CHR V* were marked with two different genetic markers, one on each arm, in haploid cells of opposite mating type. In postmating diploids, one copy of *CHR V* contained *URA3* and *MET6,* while the other had *CAN1* and *TRP1* at the exact same locations as *URA3* and *MET6,* respectively. A loss of *CHR V* with *URA3-MET6* due to mis-segregation (scenario 1 on the right) results in cells that can be selected on 5-FOA and screened for Met^-^ on plates without methionine. Similarly, a loss of the *CAN1-TRP1* copy of *CHR V* (Scenario 2) leads to canavanine resistance and a Trp^-^ phenotype. **(B)** Representative examples of *CHR V* loss analysis using selection for either 5-FOA–resistant or canavanine-resistant colonies. The former were screened by replica-plating for Met^-^ (top pair of plates), while the latter for Trp^-^ (bottom pair of plates). Notice that *CHR V* aneuploids have a growth defect and appear as small colonies, some of which are indicated with yellow arrowheads. **(C)***PDS1* heterozygosity results in an increase in *CHR V* loss. Averages of at least three biological repeats are plotted for each genotype. The error bars represent standard deviation.

The reduction in Pds1 levels in *pds1p-90* mutants did not cause a detectable increase in class 3 events in cells with WT telomerase (Fig. 6 F). Why have not yeast cells evolved a lower level of *PDS1* expression to avoid aberrant mitoses after prolonged G2/M arrests? One explanation is that it might have come at a higher cost than other types of genomic instability under normal growth conditions, for example, missegregation of single chromosomes due to less tight control of cohesin cleavage. To address this hypothesis, we compared the rates of single chromosome mis-segregation in homozygous WT (*PDS1/PDS1*) and isogenic heterozygous (*PDS1/pds1Δ*) diploids. These experiments were performed in either *MAT a/α* or *MATΔ/α* background. The former is typical of normal diploid yeast, while the latter mimics haploids, where the homologous recombination pathway is significantly less active (Valencia et al., 2001). We constructed a genetic system where the loss of one or the other copy of chromosome V (*CHR V*) could be quantitatively assayed by first selecting for a marker loss using either 5-FOA (loss of *URA3*) or canavanine (loss of *CAN1*) and then screening the postselection colonies for the loss of another marker on the other arm of the same chromosome (Fig. 10, A and B).

**Figure S3.**
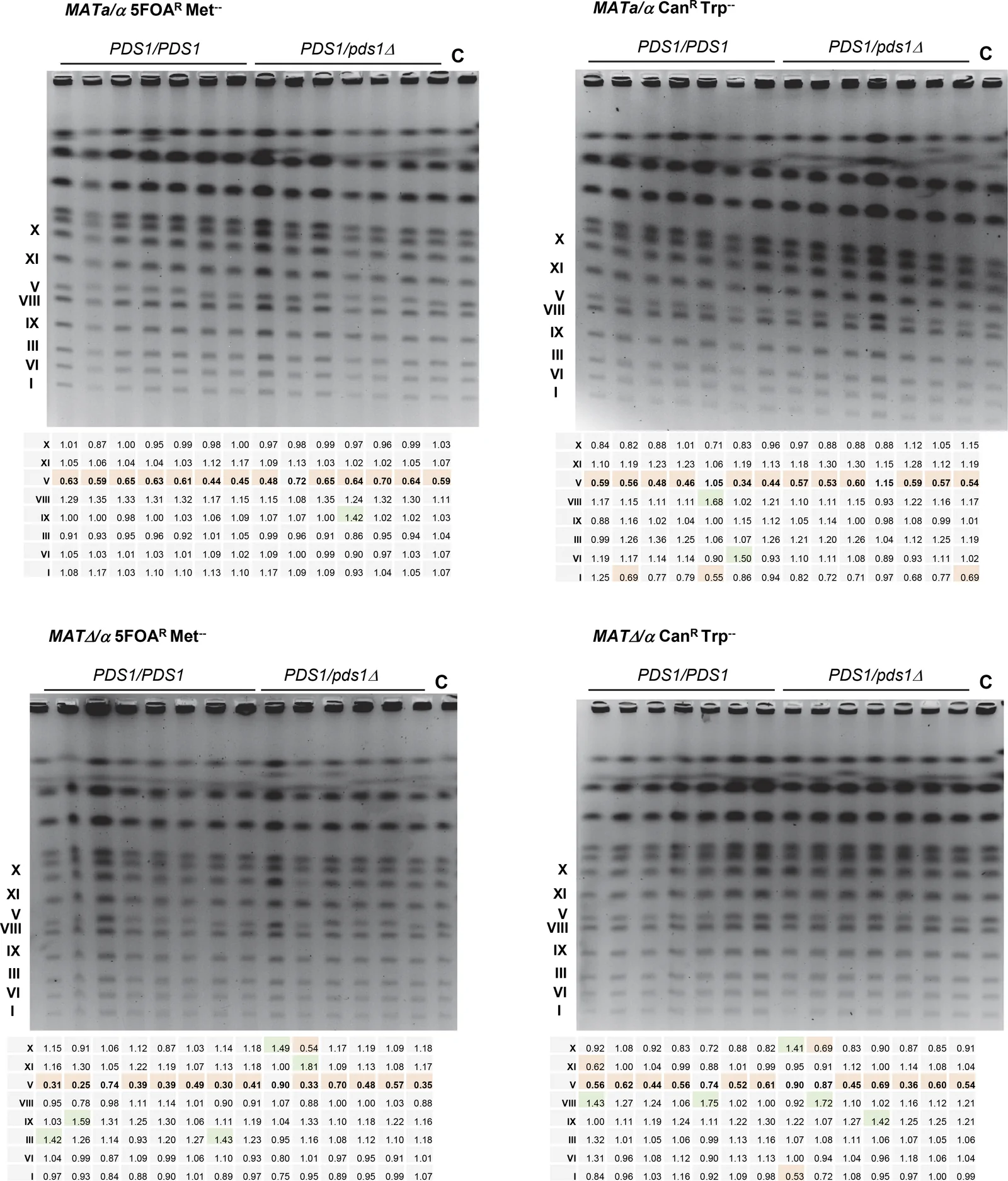
**Karyotype analysis of potential *CHR V* aneuploids by PFGE.** Representative gel images for *PDS1* homozygous and heterozygous diploids, either 5-FOA^R^ Met^--^ or Can^R^ Trp^–^ are shown. An isogenic WT haploid strain was included as a control (C) in the rightmost lane in each gel. The quantification analysis was limited to the eight smallest chromosomes, which are labelled on the left (see Materials and methods for details). The relative DNA signal for each of these chromosomes in the analyzed clones is shown in the table under the gel image. Any relative amount of 0.7 and below (highlighted in light brown) was considered a loss of one of the two copies of a chromosome, and above 1.4 (in light green) was considered a gain of a third copy. Notice that *CHR V* aneuploids are unstable, as gain of a second copy of *CHR V* confers a strong growth advantage. Therefore, some of the analyzed clones were likely mixed populations with some fraction of cells with a re-gained second copy of *CHR V*. This could explain why some of the screened clones showed the relative amount of *CHR V* DNA somewhere between 1 (diploids) and 0.5 (aneuploids). Source data are available for this figure: SourceData FS3.

Lowering the Pds1 levels through heterozygosity resulted in an increase in *CHR V* aneuploidy (Fig. 10 C). Analysis of the karyotypes of the identified aneuploids by PFGE confirmed the loss of *CHR V* in most analyzed clones. The remaining clones might have regained a second copy of the remaining *CHR V* in the process of the experiment, as returning to euploidy conferred a strong growth advantage. Furthermore, the loss of *CHR V* was often accompanied by either gains or losses of other chromosomes in many of these clones (Fig. S3). Therefore, the Pds1 levels might have evolved to be optimal for providing accurate chromosome segregation under normal growth conditions. In this case, the protein accumulation during the G2/M arrest allows the majority of cell divisions to occur normally. It remains to be addressed whether the observed death of the long-arrested cells through aberrant mitoses is collateral damage or an evolutionary product designed to eliminate the cells struggling to repair DNA damage. These cells are the most likely candidates for undergoing genomic instability should they manage to survive DNA damage stress. Eliminating them through aberrant mitoses might be a quality control mechanism preserving genomic stability in microbial populations.

## Discussion

In this study, we described at the single-cell level how activation of DNA damage signalling in response to DSBs and critically short telomeres affects cell cycle progression, in particular genome segregation in mitosis. Different DNA damage inputs leading to activation of an extended G2/M arrest and accumulation of Pds1 increase the probability of chromosome missegregation and cell death.

To investigate cells with critically short telomeres, we constructed a yeast model for TI. In many aspects, this model mimics the developmentally programmed TI in humans and other large mammals, where the gene for telomerase catalytic subunit TERT is downregulated during development (Gomes et al., 2011; Wright et al., 1996). In contrast, yeast express telomerase constitutively. TI in *S. cerevisiae* can be triggered naturally by higher growth temperature, which leads to lower Est2 protein levels (Millet et al., 2015). To avoid pleiotropic effects caused by elevated temperatures, we decreased Est2 by interfering with the *EST2* gene promoter and generated cell populations with TI under the growth conditions normally used in laboratory settings.

Telomerase-deficient cells have no means to repair their dysfunctional telomeres other than through recombination. In contrast, cells with TI can use telomerase, even though it is present at low levels. The time window for telomerase to operate in cells with TI can be extended by a G2/M arrest triggered by critically short telomeres. DNA damage signalling in response to short telomeres also stimulates homologous recombination (Barlow and Rothstein, 2009; Bashkirov et al., 2000; Dion et al., 2012; Flott et al., 2011; Ullal et al., 2011). Recombination might be either regularly or occasionally operating on Y’-telomeres in cells with TI, as evidenced by the amplification of subtelomeric Y’ repeats (Fig. 1, D and E), characteristic of type I telomerase-deficient survivors (Lundblad and Blackburn, 1993). Therefore, the cells with TI might be using both Rad51-dependent recombination and telomerase for telomere maintenance.

A single unrepairable DSB leads to a G2 arrest, which extends the cell cycle duration ∼six times (Dotiwala et al., 2007). Following this long arrest, cells undergo Cdc5-dependent adaptation by dividing in spite of the unrepaired damage (Dotiwala et al., 2007; Sandell and Zakian, 1993; Toczyski et al., 1997). In a similar setting, we detected 1.8% of these divisions as aberrant mitoses (Fig. 5 G), while cells with a subtelomeric break have a much lower frequency of class 3 events, presumably because a one-ended break is not able to generate a strong G2/M arrest. At the same time, *est2p-30* had a higher fraction of aberrant mitoses than the cells with two-ended DSBs, suggesting that the aberrant mitoses may occur in the cells with >2 critically short telomeres per haploid genome (G2/M cells have two sets of chromosomes and therefore 2 two-ended DSBs).

In the cells with TI, we very rarely saw cell cycle duration exceeding 360 min, i.e., only four normal cell cycles of 90 min each (Fig. 5 A). The distribution of aberrant mitoses peaked at around 180 min (Fig. 5 A), i.e., the duration of only two cell cycles, which might be too early to activate adaptation. Therefore, the aberrant mitoses described here might be different from the adaptation-driven divisions in telomerase-deficient cells (Coutelier et al., 2018). Instead of involving adaptation mechanisms, the cells with TI might rely on shutting down DNA damage signalling through successfully extending most of the critically short telomeres by telomerase. In telomerase-negative cells, critically short telomeres persist longer, thereby increasing the chances of aberrant mitoses, the terminal nature of which might be responsible for cell death during senescence of telomerase-deficient cells.

Mitosis is one of the most sophisticated stages of the cell cycle. It is orchestrated by multiple kinases and phosphatases, as well as the anaphase-promoting complex (APC) targeting proteins for degradation. The regulation of Pds1 is known to involve Cdk1 and Chk1 kinases, PP2A^Cdc55^ phosphatase, and APC-directed proteasomal degradation upon cell commitment to mitosis (Agarwal et al., 2003; Khondker et al., 2020; Wang et al., 2001). Pds1 degradation triggers nuclear division, which is coordinated with cytokinesis controlled by MEN. The coordination is carried out by Cdc14 phosphatase and a polo-like kinase Cdc5 (Cohen-Fix et al., 1996; Manzano-López and Monje-Casas, 2020; Shou et al., 2002; Stegmeier et al., 2002). Activation of the DNA damage signalling, which arrests the G2/M progression, adds another level of regulation to this already complicated network, involving Chk1-dependent phosphorylation of Pds1 to block chromosome segregation (Cohen-Fix and Koshland, 1997; Sanchez et al., 1999; Wang et al., 2001). The mitotic progression after longer G2/M arrests does not always adequately adjust to the excess of accumulated Pds1 (Fig. 8). The hypothesis of miscoordination between nuclear division and cytokinesis being the primary reason for these aberrant mitoses (Fig. 9 A) is supported by our genetic manipulations of MEN progression (Fig. 9, B and C).

The aberrant mitoses in cells with TI resemble the terminal genome-level missegregation (GLM) events observed in ageing mother cells in *S. cerevisiae* (Crane et al., 2019). Like class 3 events here, they are Rad9-dependent and characterized by the rDNA region on yeast chromosome XII remaining in the mother cell while the rest of the nucleus is moved to the bud. Yeast cells with terminal GLMs lack Cdc14 release from the nucleolus (Crane et al., 2019). During mitosis, Cdc14 is released in two stages. Although the early Esp1-induced partial release of Cdc14 can be dispensable for mitotic exit (Lu and Cross, 2009), it is required for nuclear positioning, rDNA segregation, spindle stability, and action prior to mitotic exit (D’Amours et al., 2004; Higuchi and Uhlmann, 2005; Ross and Cohen-Fix, 2004; Sullivan et al., 2004). Accumulation of Pds1 during prolonged G2/M arrests in cells with TI, DSBs, and possibly in ageing mother cells would prolong Esp1 inhibition and delay or even abolish the Esp1-dependent release of Cdc14 from the nucleolus and might result in a mitotic exit accompanied by genome missegregation. Therefore, the mis-regulation of the cohesin-independent function of Esp1 might contribute to the cause of aberrant mitoses.

Class 3 mitotic events lead to cell death. This cell death might result from losing rDNA and/or some essential genes in close proximity to the rDNA array on chromosome XII in yeast. While a 10-fold reduction in the number of rDNA repeats, from ∼150 copies normally present in yeast down to 15, is compatible with cell survival (Iida and Kobayashi, 2019; Kobayashi, 2006), loss of the whole rDNA array would be lethal. After undergoing an aberrant mitosis, cells might also be missing other essential genetic material or dealing with unrepairable DNA damage. The disappearance of Htb2-GFP signal prior to cell death (Fig. 3, C and D) can be explained by degradation of histones previously observed in aged mother cells and in response to DNA damage (Crane et al., 2019; Hauer et al., 2017). Another explanation for the cell death might be activation of a pathway where Pds1 accumulation might be only one of the steps in a more complex mechanism such as apoptosis. Both Pds1 and Esp1 have been linked to the regulation of H_2_O_2_-induced apoptosis in yeast (Yang et al., 2008), but it is unclear if this mechanism might be relevant to the cell death described in this study.

The DNA damage response functions to suppress genomic instability by allowing cells to pause the cell cycle until the repair is complete. This mechanism has an important additional level of regulation in multicellular eukaryotes, where the inability to complete repair or deal with other stresses in a timely manner can trigger one of the two developmental programs, either apoptosis or senescence, to eliminate cells with an increased potential for instability (reviewed by Childs et al., 2014; Childs et al., 2017). Our experiments suggest that unicellular organisms might use a similar strategy to preserve genome integrity in their populations. The probability of an aberrant mitosis becomes over 50% if a G2 arrest extends the cell cycle to 200 min and further increases at even longer arrests (Fig. 5 B). Such a high probability of aberrant mitoses eventually leading to cell death would be an efficient enough mechanism to eliminate most cells struggling with DNA repair and therefore being at a higher risk of acquiring mutations. Even though these mutations could result in a growth defect in haploids, thereby limiting their spreading in the populations, mating with a healthy growing cell might allow the mutations to persist in a heterozygous state. Therefore, a mechanism involving Pds1 accumulation might have evolved to safeguard genome stability in the populations of unicellular eukaryotes.

Pds1 might operate similarly to the recently discovered p53–53BP1–USP28 mitotic stopwatch in human cells, which works via stabilization of p53 (Meitinger et al., 2024). The p53–53BP1–USP28 complex “monitors” the duration of M-phase and triggers irreversible G1 arrests after prolonged mitoses. The Pds1-dependent pathway might be similar to the p53 mitotic stopwatch in how these two mechanisms are built—a sensor protein, Pds1 in yeast or p53 in mammals, is stabilized, accumulates over time, and its levels are then used by the cell as a readout of time. The biological roles of these pathways are even more similar: they both eliminate potentially unstable cells to preserve genome integrity.

Lowering the *PDS1* gene expression levels partially suppresses the aberrant mitoses after prolonged G2/M arrests (Fig. 6 F), but at the cost of aneuploidy under normal growth conditions (Fig. 10 C). These data argue that evolution might have kept the Pds1 levels high enough to maximize the genome stability in unchallenged cells and that the Pds1 levels are finely tuned to minimize possible aneuploidy in the absence of stress and then provide enough range during the G2/M arrests to differentiate between the cells with shorter and longer arrests.

Despite the very frequent cell death following the class 3 events, there is a small chance for a cell to survive an aberrant mitosis and preserve its proliferation potential (Fig. 3 E, class 1). Such cells would end up undergoing diploidization, similar to tetraploidization in higher eukaryotes following a mitotic slippage in response to compromised telomeres (Davoli et al., 2010) Polyploidization increases cells’ ability to adapt through aneuploidy by allowing them to reduce the number of some particular chromosomes to rebalance the genome to suit a particular adaptation. This is commonly found in cancers (Gordon et al., 2012) and has also been observed in yeast (Chen et al., 2012; Millet et al., 2015). Therefore, surviving mitotic slippage could be advantageous for adaptation of microbial populations to some stresses and might play an important role in microbial pathogenesis, where ploidy change and aneuploidy are very common (Gilchrist and Stelkens, 2019; Todd et al., 2017). The single-cell approach allows one to account for the stochasticity factor in modelling the behavior of cell populations without overlooking infrequent events, such as aberrant mitoses, as often they are the ones with the highest impact on how populations adapt to stress and evolve over time.

## Materials and methods

### Yeast strains, oligonucleotides, and plasmids

Yeast strains, oligonucleotides and plasmids used in this study are described in Table S1. To construct strains with TI, pFA6a-*hphMX6* (see Table S1, section C) was used as a PCR template in combination with OSM3068 as an R1 primer (*EST2* -30) and OSM2693 (*EST2 -30*), OSM3069 (*EST2 -20*), OSM3168 (*EST2 -17*), OSM3169 (*EST2 -23*), or OSM3170 (*EST2 -27*) as an F1 primer (see Table S1, section B for oligonucleotide sequences).

### Cell passaging

Cells were grown on YPD agar with additional 0.01% adenine at 30°C for 48 h. Yeast from multiple colonies were scooped with a toothpick and repassed onto a fresh plate for growing the next passage as described above. Plates with grown yeast were stored at 4°C. To purify genomic DNA from different passages at the same time, cells were scooped from each of the stored plates and patched onto a fresh plate. Cells from the fresh patches grown overnight at 30°C were scraped with a toothpick and used for DNA purification.

### Population doubling time measurement

Cultures in YPD medium were prepared by resuspending cells from freshly grown patches and grown overnight (at least 8 h) to log-phase (OD_600_ < 0.6). The log-phase cultures were diluted using pre-warmed YPD broth to OD_600_ of 0.025. 200 μl of the resultant culture was transferred into a well of a sterile 96-well plate. Every analyzed genotype had at least two biological and two technical repeats. The optical density was measured in a Tecan Infinite M200 plate reader. The absorbance curves were analyzed using the Omniplate software (Montano-Gutierrez et al., 2022).

### Telomere analysis by Southern blotting

Telomere length and structure were analyzed by telomere-specific Southern blotting. Purified yeast genomic DNA was digested with XhoI, resolved on a 0.7% agarose gel in 1×TBE, and transferred onto a nylon membrane. The membrane was hybridized to a ^32^P-radiolabeled telomeric oligo (CCCACA)_4_ to detect internal Y’ repeats and terminal restriction fragments.

### Western blotting

Protein extracts for Est2-13MYC, Pds1-13MYC, and actin western blotting were resolved in an SDS polyacrylamide gel and transferred onto PVDF transfer membranes (Immobilon-FL, 0.45 μm pores, IPFL00005; Merck Millipore Ltd.). Anti-c-Myc (9E10) mouse monoclonal (13-2500; Thermo Fisher Scientific, 1:1,000 dilution) was used to detect Est2-13Myc and Pds1-13Myc. Anti–β-actin mouse monoclonal antibody (ab8224; Abcam, 1:50,000 dilution) was used for Act1 western blotting. Goat anti-mouse IgG (H + L) cross-adsorbed secondary antibody Alexa Fluor 680 (A-21057; Thermo Fisher Scientific, 1:12,500 dilution) was used with the corresponding primary antibodies. Western blotting membranes were scanned using Odyssey CLx fluorescent scanner (LI-COR). The resulting images were analyzed using Image Studio Lite software.

### Time-lapse and fluorescence microscopy

Imaging of live cells was carried out using a NikonTi2 inverted microscope equipped with a 100× 1.49 NA CFI Plan Apochromat TIRF objective, Lumencor Spectra X light source (Lumencor) and a Photometrics Prime 95B camera (Teledyne Photometrics) using the NIS-Elements software. Filter sets from Semrock (Semrock) were used to image GFP at excitation 474/26 nm, emission 525/40 nm, mRuby2 and tdTomato at excitation 543/23 nm, emission 595/31 nm, and mCherry at excitation 578/21 nm, emission 641/75 nm. All experiments were performed in an Okolab Environmental chamber pre-warmed at 30°C.

In experiments to analyze the frequency of different mitotic events using chambers, yeast cultures were pre-grown in SC complete medium for at least 8 h so that their OD_600_ did not exceed 0.6. Approximately 0.02 OD of the cell culture was added by pipetting into each well of the Ibidi chamber, pre-coated with concanavalin A. The images were taken every 5 min for at least 4 h in the bright-field and GFP channels with 5 Z-stacks of 0.6 μm. The GFP channel was set to a 30 ms exposure time and 5% light source intensity.

We used agar pads to analyze cell area distribution. Agar pads were made by moulding the YPD liquid medium with 1% agarose in 65 μl Gene Frames. Yeast pre-grown in YPD were spotted on an agar pad, and the sample was sealed with a coverslip. Multiple photos were taken in the bright-field and GFP channels with 5 Z-stacks of 0.6 μm.

We also used agar pads to analyze the mitotic behavior of cells with Myo1-mCherry (125 ms exposure, 5% intensity), Spc42-tdTomato (125 ms exposure, 5% intensity), Nop56-mCherry (150 ms exposure, 8% intensity), mRuby2-Tub1 (tdTomato at 400 ms exposure, 5% intensity), and Pds1-tdTomato (500 ms exposure, 5% intensity). The images were taken every 5 min for at least 7 h in the bright-field, GFP, and mCherry/tdTomato channels with 5 or 7 Z-stacks of 0.6 μm.

Agar pads were also used to analyze the mitotic behavior of cells treated with phleomycin. Cell cultures were grown in YPD at 30°C in a shaking incubator for at least 8 h until the OD_600_ reached 0.3. Phleomycin was added to the cultures at the desired concentrations, and the cultures were left in the shaking incubator for another hour before being spotted on agar pads. The agar pads contained the same concentration of phleomycin as the treatment in the liquid culture. The images were taken every 5 min for at least 7 h in the bright-field and GFP channels with 5 Z-stacks of 0.6 μm. The GFP channel was set to a 30 ms exposure time and 5% light source intensity.

### Image analysis and single-cell lineage tracking

The images were analyzed using Fiji (ImageJ) software. Cell area was measured for all cells with clear borders in the focal plane. Cell borders excluding the bud cell compartment were marked using the polygon selection tool, and the area within was measured.

In time-lapse imaging, the mitotic behavior of each cell in the focal plane was observed and categorized. A cell was considered undergoing an arrest if the majority of the DNA was transferred and localized in the bud for more than a single time frame (5 min). After that, the observed cell division with the arrest was assigned to classes 1, 2, or 3, depending on the division outcome. The divisions completed after an arrest without any visible defect were defined as class 1. Class 3 events were aberrant mitoses with an obvious defect in nuclear segregation. We observed splits of chromatin masses (transient or permanent) inside either a mother or a daughter cell compartment followed by re-budding of the cell with the majority of the DNA. These divisions were called class 3.1 events. There were also cells generating an anucleate cell and a re-budding cell with the majority of the DNA without a prior nuclear division detected. These were called class 3.2 events. Any division generating >2 separated chromatin masses was assigned to class 3.3 events. Cells remaining arrested by the end of the recorded imaging were registered as class 2 events.

Fiji (ImageJ) software was used for quantitative analysis of Pds1-tdTomato levels throughout the cell cycle. The mean fluorescence intensity and standard deviation of the tdTomato signal were measured within the nucleus, which was defined by Htb2-GFP signal. The background signal of the non-fluorescent area outside the cell was subtracted from the mean fluorescence intensity.

### Computational and statistical analyses

All the raw data on scoring different types of mitotic events are summarized in the supplementary Excel file. Equal or unequal variance of the Student’s two-sample two-tailed *t* test was used to compare two groups, when appropriate. For multiple group comparison, one-way ANOVA was used with the appropriate post hoc test: the Tukey–Kramer test or the Games–Howell test. p values were calculated using StatsCalculators Software from StatsCalculators Team (www.statscalculators.com). p values were indicated in each figure. p values >0.05 were presented as n.s., which stands for nonsignificant.

### Quantitative analysis of *CHR V* loss

Diploid strains with both arms of *CHRV* marked genetically, *CAN1_met6::TRP1/can1::URA3_MET6 ura3-52/ura3-52 trp1-289/trp1-289*, were streaked to single colonies on synthetic agar media without uracil and tryptophan. Single colonies grown for 48 h were patched onto YPD agar and incubated for 24 h at 30°C. Patches were resuspended in water, and appropriate serial dilutions were plated on YPD plates to determine the cell titer, on plates with 5-FOA to select for colonies with lost *URA3*, and on plates with canavanine to select for a loss of *CAN1*. Colonies on YPD plates were scored in 2 days. In 3 days after plating, colonies grown on the agar with the drugs were replica-plated from 5-FOA onto plates without methionine and from canavanine onto plates without tryptophan. 5-FOA^R^ Met^-^ and Can^R^ Trp^-^ colonies were scored as aneuploid. The frequency of aneuploidy was calculated as the ratio of aneuploids (5-FOA^R^ Met^-^ and Can^R^ Trp^-^ combined) to the cell titer.

### Pulse-field gel electrophoresis of yeast chromosomes

The karyotypes of the cells selected for the loss of *CHR V* using the genetic markers were analyzed by pulse-field gel electrophoresis as described previously (Hage and Houseley, 2013). Colonies from the selective plates (5-FOA or CAN) were purified on YPD plates. A smaller colony characteristic of aneuploid phenotype was selected from each purification and patched on YPD overnight. A patch of a control WT haploid strain was included in each set of samples analyzed on the same gel. The next day, the cells were washed with 1 ml of Wash Buffer WB (10 mM Tris-HCL, pH7.6, 50 mM EDTA) and resuspended in 100 μl of WB with 17 U of Lyticase (L2524; Sigma-Aldrich). The suspension was mixed with 100 μl of 1.6% agarose (Seakem LE) pre-warmed to 50°C and transferred into the plug mould. Agarose plugs were incubated with 500 μl of the Lyticase solution (170 U of Lyticase in 500 μl of WB) for 1 h at 37°C and then with 500 μl of Proteinase K solution (100 mM EDTA, 0.2% SDS, 1% N-laurylsarcosine sodium, and 1 mg/ml proteinase K) overnight at 50°C. The next day, the plugs were washed with 1 ml of WB for 30 min at room temperature with gentle agitation; the wash was repeated three times. Half of each plug and the Yeast Chromosomes PFG marker (N0345S) were attached to a comb and placed into the gel casting tray. 1% agarose gel (Certified Megabase Agarose, Bio-Rad) was poured over the plugs and polymerized at 4°C; the comb was gently extracted. Chromosomes were resolved using the CHEF-DR III electrophoresis system (Bio-Rad) in 0.5x TBE at 6 V/cm and 14°C with the following current switch settings: 120° every 60 s for 16 h, followed by 120° every 90 s for 11 h. After the run was finished, the gel was stained with ethidium bromide and imaged using a Gel Doc XR+ imaging system (Bio-Rad).

For the analyses of aneuploidy (only *CHR X, XI, V, VIII, IX, III, VI*, and *I*), the signal in the band of each chromosome in the gel was normalized against the same chromosome in the control haploid strain, and the resultant values for the eight analyzed chromosomes were used to calculate an average for the normalized values for each strain. Then, the normalized value for each chromosome was divided by this average to calculate the relative increase/decrease in the EthBr staining for each chromosome. The values often deviated from a clear indication of a chromosome gain or loss, perhaps because the populations of aneuploid cells are highly unstable. Reverting back to euploidy often confers a growth advantage and therefore at least some analyzed clones might be mixed populations of cells with different karyotypes.

### Online supplemental material

Fig. S1 shows the effect of lig4D on aberrant mitoses in cells with TI. Fig. S2 demonstrates how loss of *BFA1* affects cell cycle duration in *est2p-30* cells, separately for each class of mitotic events. Fig. S3 presents analysis of karyotypes by PFGE in CHR V aneuploids isolated from WT and *PDS1/pds1Δ* diploids. Table S1 summarizes yeast strains, oligonucleotides, and plasmids used in the study. Data S1 shows the raw data on counting and classification of all the mitotic events analyzed using time-lapse microscopy, summarized in the supplementary Excel file, with a spreadsheet per each plot presented in the article.

## Supplementary Material

Review History

Table S1 shows yeast strains, oligonucleotides, and plasmids used in this study.

Data S1 shows the raw data on counting and classification of all the mitotic events analyzed using time-lapse microscopy with a spreadsheet per each plot presented in the article.

SourceData F1 is the source file for Fig. 1.

SourceData F4 is the source file for Fig. 4.

SourceData F6 is the source file for Fig. 6.

SourceData FS3 is the source file for Fig. S3.

## Data Availability

Data are available in the article itself and its supplementary materials.
