# Supplementary material for: Extended DNA damage–induced G2/M arrests lead to aberrant mitoses through accumulation of securin
Source: J Cell Biol. 2026 Nov 2;225(12):e202511106. doi: 10.1083/jcb.202511106 (PMC13726689; doi:10.1083/jcb.202511106)
Supplement: Supplementary file 1 [file jcb_202511106_review_history.pdf]

Extended DNA damage-induced G2/M arrests lead to aberrant mitoses through accumulation of securin

Anton Dubenko, Marah Jnied, Oleksii Kotenko, and Svetlana Makovets

Corresponding Author(s): Svetlana Makovets, University of Edinburgh

Review Timeline:

Submission Date:	2025-11-15
Editorial Decision:	2025-12-04
Revision Received:	2026-06-03
Editorial Decision:	2026-07-29
Revision Received:	2026-08-11
Editorial Decision:	2026-08-21
Revision Received:	2026-09-01
Accepted:	2026-09-09

Monitoring Editor: Daniel Lew

Scientific Editor: Therese Gerbich

Transaction Report:

(Note: With the exception of the correction of typographical or spelling errors that could be a source of ambiguity, letters and reports are not edited. The original formatting of letters and referee reports may not be reflected in this compilation.)

DOI: https://doi.org/10.1083/jcb.202511106

Revision 0

Review #1

1. Evidence, reproducibility and clarity:

Evidence, reproducibility and clarity (Required)

In their paper, Dubenko et al. investigated how cells respond to DNA double-strand breaks and telomere dysfunction at the single-cell level using time-lapse microscopy in budding yeast. To model telomerase insufficiency (TI), they weakened the EST2 promoter, mimicking partial telomerase downregulation. This led to shortened but stably maintained telomeres and DNA damage responses. They also analyzed est2Δ mutants and phleomycin-treated cells to corroborate their findings. DNA damage caused highly variable G2/M arrest durations, with prolonged arrests frequently leading to aberrant mitoses characterized by mis-coordination of nuclear division and cytokinesis, nucleolar loss, and eventual cell death. The underlying mechanism involved Chk1-dependent phosphorylation of securin (Pds1), whose progressive accumulation delayed or blocked nuclear division. Reducing PDS1 expression partially suppressed aberrant mitoses after prolonged G2/M arrest, but at the cost of increased aneuploidy under normal growth conditions. Similarly, deletion of LTE1, which delays mitotic exit and cytokinesis, reduced aberrant mitoses by allowing more time for Pds1 degradation. The authors conclude that progressive Pds1 accumulation drives aberrant mitoses, thereby eliminating cells with prolonged damage responses - a potential quality control mechanism that preserves genomic stability in yeast. They further suggest that this may parallel the p53-53BP1-USP28 mitotic stopwatch in human cells, which promotes p53 stabilization to eliminate genomically compromised cells.

Major comments

- Aberrant mitoses occur after DNA damage mediated cell cycle arrest. Following points would help to understand the nature these aberrant mitoses: Do the aberrant mitoses occur after DNA repair or instead arise from slippage out of arrest? Is there a correlation between the duration of DNA damage mediated cell cycle arrest and the different classes

of aberrant mitoses? For example, do longer arrests result in class 3.1 events, while relatively shorter arrests lead to class 3.2 events?
- The data shown in Figure 5 need quantification. What are the frequencies of the events shown? What is the timing between actomyosin ring contraction and chromosome segregation? When chromosomes segregate after prolonged arrest, do the separated nuclei always appear in the bud?
- The authors show that Securin accumulates during prolonged arrest. Securin accumulation suggests Separase inactivation and thus failure of Cohesin cleavage. However, some cells still separate their DNA, suggesting that Cohesin is cleaved in at least a fraction of cases. What percentage of cells experiencing G2/M arrest show Securin accumulation? Do all cells that undergo aberrant mitosis have high levels of Securin? Is this the predominant phenotype? Do cells that separate their DNA after a long DNA damage dependent cell cycle arrest actually degrade Securin or somehow escape the Securin barrier manage to separate their DNA?
- The authors state that short spindles collapse during class 3.2 mitotic events. What is the cell cycle stage here; pre- or post-anaphase? Are sister chromatids separated? In other words, does the failure of nuclear division stem from defective spindle elongation or from failure of cohesin cleavage? Since Esp1 (separase) is also a midzone protein involved in spindle midzone assembly,and is also critical for CdcFourteen-Eary-Anaphase Release (FEAR) it would be important to clarify this point.

Minor comments

- The link between the prolonged DNA damage arrest and aberrant mitoses here seems to be the Pds1 phosphorylation by Chk1, and consequent Pds1 stabilization. However, I feel that this finding is diluted in the results section. A change in the data presentation order may help bringing the result forward.
- The text and main figure (Figure 2) explaining the classes of aberrant mitoses are difficult

to follow. Representative time-lapse image series and graphs from Figure S1 are more complete and easier to interpret, and could be moved into the main figure instead.
- Dark orange bars in Figures 3A and 3F are not indicated in the legend. These type of graphs are also difficult to read due to overlapping data series. It would be better to represent the data in a different way- ie. By showing individual data series separately.
- The data presented in Figure 3B are not clearly explained.
- Striped or dotted graph fill patterns should be avoided, as they are barely visible, especially in legends.
- The discussion could be more concise.
- One-way ANOVA should be used when comparing more than two samples.
- Nuclear divisions occurring within one cell compartment (Figure 5) resemble failures of the spindle position checkpoint (SPOC), which normally inhibits mitotic exit and cytokinesis when spindle elongation into the bud fails. This checkpoint arrests cells in M phase. Interestingly, the most downstream SPOC component (Bfa1) is also required for cell cycle arrest in response to telomere dysfunction. This raises the question of whether Bfa1 is required to arrest these cells with compromised telomeres in G2/M or M phase. Could the authors elaborate on this?

2. Significance:

Significance (Required)

This study will be of interest to researchers working on DNA damage response, cell cycle regulation, telomere biology, and genome stability. It may also engage broader audiences in aging, cancer biology. The yeast telomerase insufficiency (TI) model developed here is a valuable tool for mimicking mammalian cells in which expression of the telomerase catalytic subunit TERT is downregulated during development. Conceptually, the study highlights parallels between yeast and mammalian systems, suggesting evolutionary conservation of quality-control mechanisms that eliminate compromised cells.

Reviewer expertise: Cell cycle control, checkpoints.

3. How much time do you estimate the authors will need to complete the suggested revisions:

Estimated time to Complete Revisions (Required)

(Decision Recommendation)

Between 3 and 6 months

4. *Review Commons* values the work of reviewers and encourages them to get credit for their work. Select 'Yes' below to register your reviewing activity at Web of Science Reviewer Recognition Service (formerly Publons); note that the content of your review will not be visible on Web of Science.

Web of Science Reviewer Recognition

Yes

Review #2

1. Evidence, reproducibility and clarity:

Evidence, reproducibility and clarity (Required)

In this manuscript, the authors analyse the effects of a decrease in telomerase in budding yeast S. cerevisiae on G2/M cell arrest. Using different immunofluorecent tools and time lapse microscopy together with different genetic approaches, they show that telomere insufficiency correlates with cytokinesis problems together with accumulation Pds1 resulting in increased aneuploidy. They conclude that longer arrests may eliminate genetically unstable cells.

The consequences of telomerase deficiency have been extensively studied (work by the labs of Teixeira, Lingner, Shore, Zakian, Wellinger, etc). At the single-cell it has been already shown that senescence varies considerably from one cell to another, that cells can switch from rapid proliferation to a prolonged cell cycle in a single division, and that long cell cycles precede cell death (Xu et al 2014, Coutelier, et al 2018).

This work aims to explore the results of a decrease in telomerase expression and to study the mechanisms responsible for cycle defects. In this sense, this work deserves publication, but at this stage, the manuscript needs to be improved at many levels to make

it appropriate for publication despite the impressive large number of experimental data. The article is quite dense and could be made easier to the reader by clearly highlighting the novelty brought to the field. A way to consider could be to focus on some aspects of the different data, or even considering splitting the data in two publications?

Below are a few points to consider strengthening the impact of this work.

1. The authors use an unusual method in yeast where conditional promoters (GAL, tetO2, CUP1) are well controlled. As a result, the position of the Hygromycin cassette in the EST2 promoter must be improved in the text and figure. Above all, expression levels are not visible on the Western blot, must be quantified by RT-qPCR, and must be shown for all constructs. (both expression and growth). In Fig. 1C, it appears that the actin control comes from a different gel, which should be avoided. In Fig.1F how is cell size calculated?
2. The estimated probabilities of class 3 events assume that a class 2 event becomes either a class 1 event or a class 3 event, but do not consider the fact that class 2 events can also lead to cell death. How do the authors account for this bias? Can the authors explain why they chose to perform 5 Z-stacks of 0.6 μm without projection? Z-projection imaging might be more accurate for the analyses.
3. The conclusion of the experiments in Fig. 3 is that DNA damage and decreased expression of est2 (if well documented, see comment 1) are comparable. Suppression by Δ rad9 must be shown in both cases. To confirm that G2/M arrest is due to DNA damage, analysis of H2A phosphorylation or Rad53 phosphorylation would be beneficial. The conditions for phleomycin treatment must be specified (time). The graphs in Fig. 3A, 3B and 3F could be made easier to understand. What are the percentages calculated on?
4. The conclusions concerning Pds1 should be interpreted with caution, as it has not been proven that aberrant mitoses are caused by the accumulation of Pads1; only a correlation has been demonstrated. We propose to add control strains (WT, Δest2, WT treated with phleomycin). The measurement should be performed on more than 15 cells, and the method should be explained in the Materials and Methods section. Finally,accumulation

should be quantified by RT-qPCR to consolidate the results.
5. The loss of coordination between nuclear division and cytokines is interesting but needs to be supported by quantification and controls (WT, Δest2, MEN mutants).

Minor comments

The authors should make an effort to cite recent work in the field and position their current analysis accordingly, and make sure references are not missing (for instance type I and type II survivors). It would be helpful to also improve the concision and the clarity of the results section, with concluding sentences for each result.
Work could be done on the figures to simplify them. In particular, the bar charts are quite complicated to understand given all the mutations that have been made and the fact that they are each subdivided into classes 1, 2, and 3.

2. Significance:

Significance (Required)

This work aims to explore the results of a decrease in telomerase expression and to study the mechanisms responsible for cycle defects. In this sense, this work deserves publication, but at this stage, the manuscript needs to be improved at many levels to make it appropriate for publication despite the impressive large number of experimental data. The article is quite dense and could be made easier to the reader by clearly highlighting the novelty brought to the field. A way to consider could be to focus on some aspects of the different data, or even considering splitting the data in two publications?

Specialized audience.

Our expertise in DNA repair and yeast genome organization allows us to offer what we hope are constructive comments on this work, even though not telomere specialists.

3. How much time do you estimate the authors will need to complete the suggested revisions:

Estimated time to Complete Revisions (Required)

(Decision Recommendation)

Between 3 and 6 months

4. *Review Commons* values the work of reviewers and encourages them to get credit for their work. Select 'Yes' below to register your reviewing activity at [Web of Science Reviewer Recognition Service]() (formerly Publons); note that the content of your review will not be visible on Web of Science.

Web of Science Reviewer Recognition

Yes

Review #3

1. Evidence, reproducibility and clarity:

Evidence, reproducibility and clarity (Required)

In this manuscript, Dubenko and colleagues investigated, at a single-cell level, how yeast cells regulate cell cycle arrest and recovery in response to DNA breaks or telomere dysfunctions, as well as the potential outcomes for cells that resume cell cycle progression after arrests of varying durations. They used Saccharomyces cerevisiae populations in which telomere defects were induced by downregulating telomerase expression, while DNA breaks were generated through phleomycin treatment. Time-lapse microscopy was employed to measure the duration of checkpoint-induced cell cycle blocks, nuclear division, and the localization of key proteins and structures in individual cells, with the goal of exploring heterogeneity in the DNA damage response and the relationship between cell cycle arrest and chromosome stability.

To monitor nuclear dynamics, the authors generated yeast strains with reduced telomerase expression carrying GFP-tagged histones and mCherry-labeled Nop56 for time-lapse imaging. Both telomerase downregulation and phleomycin treatment led to an increased frequency of aberrant DNA segregations following G2/M cell cycle arrests. This frequency was higher in cells experiencing prolonged arrests. Elevated levels of Pds1 were observed in cells with extended checkpoint activation, whereas partial loss of Pds1 function reduced

the frequency of abnormal segregations but increased the incidence of aneuploidy. Analysis of mitotic spindle dynamics through time-lapse microscopy indicated that the spindle becomes unstable in cells undergoing prolonged cell cycle arrest. Furthermore, mutations that delayed cytokinesis decreased the occurrence of aberrant mitoses, while mutations promoting premature activation of MEN had the opposite effect, increasing aberrant mitoses.

Overall, the authors concluded that sustained activation of the DNA damage checkpoint leads to mitotic defects, aneuploidy, and cell death over a few divisions in cells that take too long to repair DNA damage. They proposed that this mechanism could eliminate cells with unstable genomes.

Major comments:

The data and the methods presented in such a way that they can be reproduced.

The experiments are adequately replicated and statistical analysis adequate.

Most of the key conclusions are convincing, particularly those concerning the relationship between prolonged cell cycle arrest and mitotic defects, the role of Pds1, and the coordination between chromosome segregation and mitotic exit/cytokinesis.

However, while the authors propose that prolonged arrests and mitotic defects may occur in cells that take too long to repair DNA damage, they did not directly assess DNA damage persistence.

Another important point that, in my opinion, need further investigation is the impact of EST2 downregulation on cell growth kinetics and telomere integrity.

The extent of telomerase downregulation, as well as the persistence of telomerase activity in TI strains, was not clearly evaluated.

Additional experiments suggested:

I think that the effect of EST2 downregulation is not entirely clear. The authors showed that

Est2 protein was undetectable by western blot, but telomeres from TI cells appear to be at least partially functional. However, the Southern blot in Fig. 1D lacks critical controls, such as est2Δ telomeres and type I and type II survivors, which are necessary for proper interpretation.
- Most of the analyses were performed after 200 PD, but it is unclear what happens before this point. Do the cells experience growth defects or a senescence-like phenotype soon after TI? How do telomeres behave before reaching equilibrium?
- Fig. 1C: at what time point were Est2 levels evaluated? Is there any change or increase in Est2 levels over time?
- Figs. 1C-F: est2p-17 and est2p-30 cells show undetectable Est2 protein levels, similar telomere structures, and accumulation of large cells, yet they form colonies differently on agar plates. What explains these differences? Have the authors analyzed the growth kinetics in these strains?
Finally, in the discussion, the authors claim that telomerase activity is present in TI cells, but they did not directly assess telomerase activity. This would require further clarification.
I think these experiments are realistic and could be done in few months.

Minor comments:

Specific experimental issues that are easily addressable:
- Fig. 3C: RAD9 deletion leads to a reduction in both cell cycle prolongation and aberrant mitosis in TI cells. What is its impact on cell viability? Viability can be assessed either by colony formation assays or by time-lapse experiments.
- How do other mutations that affect cell cycle arrest and mitotic defects in TI cells, such as the pds1 phosphorylation-defective allele or MEN mutations, impact cell viability?

Prior studies referenced appropriately, but some review are quite old (e.g. Melo and Toczkski, 2002; Cesare and Reddel, 2010).

The text and figures are generally clear and accurate. Suggestions to improve the clarity and the readability of the figures:

- Fig. 2B: Add estp-17 in the graph
- Legend strains of some graph are very small and difficult to read (e.g. Fig. 2B; 3A, C, D, E, ...)

Suggestions that would help the authors improve the presentation of their data and conclusions:

- Page 2: Briefly describe the concept of cohesion to help readers unfamiliar with the term.
- Page 4: Clarify the difference between telomerase insufficiency and telomerase deficiency.
- Page 4: Consider moving the description of telomere structure to the Introduction for better context.
- Page 5: Specify how many population doublings (PD) correspond to "10 passages on YEPD plates."
- Page 6: Present the description of the different phenotypes observed by microscopy first, and then describe the behavior of the different strains. This structure would make the text clearer for readers.
- Page 12: Define here what is meant by "collapsed spindle."
- Page 13: Consider quantifying the frequency of collapsed spindles during the different mitotic events.
- Discussion: The discussion is too long and difficult to follow. Consider condensing and reorganizing it to improve readability.

2. Significance:

Significance (Required)

This work advances our understanding of how cells respond to DNA damage. In particular, it provides insights into how individual cells within a population respond differently likely depending on the type and extent of damage they experience, thereby highlighting the heterogeneity of the DNA damage response. Such a response, on the one hand, coordinates cell cycle arrest with DNA repair, allowing cells sufficient time to remove the damage and subsequently progress through cell divisions with an intact genome. On the other hand, when this response persists, it promotes mitotic catastrophes and cell death, likely preventing the expansion of cells with genetic instability.

People working od DNA damage response will be interested in this work.

My expertise:

I work on the DNA damage response in yeast, with a specific focus on checkpoint signaling and the activation of checkpoint kinases. However, I do not have sufficient expertise to fully evaluate the contribution of MEN and cytokinesis, as I am not familiar with all the components and the complex interconnections between the pathways that regulate mitotic exit.

3. How much time do you estimate the authors will need to complete the suggested revisions:

Estimated time to Complete Revisions (Required)

(Decision Recommendation)

Between 3 and 6 months

4. *Review Commons* values the work of reviewers and encourages them to get credit for their work. Select 'Yes' below to register your reviewing activity at [Web of Science Reviewer Recognition Service] (formerly Publons); note that the content of your review will not be visible on Web of Science.

Web of Science Reviewer Recognition

No

Revision Plan

Manuscript number: RC-2025-03125
Corresponding author(s): Svetlana Makovets

[The "revision plan" should delineate the revisions that authors intend to carry out in response to the points raised by the referees. It also provides the authors with the opportunity to explain their view of the paper and of the referee reports.

The document is important for the editors of affiliate journals when they make a first decision on the transferred manuscript. It will also be useful to readers of the reprint and help them to obtain a balanced view of the paper.

If you wish to submit a full revision, please use our "Full Revision" template. ***It is important to use the appropriate template to clearly inform the editors of your intentions.****]*

1. General Statements [optional]

Dear Editor,

We are presenting our manuscript "*Extended DNA damage-induced G2/M arrests lead to aberrant mitoses and cell death due to excessive accumulation of securin*" by Anton Dubenko, Marah Jnied, Oleksii Kotenko, and Svetlana Makovets to be considered for publication as a research article.

In this study, we used time-lapse microscopy in combination with fluorescently tagged proteins to investigate cellular responses to nonfunctional telomeres and DNA breaks at the single-cell level. In each experiment, we analysed thousands of yeast cells undergoing mitoses in order to observe a full spectrum of different cellular behaviours, even quite rare ones. Unlike the population-level studies commonly used in the previous research on this topic, our approach allowed us to uncover these rare events, in particular, aberrant mitoses specific to the cells with prolonged G2/M arrests induced through DNA damage signalling. Furter studies focused on gaining molecular insights into the mechanisms of these aberrant mitoses led us to the discovery of Pds1 (securin) protein levels operating in each single cell like a stopwatch during the G2/M arrests (see the schematic at the end of the letter). DNA damage signalling starts the stopwatch by stabilising Pds1 through its phosphorylation by the checkpoint kinase Chk1 and the phosphorylated Pds1 progressively accumulates over the time of the arrest: the longer the arrest the higher the Pds1 levels are in an arrested cell, as we show at the single-cell level (Figure 6A, top). During mitosis, cells need to degrade Pds1 at the metaphase-to-anaphase transition in order to allow sister chromatid separation and nuclear division (Figure 6A, left part of the schematic). However, cells with prolonged arrests accumulate so much Pds1 that they struggle to degrade it in time and this leads to either a grossly delayed or a completely missed nuclear division (Figure 6A, right part of the schematic). As a result, cells undergo an

aberrant mitosis accompanied by missegregation of both their genome and spindle pole bodies, loss of rDNA and cell death in the following cell cycle. We manipulated the levels of Pds1 protein to demonstrate that lowering Pds1 partially suppresses aberrant mitoses in cells with DNA damage, but this comes at the cost of increased aneuploidy under the normal growth conditions. Therefore, the Pds1 protein levels are under a tight control to preserve the overall genome stability.

Although the aberrant mitoses were rarely observed during normal cell cycle progression, they occurred in about 30% of the cells undergoing a G2/M arrest and 50-80% of cells after extended arrests. This raises the possibility of this mechanism being selected during the evolution as a way to eliminate cells with extended G2/M, i.e the cells struggling to deal with DNA damage repair. In other words, this pathway might function as a quality control mechanism preserving genome stability in the populations of single-cellular organisms. Interestingly, such a mechanism is well-known in higher eukaryotes, where p53-p16 governed terminal arrests are triggered as part of a senescence program in cells not capable of a timely response to different stress signals, including DNA damage and nonfunctional telomeres. These cells are then eliminated by macrophages in order to preserve the genome stability and suppress carcinogenesis. It has been discovered recently that stabilisation of p53 during prolonged mitoses acts as a stopwatch in human cells and its accumulation leads to terminal G1 arrests following abnormally long mitoses (Meitinger et al., 2024; DOI: 10.1126/science.add9528). Therefore, the Pds1-dependent mechanism we describe here functionally parallels the p53-dependent stopwatch in higher eukaryotes and operates in a similar manner, through accumulation of a stabilised protein.

The manuscript has been reviewed by 3 reviewers and we find most of the comments constructive and helpful for further improving the presented data and their presentation. We submit a list of additional experiments and data analyses we will perform according to Reviewers' requests. We think that not having an expert on telomeres was to our disadvantage as only Reviewer 1 fully appreciated the newly described yeast model on telomerase insufficiency as well as the overall significance of our findings on population dynamics in cells with compromised telomeres and DNA damage. We believe that our findings are of interest to a rather broad readership in the fields of genome stability, telomere biology, as well as microbial population dynamics and evolution.

2. Description of the planned revisions

Here is a summary of all the planned revisions, with the references to particular reviewer's comments. The point-by-point responses to all the reviewers' comments are included as a separate file.

1. Optimize the Est2 western blotting to detect Est2 in the cells with telomerase insufficiency (TI) to estimate the protein level reduction

factor relative to the WT Est2 levels (in response to Reviewer 2, comment 1; R3, comm 1 and 5);
2. Add Southern blots for telomere analysis during TI cell passaging and type I and type II survivors (R3, comm 1, 2, and 5);
3. Analyze mitotic events in cells with single unrepairable DSBs (R1, comm1);
4. Compare the duration of arrests in Class 3.1 vs Class 3.2 mitotic evens (R1, comm 1)
5. Quantify mitotic events shown in Fig 5E-F (R1, comm 2, R2, comm 5 and R3, in the presentation comments);
6. Extend the analysis of Securin accumulation (Fig 4D) to more cells, more mitotic division types and further time-points in the cells cycle to show Pds1 protein level decrease before the cell divisions (R1, comm 3 and R2, comm 4);
7. Use TEV-cleavable cohesin to address the reasons for aberrant mitosis (R1, comm 4);
8. Cell viability analysis in *rad9*, *pds1-m9* and MEN pathway mutants (R3, comm 6 and 7);
9. We will also do our best to address all the comments on improving the presentation of the manuscript.

3. Description of the revisions that have already been incorporated in the transferred manuscript

Please insert a point-by-point reply describing the revisions that were already carried out and included in the transferred manuscript. If no revisions have been carried out yet, please leave this section empty.

4. Description of analyses that authors prefer not to carry out

Please include a point-by-point response explaining why some of the requested data or additional analyses might not be necessary or cannot be provided within the scope of a revision. This can be due to time or resource limitations or in case of disagreement about the necessity of such additional data given the scope of the study. Please leave empty if not applicable.

1. Suppression of G2/M arrest by *rad9* deletion and monitoring Rad53 phosphorylation in cells with DSBs (R2, comm 3). This has been extensively characterized in the literature. In addition, the focus of the manuscript is more of a downstream, on Pds1 which we analysed both in cells with TI and DSBs, in order to draw a strong parallel between the responses to TI and DSBs (Figure 4).

2. Accumulation of Pds1 by qRT-PCR in cells with DNA damage signalling (Rev 2, comm 4) – we believe that this approach is not appropriate for the analysis of rare events in live single cells. For obvious reason, the qRT-PCR can't be performed on the single cells which are being imaged by live microscopy. At the populational level, qRT-PCR won't be informative as the majority of the cells are not relevant to aberrant mitoses. Furthermore, the Pds1 protein levels on similar populations with DNA damage signalling have been reported previously by the Elledge lab (Wang et al., 2001).

Point-by-point responses to reviewers' comments (the responses are in Italic):

Reviewer #1 (Evidence, reproducibility and clarity (Required)):

In their paper, Dubenko et al. investigated how cells respond to DNA double-strand breaks and telomere dysfunction at the single-cell level using time-lapse microscopy in budding yeast. To model telomerase insufficiency (TI), they weakened the EST2 promoter, mimicking partial telomerase downregulation. This led to shortened but stably maintained telomeres and DNA damage responses. They also analyzed est2Δ mutants and phleomycin-treated cells to corroborate their findings. DNA damage caused highly variable G2/M arrest durations, with prolonged arrests frequently leading to aberrant mitoses characterized by mis-coordination of nuclear division and cytokinesis, nucleolar loss, and eventual cell death. The underlying mechanism involved Chk1-dependent phosphorylation of securin (Pds1), whose progressive accumulation delayed or blocked nuclear division. Reducing PDS1 expression partially suppressed aberrant mitoses after prolonged G2/M arrest, but at the cost of increased aneuploidy under normal growth conditions. Similarly, deletion of LTE1, which delays mitotic exit and cytokinesis, reduced aberrant mitoses by allowing more time for Pds1 degradation. The authors conclude that progressive Pds1 accumulation drives aberrant mitoses, thereby eliminating cells with prolonged damage responses - a potential quality control mechanism that preserves genomic stability in yeast. They further suggest that this may parallel the p53-53BP1-USP28 mitotic stopwatch in human cells, which promotes p53 stabilization to eliminate genomically compromised cells.

Major comments

1. Aberrant mitoses occur after DNA damage mediated cell cycle arrest. Following points would help to understand the nature these aberrant mitoses: Do the aberrant mitoses occur after DNA repair or instead arise from slippage out of arrest? Is there a correlation between the duration of DNA damage mediated cell cycle arrest and the different classes of aberrant mitoses? For example, do longer arrests result in class 3.1 events, while relatively shorter arrests lead to class 3.2 events?

> *We will address the first question by analysing cells with a single unrepairable HO-induced break. If they progress into aberrant mitoses then arrest slippage is possible. We will also analyse the duration of arrests prior to Class 3.1 and 3.2 events using the existing datasets to see if there is a correlation proposed by the Reviewer.*

2. The data shown in Figure 5 need quantification. What are the frequencies of the events shown? What is the timing between actomyosin ring contraction and

chromosome segregation? When chromosomes segregate after prolonged arrest, do the separated nuclei always appear in the bud?

> *We will try to quantify the events in Fig 5D-F as suggested, though the numbers for the relatively rare Class 3.1 events might be too low for any statistically significant conclusion.*
>
> *When chromosomes segregate after a prolonged arrest, sometimes they segregate normally (Class 1 events) and other times in the bud (Class 3.1). It is a probabilistic process as can be seen from the distributions of the mitotic evens in Fig 3A-B.*

3. The authors show that Securin accumulates during prolonged arrest. Securin accumulation suggests Separase inactivation and thus failure of Cohesin cleavage. However, some cells still separate their DNA, suggesting that Cohesin is cleaved in at least a fraction of cases. What percentage of cells experiencing G2/M arrest show Securin accumulation? Do all cells that undergo aberrant mitosis have high levels of Securin? Is this the predominant phenotype? Do cells that separate their DNA after a long DNA damage dependent cell cycle arrest actually degrade Securin or somehow escape the Securin barrier manage to separate their DNA?

> *We will extend the analysis of Securin accumulation to address these questions, by including more cells into the analysis and also showing how the Securin level drops at the chromosome segregation window. We observed this but have not included in the manuscript.*

4. The authors state that short spindles collapse during class 3.2 mitotic events. What is the cell cycle stage here; pre- or post-anaphase? Are sister chromatids separated? In other words, does the failure of nuclear division stem from defective spindle elongation or from failure of cohesin cleavage? Since Esp1 (separase) is also a midzone protein involved in spindle midzone assembly,and is also critical for CdcFourteen-Eary-Anaphase Release (FEAR) it would be important to clarify this point.

> *We will address this question by using a system with cohesin cleavage induced by the expression of TEV-protease. If the aberrant mitoses stem from a defect in cohesin cleavage, then cleaving it externally by TEV should suppress the aberrant mitoses, at least partially.*

Minor comments

5. The link between the prolonged DNA damage arrest and aberrant mitoses here seems to be the Pds1 phosphorylation by Chk1, and consequent Pds1 stabilization. However, I feel that this finding is diluted in the results section. A change in the data presentation order may help bringing the result forward.

We will stress this point more in the text.

6. The text and main figure (Figure 2) explaining the classes of aberrant mitoses are difficult to follow. Representative time-lapse image series and graphs from Figure S1 are more complete and easier to interpret, and could be moved into the main figure instead.

We will consider re-arranging the figures as suggested.

7. Dark orange bars in Figures 3A and 3F are not indicated in the legend. These type of graphs are also difficult to read due to overlapping data series. It would be better to represent the data in a different way- ie. By showing individual data series separately.

The dark orange colour is the overlap between the half transparent yellow (Class 1) and red (Class 3) datasets. We will think on how to make data presentation clearer to the reader.

8. The data presented in Figure 3B are not clearly explained.

We will modify the explanation.

9. Striped or dotted graph fill patterns should be avoided, as they are barely visible, especially in legends.

We will address this.

10. The discussion could be more concise.

We will try to shorten it.

11. One-way ANOVA should be used when comparing more than two samples.

We will look into the stats again.

12. Nuclear divisions occurring within one cell compartment (Figure 5) resemble failures

of the spindle position checkpoint (SPOC), which normally inhibits mitotic exit and cytokinesis when spindle elongation into the bud fails. This checkpoint arrests cells in M phase. Interestingly, the most downstream SPOC component (Bfa1) is also required for cell cycle arrest in response to telomere dysfunction. This raises the question of whether Bfa1 is required to arrest these cells with compromised telomeres in G2/M or M phase. Could the authors elaborate on this?

> *This is a valid point. Based on the data, it does look like Bfa1 is required to arrest cells with compromised telomeres, maybe not as critically as Rad9, but it certainly plays a role. We will bring this up in the Discussion.*

Reviewer #1 (Significance (Required)):

This study will be of interest to researchers working on DNA damage response, cell cycle regulation, telomere biology, and genome stability. It may also engage broader audiences in aging, cancer biology. The yeast telomerase insufficiency (TI) model developed here is a valuable tool for mimicking mammalian cells in which expression of the telomerase catalytic subunit TERT is downregulated during development. Conceptually, the study highlights parallels between yeast and mammalian systems, suggesting evolutionary conservation of quality-control mechanisms that eliminate compromised cells.

Reviewer expertise: Cell cycle control, checkpoints.

Reviewer #2 (Evidence, reproducibility and clarity (Required)):

In this manuscript, the authors analyse the effects of a decrease in telomerase in budding yeast S. cerevisiae on G2/M cell arrest. Using different immunofluorecent tools and time lapse microscopy together with different genetic approaches, they show that telomere insufficiency correlates with cytokinesis problems together with accumulation Pds1 resulting in increased aneuploidy. They conclude that longer arrests may eliminate genetically unstable cells.
The consequences of telomerase deficiency have been extensively studied (work by the labs of Teixeira, Lingner, Shore, Zakian, Wellinger, etc). At the single-cell it has been already shown that senescence varies considerably from one cell to another, that cells can switch from rapid proliferation to a prolonged cell cycle in a single division, and that long cell cycles precede cell death (Xu et al 2014, Coutelier, et al 2018).

This work aims to explore the results of a decrease in telomerase expression and to study the mechanisms responsible for cycle defects. In this sense, this work deserves publication, but at this stage, the manuscript needs to be improved at many levels to make it appropriate for publication despite the impressive large number of experimental data. The article is quite dense and could be made easier to the reader by clearly highlighting the novelty brought to the field. A way to consider could be to focus on some aspects of the different data, or even considering splitting the data in two publications?
Below are a few points to consider strengthening the impact of this work.

1. The authors use an unusual method in yeast where conditional promoters (GAL, tetO2, CUP1) are well controlled. As a result, the position of the Hygromycin cassette in the EST2 promoter must be improved in the text and figure. Above all, expression levels are not visible on the Western blot, must be quantified by RT-qPCR, and must be shown for all constructs. (both expression and growth). In Fig. 1C, it appears that the actin control comes from a different gel, which should be avoided. In Fig.1F how is cell size calculated?

> *We will address this by optimising Est2 western blotting. We believe that we could make the Est2 signals in the cells with TI detectable.*

2. The estimated probabilities of class 3 events assume that a class 2 event becomes either a class 1 event or a class 3 event, but do not consider the fact that class 2 events can also lead to cell death. How do the authors account for this bias? Can the authors explain why they chose to perform 5 Z-stacks of 0.6 µm without projection? Z-projection imaging might be more accurate for the analyses.

> *Based on our observations, the cell death events are too rare relatively to either Class 1 or Class 3 events and for this reason they could be ignored as a possible outcome to simplify the analysis. However, we will look into quantifying the death events and either explain the justification of their exclusion from the analysis or take them into account.*
>
> *As for the microscopy analysis, we found that moving across different imaging planes for every event instead of using Z-projection gave a clearer picture of the events inside the cells. This approach was used for analysing nuclear morphology, not to quantify a fluorescent signal.*

3. The conclusion of the experiments in Fig. 3 is that DNA damage and decreased expression of est2 (if well documented, see comment 1) are comparable. Suppression by

Δ rad9 must be shown in both cases. To confirm that G2/M arrest is due to DNA damage, analysis of H2A phosphorylation or Rad53 phosphorylation would be beneficial. The conditions for phleomycin treatment must be specified (time). The graphs in Fig. 3A, 3B and 3F could be made easier to understand. What are the percentages calculated on?

The Rad9 involvement (as well as Rad53 phosphorylation) in the cell cycle arrest in response to both DNA breaks and compromised telomeres (cells with telomerase deficiency) has been extensively characterised in the literature. The key downstream component of the DNA damage signalling pathway analysed here is Pds1 (Securin) which we assayed for both TI and DNA breaks (Phleo), as shown in Figure 4. Given the focus of the manuscript on aberrant mitoses and not as much on the upstream signalling leading to Pds1 phosphorylation, we believe that the pds1-m9 analysis is both more informative, than rad9 and rad53 would be, and sufficient to draw a parallel between TI and DSBs.

4. The conclusions concerning Pds1 should be interpreted with caution, as it has not been proven that aberrant mitoses are caused by the accumulation of Pads1; only a correlation has been demonstrated. We propose to add control strains (WT, Δest2, WT treated with phleomycin). The measurement should be performed on more than 15 cells, and the method should be explained in the Materials and Methods section. Finally, accumulation should be quantified by RT-qPCR to consolidate the results.

We think the Reviewer might have missed the Figure 4E-F data establishing the cause-effect relationship between the expression levels of Pds1 and aberrant mitoses. We show that reducing the PDS1 expression partially suppresses aberrant mitoses.

The analysis of Pds1 levels at the single cell level will be extended to more cells. We don't think that the proposed RT-qPCR for PDS1 expression is appropriate here. It can't be performed at the single cell level and won't be informative at the population level. In addition, the Pds1 protein dynamics in cells with DNA damage have been analysed at the population level before (Wang et al, 2001).

5. The loss of coordination between nuclear division and cytokines is interesting but needs to be supported by quantification and controls (WT, Δest2, MEN mutants).

We will try to quantify the events in the cells with TI. The quantifications in the WT cells would be close to technically impossible to do as the Class 3 events are extremely rare.

Minor comments
The authors should make an effort to cite recent work in the field and position their current analysis accordingly, and make sure references are not missing (for instance type I and type II survivors). It would be helpful to also improve the concision and the clarity of the results section, with concluding sentences for each result.
Work could be done on the figures to simplify them. In particular, the bar charts are quite complicated to understand given all the mutations that have been made and the fact that they are each subdivided into classes 1, 2, and 3.

We will address these suggestions in the manuscript text.

Reviewer #2 (Significance (Required)):

This work aims to explore the results of a decrease in telomerase expression and to study the mechanisms responsible for cycle defects. In this sense, this work deserves publication, but at this stage, the manuscript needs to be improved at many levels to make it appropriate for publication despite the impressive large number of experimental data. The article is quite dense and could be made easier to the reader by clearly highlighting the novelty brought to the field. A way to consider could be to focus on some aspects of the different data, or even considering splitting the data in two publications?

Specialized audience.

Our expertise in DNA repair and yeast genome organization allows us to offer what we hope are constructive comments on this work, even though not telomere specialists.

Reviewer #3 (Evidence, reproducibility and clarity (Required)):

In this manuscript, Dubenko and colleagues investigated, at a single-cell level, how yeast cells regulate cell cycle arrest and recovery in response to DNA breaks or telomere dysfunctions, as well as the potential outcomes for cells that resume cell cycle progression after arrests of varying durations. They used Saccharomyces cerevisiae populations in which telomere defects were induced by downregulating telomerase

expression, while DNA breaks were generated through phleomycin treatment. Time-lapse microscopy was employed to measure the duration of checkpoint-induced cell cycle blocks, nuclear division, and the localization of key proteins and structures in individual cells, with the goal of exploring heterogeneity in the DNA damage response and the relationship between cell cycle arrest and chromosome stability.

To monitor nuclear dynamics, the authors generated yeast strains with reduced telomerase expression carrying GFP-tagged histones and mCherry-labeled Nop56 for time-lapse imaging. Both telomerase downregulation and phleomycin treatment led to an increased frequency of aberrant DNA segregations following G2/M cell cycle arrests. This frequency was higher in cells experiencing prolonged arrests. Elevated levels of Pds1 were observed in cells with extended checkpoint activation, whereas partial loss of Pds1 function reduced the frequency of abnormal segregations but increased the incidence of aneuploidy.

Analysis of mitotic spindle dynamics through time-lapse microscopy indicated that the spindle becomes unstable in cells undergoing prolonged cell cycle arrest. Furthermore, mutations that delayed cytokinesis decreased the occurrence of aberrant mitoses, while mutations promoting premature activation of MEN had the opposite effect, increasing aberrant mitoses.

Overall, the authors concluded that sustained activation of the DNA damage checkpoint leads to mitotic defects, aneuploidy, and cell death over a few divisions in cells that take too long to repair DNA damage. They proposed that this mechanism could eliminate cells with unstable genomes.

Major comments:

The data and the methods presented in such a way that they can be reproduced.

The experiments are adequately replicated and statistical analysis adequate.

Most of the key conclusions are convincing, particularly those concerning the relationship between prolonged cell cycle arrest and mitotic defects, the role of Pds1, and the coordination between chromosome segregation and mitotic exit/cytokinesis.

However, while the authors propose that prolonged arrests and mitotic defects may occur in cells that take too long to repair DNA damage, they did not directly assess DNA damage persistence.

Another important point that, in my opinion, need further investigation is the impact of EST2 downregulation on cell growth kinetics and telomere integrity.

The extent of telomerase downregulation, as well as the persistence of telomerase activity in TI strains, was not clearly evaluated.

Additional experiments suggested:

1. I think that the effect of EST2 downregulation is not entirely clear. The authors showed that Est2 protein was undetectable by western blot, but telomeres from TI cells appear to be at least partially functional. However, the Southern blot in Fig. 1D lacks critical controls, such as est2Δ telomeres and type I and type II survivors, which are necessary for proper interpretation.

> *We will add Est2 western with detectable telomerase and Southerns with the type I and II survivors.*

2. -Most of the analyses were performed after 200 PD, but it is unclear what happens before this point. Do the cells experience growth defects or a senescence-like phenotype soon after TI? How do telomeres behave before reaching equilibrium?

> *The telomeres shorten in the first few passages, as expected, and then equilibrate as short and stable. We will add a Southern blot of telomere analysis at each passage.*

3. -Fig. 1C: at what time point were Est2 levels evaluated? Is there any change or increase in Est2 levels over time?

> *The levels were evaluated right after reducing the telomerase expression levels in order to avoid the complications caused by the changes in response to compromised telomeres – the cell cycle profile shifting to G2/M and the cell size increases, which are both likely to affect the ratio of nuclear proteins to actin in general. By avoiding this, one could compare the levels of Est2 protein in cells with TI to the WT levels to estimate the reduction factor.*

4. -Figs. 1C-F: est2p-17 and est2p-30 cells show undetectable Est2 protein levels, similar telomere structures, and accumulation of large cells, yet they form colonies differently on agar plates. What explains these differences? Have the authors analyzed the growth kinetics in these strains?

> *The growth kinetics is shown in Fig 1E-G, not sure what else we could add here. As for the explanation in the colony size, the est2p-17 have a larger fraction of cells experiencing G2/M arrest (see Fig S1B, est2p-17 have a smaller fraction of cells with Normal Divisions than est2p-30 cells) and also longer duration of the arrests on average (Fig S1B, est2p-17 have a larger fraction of Class 2 cells than est2p-30 cells, which is an indirect representation of the duration of arrests: the longer the arrests on average, the larger fraction of cells will be scored as arrested at the end*

of the video). These two factors result in the longer population doubling time (see Figure 1G) and therefore a slower growth on agar plates (Fig 1E).

5. Finally, in the discussion, the authors claim that telomerase activity is present in TI cells, but they did not directly assess telomerase activity. This would require further clarification.

We are hoping to add a western blot with detectable Est2 in cells with TI. The telomerase activity is clearly present in the cells with TI because the telomeres maintain the short and stable structure (Fig 1D). This is very different from the cells completely lacking telomerase activity where the switching to recombination-dependent telomere maintenance leads to very clear changes in telomere structure. This will be clearer once we add samples for telomerase-deficient survivors to the Southern blots.

I think these experiments are realistic and could be done in few months.

Minor comments:
Specific experimental issues that are easily addressable:
6. -Fig. 3C: RAD9 deletion leads to a reduction in both cell cycle prolongation and aberrant mitosis in TI cells. What is its impact on cell viability? Viability can be assessed either by colony formation assays or by time-lapse experiments.

We will do these experiments and add the data.

7. - How do other mutations that affect cell cycle arrest and mitotic defects in TI cells, such as the pds1 phosphorylation-defective allele or MEN mutations, impact cell viability?

We will do these experiments and add the data.

Prior studies referenced appropriately, but some review are quite old (e.g. Melo and Toczkski, 2002; Cesare and Reddel, 2010).

The text and figures are generally clear and accurate. Suggestions to improve the clarity and the readability of the figures:

We will address all the suggestions below on the presentation of the manuscript.

-Fig. 2B: Add estp-17 in the graph

-Legend strains of some graph are very small and difficult to read (e.g. Fig. 2B; 3A, C, D, E, ...)

- Suggestions that would help the authors improve the presentation of their data and conclusions:

-Page 2: Briefly describe the concept of cohesion to help readers unfamiliar with the term.

-Page 4: Clarify the difference between telomerase insufficiency and telomerase deficiency.

-Page 4: Consider moving the description of telomere structure to the Introduction for better context.

-Page 5: Specify how many population doublings (PD) correspond to "10 passages on YEPD plates."

-Page 6: Present the description of the different phenotypes observed by microscopy first, and then describe the behavior of the different strains. This structure would make the text clearer for readers.

-Page 12: Define here what is meant by "collapsed spindle."

-Page 13: Consider quantifying the frequency of collapsed spindles during the different mitotic events.

-Discussion: The discussion is too long and difficult to follow. Consider condensing and reorganizing it to improve readability.

Reviewer #3 (Significance (Required)):

This work advances our understanding of how cells respond to DNA damage. In particular, it provides insights into how individual cells within a population respond differently likely depending on the type and extent of damage they experience, thereby highlighting the heterogeneity of the DNA damage response. Such a response, on the one hand, coordinates cell cycle arrest with DNA repair, allowing cells sufficient time to remove the damage and subsequently progress through cell divisions with an intact genome. On the other hand, when this response persists, it promotes mitotic catastrophes and cell death, likely preventing the expansion of cells with genetic instability.

People working od DNA damage response will be interested in this work.

My expertise:
I work on the DNA damage response in yeast, with a specific focus on checkpoint signaling and the activation of checkpoint kinases. However, I do not have sufficient expertise to fully evaluate the contribution of MEN and cytokinesis, as I am not familiar with all the components and the complex interconnections between the pathways that regulate mitotic exit.

December 4, 2025

Re: JCB manuscript #202511106T

Svetlana Makovets
University of Edinburgh

Dear Dr. Makovets,

Thank you for submitting your manuscript entitled "Extended DNA damage-induced G2/M arrests lead to aberrant mitoses and cell death due to excessive accumulation of securin". We have assessed your revision plan and would be happy to send a suitably revised paper back to the original referees for re-review.

While you are revising your manuscript, please also attend to the following editorial points to help expedite the publication of your manuscript. Please direct any editorial questions to the journal office.

GENERAL GUIDELINES:

Text limits: Character count for an Transfer is < 40,000, not including spaces. Count includes title page, abstract, introduction, results, discussion, and acknowledgments. Count does not include materials and methods, figure legends, references, tables, or supplemental legends.

Figures: Transfers may have up to 10 main text figures. Figures must be prepared according to the policies outlined in our Instructions to Authors, under Data Presentation, https://jcb.rupress.org/site/misc/ifora.xhtml. All figures in accepted manuscripts will be screened prior to publication.

IMPORTANT: It is JCB policy that if requested, original data images must be made available. Failure to provide original images upon request will result in unavoidable delays in publication. Please ensure that you have access to all original microscopy and blot data images before submitting your revision.

Supplemental information: There are strict limits on the allowable amount of supplemental data. Transfers may have up to 5 supplemental figures. Up to 10 supplemental videos or flash animations are allowed. A summary of all supplemental material should appear at the end of the Materials and methods section.

Please note that JCB now requires authors to submit Source Data used to generate figures containing gels and Western blots with all revised manuscripts. This Source Data consists of fully uncropped and unprocessed images for each gel/blot displayed in the main and supplemental figures. For assays performed using capillary electrophoresis and/or immunoassay-based detection, authors should instead provide the electropherogram graph(s) for each experiment, plotting fluorescence/chemiluminescence intensity vs. molecular weight/size. Please be sure to provide one Source Data file for each figure gels, blots, and/or capillary electrophoresis assays along with your revised manuscript files. File names for Source Data figures should be alphanumeric without any spaces or special characters (i.e., SourceDataF#, where F# refers to the associated main figure number or SourceDataFS# for those associated with Supplementary figures). For traditional gels and blots, the lanes of the gels/blots should be labeled as they are in the associated figure, the place where cropping was applied should be marked (with a box), and molecular weight/size standards should be labeled wherever possible. For capillary electrophoresis assays, each trace in the graph should be color-coded and labeled to indicate which protein, gene, or sample is being measured (please try to avoid red/green combinations to accommodate our color-blind readers).

Source Data files will be made available to reviewers during evaluation of revised manuscripts and, if your paper is eventually published in JCB, the files will be directly linked to specific figures in the published article.

Source Data Figures should be provided as individual PDF files (one file per figure). Authors should endeavor to retain a minimum resolution of 300 dpi or pixels per inch. Please review our instructions for export from Photoshop, Illustrator, and PowerPoint here: https://rupress.org/jcb/pages/submission-guidelines#revised

The typical timeframe for revisions is three to four months. If you anticipate any difficulties in meeting this aforementioned revision time limit, please contact us and we can work with you to find an appropriate time frame for resubmission. Please note that papers are generally considered through only one revision cycle, so any revised manuscript will likely be either accepted or rejected.

When submitting the revision, please include a cover letter addressing the reviewers' comments point by point. Please also highlight all changes in the text of the manuscript.

We hope that the comments below will prove constructive as your work progresses. We would be happy to discuss them further once you've had a chance to consider the points raised in this letter.

Thank you for this interesting contribution to Journal of Cell Biology. You can contact us at the journal office with any questions at cellbio@rockefeller.edu.

Sincerely,

Daniel Lew
Monitoring Editor
Journal of Cell Biology

Therese Gerbich
Scientific Editor
Journal of Cell Biology

Response to Reviewers' Comments

We would like to thank all the reviewers for constructive criticism and very helpful suggestions. We tried to address as many concerns as we could and believe that the revised version of the manuscript has been significantly improved following all the received advice.

The major changes to the manuscript include:

1. *The figures have been re-arranged to include 10 main and 5 supplemental, by adding 2 of the supplemental figures to the main ones and splitting one of the main figures into two. Therefore, the figure numbering has changed since the original submission.*
2. *In response to the Reviewer 2 comments, we abandoned the idea of making assumptions on how Class 2 events might be resolved by either Class 1 or Class 3 mitoses, mainly because cell death should be considered as a third valid outcome, as pointed out the Reviewer. Therefore, anticipating the outcomes of the Class 2 events seems more complex than we originally considered. Accordingly, the corresponding graphs based on splitting the Class 2 events into Class 1 and Class 3 have been taken out. Two of them (Figure 2D and 5D) have been replaced with different graphs, where the probability of aberrant mitoses following an arrest is calculated relative to all the arrested cells observed, with the explanation in the text that these numbers would be underestimating Class 3 events: some of the Class 2 cells are to resolve through Class 3 as well.*
3. *Where appropriate, the P-values have been recalculated based on one-way ANOVA as suggested by Reviewer 1.*
4. *In response to Reviewr 3 comments, a dataset analysing mitoses in response to single unrepairable DSBs (introduced by HO nuclease) either in the middle of a chromosome or at a subtelomere has been added to demonstrate that aberrant mitoses can occur when cells divide with unrepaired DNA damage (Figure 5G).*
5. *The analysis of Pds1 accumulation in cells with TI was completely re-done, now with the focus on a comparison of the time it takes to degrade accumulated Pds1 prior to Class 1 and Class 3 mitoses (Figure 6C-D). We demonstrate that, indeed, aberrant mitoses correlate with the longer time periods it takes cells to get rid of Pds1 prior to cell division. This should address the comments on different aspects of Pds1 accumulation by all 3 Reviewers.*
6. *In response to Reviewer 1 comments, we tested our earlier hypothesis that Class 3.1 cells might have shorter G2/M arrests than Class 3.2 cells but we couldn't detect such a difference (Figure 7E). The text has been modified to remove the speculations around the hypothesis.*
7. *The effect of different checkpoint mutations on cell death in cells with TI has been analysed and presented in Figure 9C.*
8. *In addition to the listed above changes, the text of the manuscript has been edited to incorporate most of the suggestions by the reviewers and also to match the modifications to the figures. In particular, the Discussion has been shortened significantly as asked by the reviewers. The paper title had to be shortened due to the character limits by JCB.*

There were also two experiments which we attempted in response to the reviewers' comments, but they didn't work quite well enough. We included the results in our point-by-point responses but not in the manuscript.

Below are point-by point responses to the reviewers' comments (the responses are in Italic).

Reviewer #1 (Evidence, reproducibility and clarity (Required)):

In their paper, Dubenko et al. investigated how cells respond to DNA double-strand breaks and telomere dysfunction at the single-cell level using time-lapse microscopy in budding yeast. To model telomerase insufficiency (TI), they weakened the EST2 promoter, mimicking partial telomerase downregulation. This led to shortened but stably maintained telomeres and DNA damage responses. They also analyzed est2Δ mutants and phleomycin-treated cells to corroborate their findings. DNA damage caused highly variable G2/M arrest durations, with prolonged arrests frequently leading to aberrant mitoses characterized by mis-coordination of nuclear division and cytokinesis, nucleolar loss, and eventual cell death. The underlying mechanism involved Chk1-dependent phosphorylation of securin (Pds1), whose progressive accumulation delayed or blocked nuclear division. Reducing PDS1 expression partially suppressed aberrant mitoses after prolonged G2/M arrest, but at the cost of increased aneuploidy under normal growth conditions. Similarly, deletion of LTE1, which delays mitotic exit and cytokinesis, reduced aberrant mitoses by allowing more time for Pds1 degradation. The authors conclude that progressive Pds1 accumulation drives aberrant mitoses, thereby eliminating cells with prolonged damage responses - a potential quality control mechanism that preserves genomic stability in yeast. They further suggest that this may parallel the p53-53BP1-USP28 mitotic stopwatch in human cells, which promotes p53 stabilization to eliminate genomically compromised cells.

Major comments

1. Aberrant mitoses occur after DNA damage mediated cell cycle arrest. Following points would help to understand the nature these aberrant mitoses: Do the aberrant mitoses occur after DNA repair or instead arise from slippage out of arrest? Is there a correlation between the duration of DNA damage mediated cell cycle arrest and the different classes of aberrant mitoses? For example, do longer arrests result in class 3.1 events, while relatively shorter arrests lead to class 3.2 events?

> *We believe at least some of the aberrant mitosis arise from arrest slippages. We added an experiment where we analysed cell divisions following a single unrepairable DSB (HO-inducible) and saw an increase in aberrant mitoses in this*

setting (Fig 5G, Class 3 increase in cells with a DSB at HEM13). Because the break can't be repaired and its induction was confirmed in each scored cell by observing Rad52 foci, then the divisions must have resulted from arrest slippages.

We also analysed the duration of arrests prior to Class 3.1 and 3.2 events using the existing datasets and could not detect any difference (Fig 7E). The text has been modified accordingly, i.e. the speculations on the possible difference have been removed.

2. The data shown in Figure 5 need quantification. What are the frequencies of the events shown? What is the timing between actomyosin ring contraction and chromosome segregation? When chromosomes segregate after prolonged arrest, do the separated nuclei always appear in the bud?

We quantified the events as suggested and added the numbers of analysed events in the figure legend (Fig 7 in the revised version). Please note that the numbers for the relatively rare Class 3.1 events are much lower than for less rare Class 3.2.

When chromosomes segregate after a prolonged arrest, sometimes they segregate normally (Class 1 events) and other times in the bud (Class 3.1). In Class 1, half of the chromatin is pulled from the neck area back to the mother cell. This movement seem obstructed by a prematurely contracting myosin ring in Class 3.1.

3. The authors show that Securin accumulates during prolonged arrest. Securin accumulation suggests Separase inactivation and thus failure of Cohesin cleavage. However, some cells still separate their DNA, suggesting that Cohesin is cleaved in at least a fraction of cases. What percentage of cells experiencing G2/M arrest show Securin accumulation? Do all cells that undergo aberrant mitosis have high levels of Securin? Is this the predominant phenotype? Do cells that separate their DNA after a long DNA damage dependent cell cycle arrest actually degrade Securin or somehow escape the Securin barrier manage to separate their DNA?

We significantly extended the analysis of Securin accumulation to address these questions, by 1). Comparing Class 1 and Class 3 events instead of monitoring Class 2; 2). including more cells into the analysis and 3). not only showing how the Securin accumulates, but mainly focusing on how its level drops at the chromosome segregation window (Figure 6C-D in the revised version, with more details added in the legend). We could observe that aberrant mitoses correlate with longer time of Securin degradation. Cells always degrade securin before they divide.

4. The authors state that short spindles collapse during class 3.2 mitotic events. What is the cell cycle stage here; pre- or post-anaphase? Are sister chromatids separated? In other words, does the failure of nuclear division stem from defective spindle elongation or from failure of cohesin cleavage? Since Esp1 (separase) is also a midzone protein involved in spindle midzone assembly,and is also critical for CdcFourteen-Eary-Anaphase Release (FEAR) it would be important to clarify this point.

We tried to address this question by using a system with cohesin cleavage induced by the expression of TEV-protease in cells treated with phelomycin to trigger the DNA damage. If the aberrant mitoses stem from a defect in cohesin cleavage, then cleaving it externally by TEV should suppress the aberrant mitoses, at least partially. However, the results were not straight forward to interpret because

1) *adding galactose to raffinose-grown cells treated with phleomycin resulted in an increase in aberrant mitoses for some unknown reason, see Figure N1 below where 4 biological repeats are plotted separately (solid colour - RAF, stripes represent GAL addition). In all 3 control genotypes (grey, yellow, blue), where the addition of galactose didn't induce TEV protease-dependent cleavage of cohesin, the galactose induction caused a further increase in aberrant mitoses (stripy bars are higher than solid ones).*
2) *SCC1-TEV must have leaky expression in Raffinose (solid blue bars) as the presence of this construct by itself significantly increased aberrant mitoses (compare solid blue to solid grey).*

Despite these complications, the presence of the full system sufficient to cleave cohesin through gal-inducible TEV expression resulted in a decrease in aberrant mitoses (green). The trend was the same in all 4 repeats but the absolute numbers differed and this unexpected effects of galactose and SCC1-TEV in control strains made it very difficult to analyse – we couldn't detect a statistically-significant difference if we just compare RAF to GAL in the pGAL-TEV SCC1-TEV strains (Figure N2). We decided not to include the data but would be grateful for any advice from the Reviewer on the possibilities of including them in some state. Alternatively, these results could stay in the Responses to the Reviewers which we assume will be publicly available via BioRxiv.

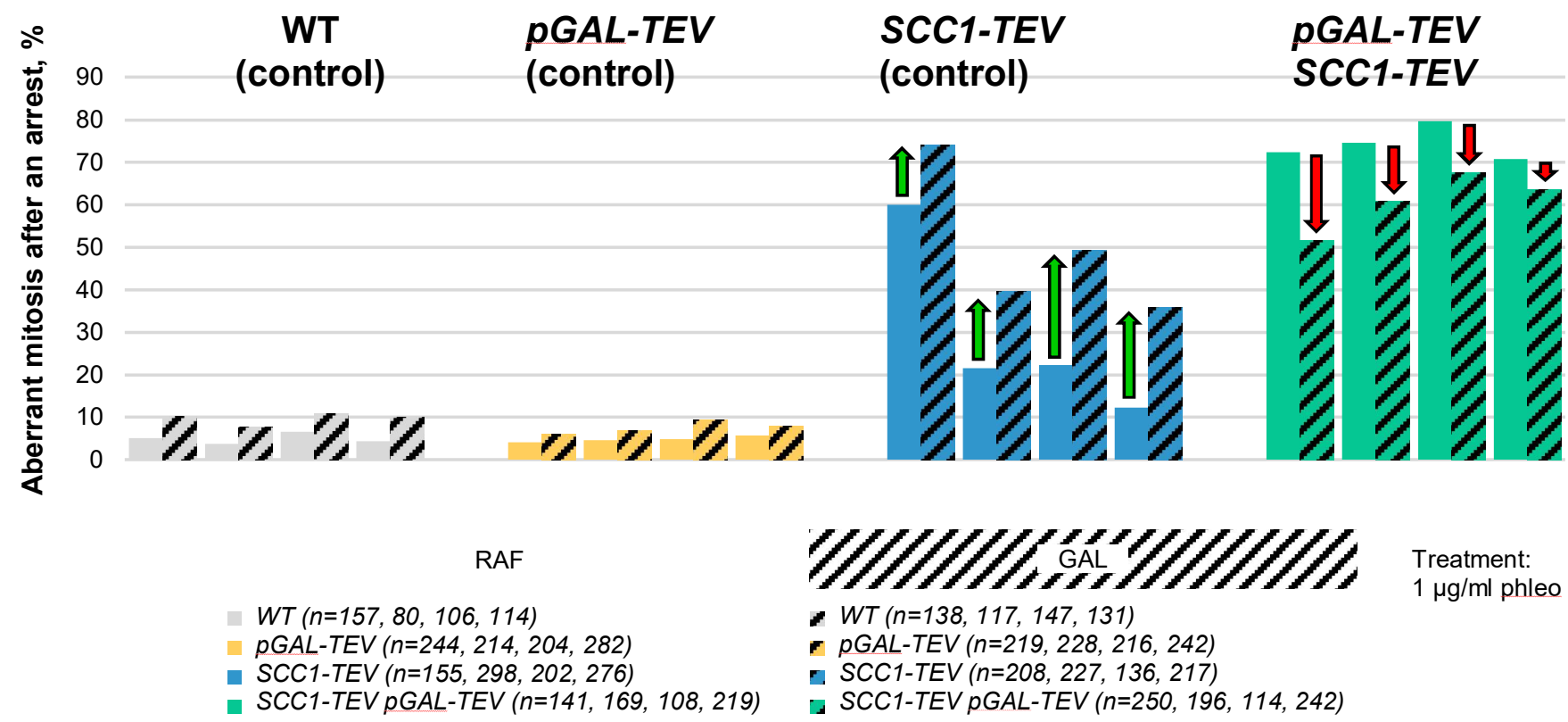

Figure R1-1. Analysis of biological repeats individually showed that the probability of arrested cells to undergo an aberrant mitosis after inducing artificial cohesin cleavage was always decreasing, whereas adding galactose led to an increase for other populations. A total number of completed mitotic events after an arrest (n) scored for each repeat is stated in the legend. Arrows highlight increase or decrease in the frequency only for *SCC1-TEV and SCC1-TEV pGAL-TEV* mutants.

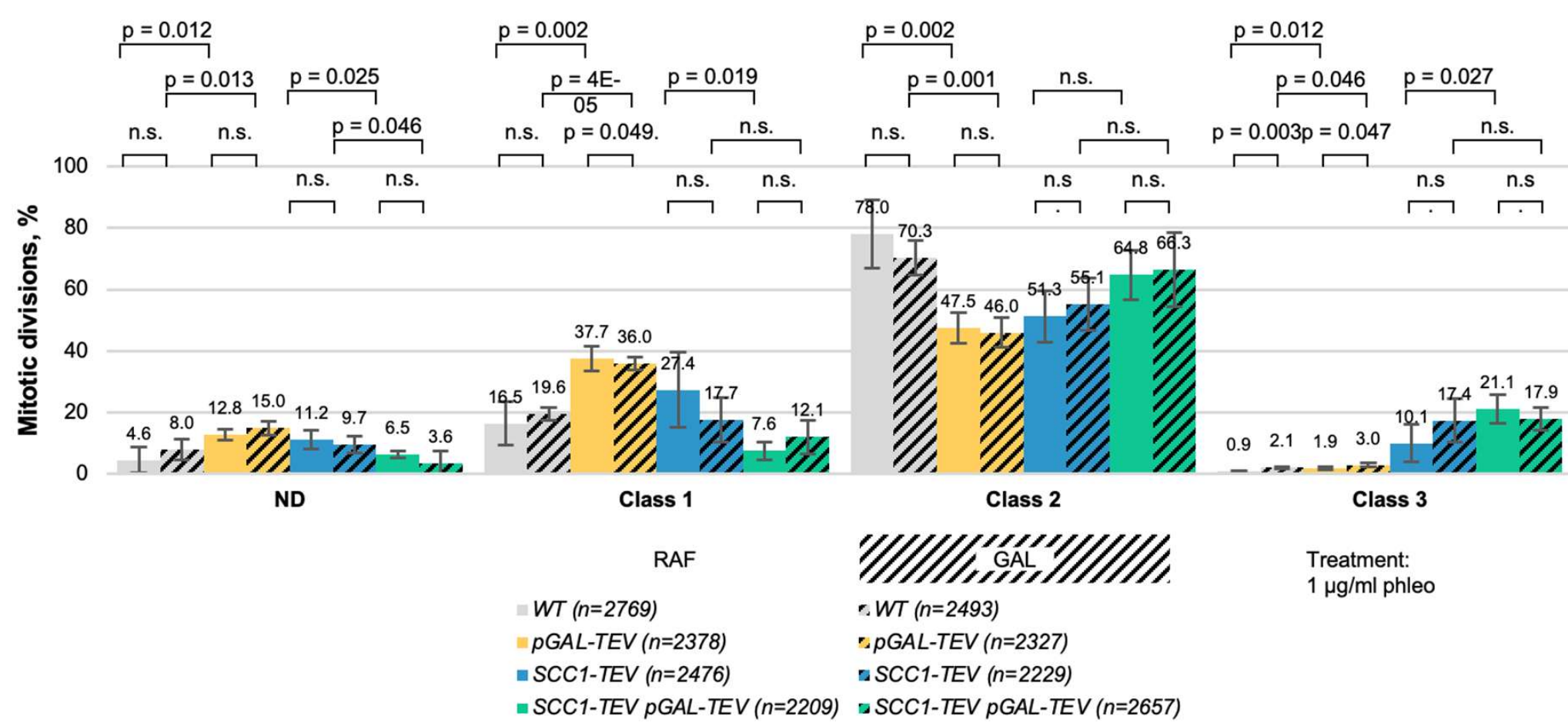

Figure R1-2. Inducing TEV protease to cleave Scc1-TEV during the phleomycin-induced arrest did not demonstrate a statistically significant decrease in the frequency of aberrant mitosis, but the effect might be shadowed by the one caused by adding galactose. Averages (shown above each bar) of at least 3 biological repeats are plotted for each genotype. The error bars represent st.dev. A total number of mitotic events (n) scored for each genotype in all the repeats is stated in the legend.

Minor comments

5. The link between the prolonged DNA damage arrest and aberrant mitoses here seems to be the Pds1 phosphorylation by Chk1, and consequent Pds1 stabilization. However, I feel that this finding is diluted in the results section. A change in the data presentation order may help bringing the result forward.

Wang et al showed that the Chk1-dependent phosphorylation is required for the accumulation and this is their finding. Ours is that this accumulation leads to aberrant mitoses, hence the focus is more on this.

6. The text and main figure (Figure 2) explaining the classes of aberrant mitoses are difficult to follow. Representative time-lapse image series and graphs from Figure S1 are more complete and easier to interpret, and could be moved into the main figure instead.

We moved figure S1 into the main figures as suggested.

7. Dark orange bars in Figures 3A and 3F are not indicated in the legend. These type of graphs are also difficult to read due to overlapping data series. It would be better to represent the data in a different way- ie. By showing individual data series separately.

We converted each plot of this kind into a series of 3 or 4 plots to simplify the presentation of the data as suggested (Figure 5A and 5F).

8. The data presented in Figure 3B are not clearly explained.

The figure legend has been extended to address this point (Figure 5B now)

9. Striped or dotted graph fill patterns should be avoided, as they are barely visible, especially in legends.

We replaced the dotted patterns and left thick stripes for double mutants only, where the 2 colours are always a combination of the colours of the corresponding single mutants.

10. The discussion could be more concise.

It has been shortened significantly in the revised version

11. One-way ANOVA should be used when comparing more than two samples.

The stats have been re-done as suggested and the p-values replaced.

12. Nuclear divisions occurring within one cell compartment (Figure 5) resemble failures of the spindle position checkpoint (SPOC), which normally inhibits mitotic exit and cytokinesis when spindle elongation into the bud fails. This checkpoint arrests cells in M phase. Interestingly, the most downstream SPOC component (Bfa1) is also required for cell cycle arrest in response to telomere dysfunction. This raises the question of whether Bfa1 is required to arrest these cells with compromised telomeres in G2/M or M phase. Could the authors elaborate on this?

Based on what we observe, the spindle elongates into the bud as the nucleus is transferred into the bud during the arrest. The failure to segregate comes from the lack of pulling half of the nucleus back to the mother. The pulling back occurs in Class 1 mitoses, but not in the cells with aberrant mitoses. Bfa1 is certainly required to minimise the aberrant mitoses, but it may come from Bfa1 being required to maintain the arrest in general rather than the wrong positioning of the spindle (Figure S4).

Reviewer #1 (Significance (Required)):

This study will be of interest to researchers working on DNA damage response, cell cycle regulation, telomere biology, and genome stability. It may also engage broader audiences in aging, cancer biology. The yeast telomerase insufficiency (TI) model developed here is a valuable tool for mimicking mammalian cells in which expression of the telomerase catalytic subunit TERT is downregulated during development. Conceptually, the study highlights parallels between yeast and mammalian systems, suggesting evolutionary conservation of quality-control mechanisms that eliminate compromised cells.

Reviewer expertise: Cell cycle control, checkpoints.

Reviewer #2 (Evidence, reproducibility and clarity (Required)):

In this manuscript, the authors analyse the effects of a decrease in telomerase in budding

yeast S. cerevisiae on G2/M cell arrest. Using different immunofluorecent tools and time lapse microscopy together with different genetic approaches, they show that telomere insufficiency correlates with cytokinesis problems together with accumulation Pds1 resulting in increased aneuploidy. They conclude that longer arrests may eliminate genetically unstable cells.

The consequences of telomerase deficiency have been extensively studied (work by the labs of Teixeira, Lingner, Shore, Zakian, Wellinger, etc). At the single-cell it has been already shown that senescence varies considerably from one cell to another, that cells can switch from rapid proliferation to a prolonged cell cycle in a single division, and that long cell cycles precede cell death (Xu et al 2014, Coutelier, et al 2018).

This work aims to explore the results of a decrease in telomerase expression and to study the mechanisms responsible for cycle defects. In this sense, this work deserves publication, but at this stage, the manuscript needs to be improved at many levels to make it appropriate for publication despite the impressive large number of experimental data. The article is quite dense and could be made easier to the reader by clearly highlighting the novelty brought to the field. A way to consider could be to focus on some aspects of the different data, or even considering splitting the data in two publications?

Below are a few points to consider strengthening the impact of this work.

1. The authors use an unusual method in yeast where conditional promoters (GAL, tetO2, CUP1) are well controlled. As a result, the position of the Hygromycin cassette in the EST2 promoter must be improved in the text and figure. Above all, expression levels are not visible on the Western blot, must be quantified by RT-qPCR, and must be shown for all constructs. (both expression and growth). In Fig. 1C, it appears that the control comes from a different gel, which should be avoided. In Fig.1F how is cell size calculated?

We avoided using the mentioned controlled promoters because they are leaky and the leaky expression might be comparable to the EST2 low level expression. In addition, these sort of on/off promoters have a high degree of stochasticity in a population which would complicate the analysis at the single cell level. Our approach results in a more homogeneous population of cells with a different degree of weakened expression. It worked well not only for EST2 but also for PDS1 expression.

We attempted to follow Reviewer's advice to quantify the EST2 expression using RT-qPCR. Total RNA was purified from the WT, est2p-17 and est2p-30 cells prior to any signs of growth defect due to short telomeres. cDNA was made by 2 separate approaches: either using oligo-dT or random priming. For oligo-dT, the qPCR oligos were designed at the 3' end of EST2 and ACT1. For the random priming, the qPCR oligos were selected within the first 500 bp of both genes. 3 biological repeats were performed for each genotype (3

independently isolated mutant derivatives for either genotype and independent 3 cultures of WT parental strain grown from single colonies). The relative ratio of EST2/ACT1 is plotted below. Unexpectedly, these ratios don't correspond to what we observe on Western blots or phenotypically, because they show an increase in transcripts corresponding to the EST2 region. Unfortunately, this approach doesn't allow us to distinguish if the transcripts are on EST2 coding or non-coding strand. At that point, we decided to stop further investigation as it was leading us away from the focus of this manuscript. We didn't include these data either.

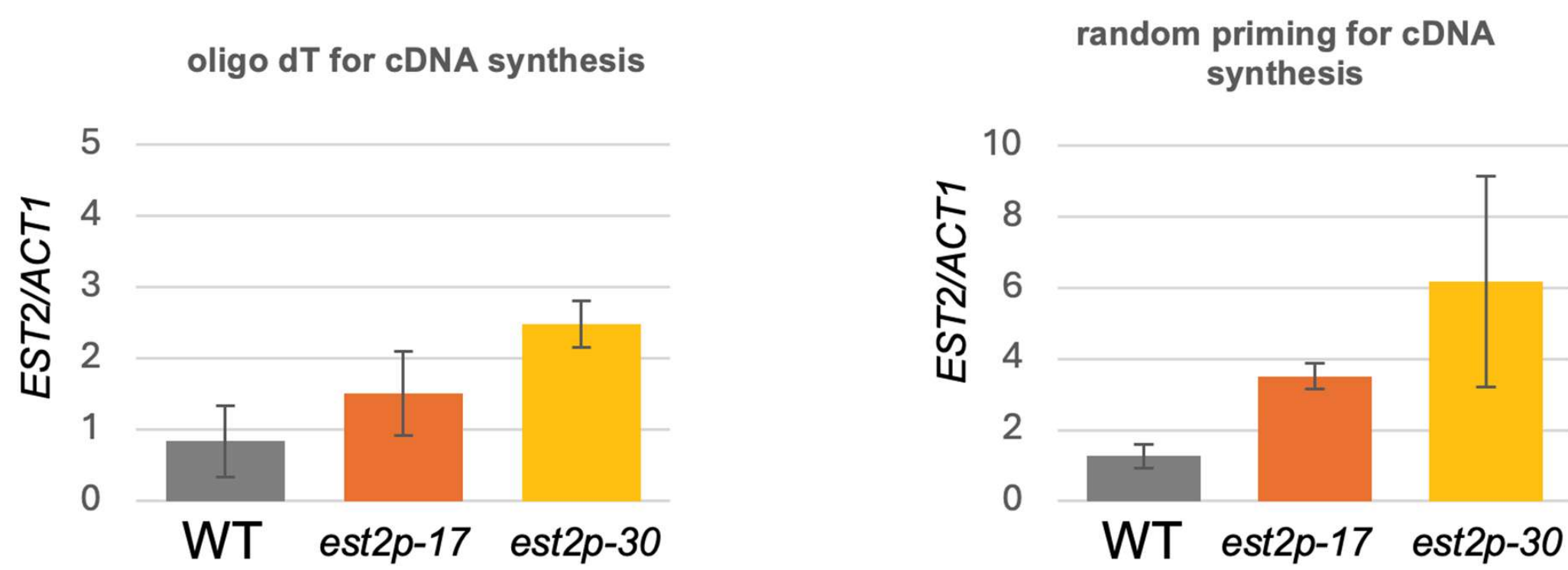

Figure R2. The effect of *est2p-17* and *est2p-30* on transcription in the EST2 locus. RNA corresponding to *EST2* locus sequences was normalized to ACT1 transcripts by RT-qPCR.

What is important is that the Est2 levels are down (Figure 1C, also the well-established phenotype of short telomeres and growth defect) and at the same time they remain above zero as the telomere structure is different from the cells with EST2 deletion. To make this more clear, we edited the text to stress this and also added a Southern blot image in Figure 1A showing examples of telomere structure in telomerase-positive cells and telomerase-negative survivors, Type I and Type II.

Actin control in Figure 1C comes from a different blot because the molecular weight of Act1 protein is much smaller than Est2-13myc and requires a higher gel percentage. In addition, much less protein sample needs to be loaded for Act1 to preserve the linearity of the signal for a LiCor -based quantification analysis. However, both gels were loaded in parallel using the exact same set of samples (just different volumes) and run in the same gel box, etc all the way to both blots being scanned together (see the Figure 1 Source File where the two gels are shown scanned side by side).

Cell area was calculated by marking cell borders with the polygon selection tool in the Fiji software and measuring the area within them. It is described in the Materials and Methods.

2. The estimated probabilities of class 3 events assume that a class 2 event becomes either a class 1 event or a class 3 event, but do not consider the fact that class 2 events can also lead to cell death. How do the authors account for this bias? Can the authors explain why they chose to perform 5 Z-stacks of 0.6 µm without projection? Z-projection imaging might be more accurate for the analyses.

> *We agree with the Reviewer that taking into account potential cell death as an additional possible outcome of Class 2 events complicates the issue with anticipated Class 3 percentages. Therefore, we removed all the charts of this kind and edited the text accordingly. The original charts with the Class 2 included still allow to analyse the actual Class 3 percentages.*
>
> *As for the microscopy analysis, we found that moving across different imaging planes for every event instead of using Z-projection gave a clearer picture of the events inside the cells. This approach was used for analysing nuclear morphology, not to quantify a fluorescent signal.*

3. The conclusion of the experiments in Fig. 3 is that DNA damage and decreased expression of est2 (if well documented, see comment 1) are comparable. Suppression by Δ rad9 must be shown in both cases. To confirm that G2/M arrest is due to DNA damage, analysis of H2A phosphorylation or Rad53 phosphorylation would be beneficial. The conditions for phleomycin treatment must be specified (time). The graphs in Fig. 3A, 3B and 3F could be made easier to understand. What are the percentages calculated on?

> *The Rad9 involvement (as well as Rad53 phosphorylation) in the cell cycle arrest in response to both DNA breaks and compromised telomeres (cells with telomerase deficiency) has been extensively characterised in the literature (For example Ijpma and Greider, 2003). The key downstream component of the DNA damage signalling pathway analysed here is Pds1 (Securin) which we assayed for both TI and DNA breaks (Phleo), as shown in the revised Figure 6A-B. Given the focus of the manuscript on aberrant mitoses and not as much on the upstream signalling leading to Pds1 phosphorylation, we believe that the pds1-m9 analysis is both more informative, than rad9 and rad53 would be, and sufficient to draw a parallel between TI and DSBs, which has been established and well documented for DSBs and telomerase loss.*

4. The conclusions concerning Pds1 should be interpreted with caution, as it has not been proven that aberrant mitoses are caused by the accumulation of Pads1; only a

correlation has been demonstrated. We propose to add control strains (WT, Δest2, WT treated with phleomycin). The measurement should be performed on more than 15 cells, and the method should be explained in the Materials and Methods section. Finally, accumulation should be quantified by RT-qPCR to consolidate the results.

We believe the data in Figure 6E(revised version numbering) establish the cause-effect relationship between the expression levels of Pds1 and aberrant mitoses. We show that reducing PDS1 expression partially suppresses aberrant mitoses.

The analysis of Pds1 levels at the single cell level has been extended to more cells and to Class1 and Class 3 comparison (Figure 6C-D).

We don't think that the proposed RT-qPCR for PDS1 expression is appropriate here. It can't be performed at the single cell level. At the populational level, it won't add anything to what we know already, because the Pds1 protein dynamics in cells with DNA damage have already been analysed (Wang et al, 2001).

5. The loss of coordination between nuclear division and cytokines is interesting but needs to be supported by quantification and controls (WT, Δest2, MEN mutants).

The quantifications for est2p-30 have been added to the figure legend (Figure 7). For WT cells, such analysis would be close to technically impossible to do as the Class 3.1 events are extremely rare under normal growth conditions (<0.1%).

We are not sure we understand what the Reviewer meant by listing MEN mutants and Δest2 as controls and what aspects these would be controlling. The Δest2 mutants behave depending on the stage of senescence (Figure 4) and it would be a very laborious experiment to analyse the mutants through different stages of senescence. Class 3.1 events are quite rare (0.8% in est2p-30) and would be even more rare at the early stages of senescence. In addition, we could catch the Myo2 ring contraction in-progress in only a fraction of Class 3.1 events with the 5 min imaging interval.

Minor comments

The authors should make an effort to cite recent work in the field and position their current analysis accordingly, and make sure references are not missing (for instance type I and type II survivors). It would be helpful to also improve the concision and the clarity of the results section, with concluding sentences for each result.

Work could be done on the figures to simplify them. In particular, the bar charts are quite complicated to understand given all the mutations that have been made and the fact that they are each subdivided into classes 1, 2, and 3.

We made sure concluding sentences are present at the end of the paragraphs in the Results, as suggested by the Reviewer. The charts with the anticipated Class 3 percentages have been removed. The text was also edited further to improve clarity. A few more recent references have been added.

Reviewer #2 (Significance (Required)):

This work aims to explore the results of a decrease in telomerase expression and to study the mechanisms responsible for cycle defects. In this sense, this work deserves publication, but at this stage, the manuscript needs to be improved at many levels to make it appropriate for publication despite the impressive large number of experimental data. The article is quite dense and could be made easier to the reader by clearly highlighting the novelty brought to the field. A way to consider could be to focus on some aspects of the different data, or even considering splitting the data in two publications?

We agree that there are a lot of different experiments here and we considered a possibility of splitting the story into two as suggested. However, we don't see two clear messages to have one for each of the separate papers. As is, there is a very clear message and this tells us to keep it as is. We tried to clarify the text to help navigating through our data as much as possible.

Specialized audience.

Our expertise in DNA repair and yeast genome organization allows us to offer what we hope are constructive comments on this work, even though not telomere specialists.

Reviewer #3 (Evidence, reproducibility and clarity (Required)):

In this manuscript, Dubenko and colleagues investigated, at a single-cell level, how yeast cells regulate cell cycle arrest and recovery in response to DNA breaks or telomere dysfunctions, as well as the potential outcomes for cells that resume cell cycle

progression after arrests of varying durations. They used Saccharomyces cerevisiae populations in which telomere defects were induced by downregulating telomerase expression, while DNA breaks were generated through phleomycin treatment. Time-lapse microscopy was employed to measure the duration of checkpoint-induced cell cycle blocks, nuclear division, and the localization of key proteins and structures in individual cells, with the goal of exploring heterogeneity in the DNA damage response and the relationship between cell cycle arrest and chromosome stability.

To monitor nuclear dynamics, the authors generated yeast strains with reduced telomerase expression carrying GFP-tagged histones and mCherry-labeled Nop56 for time-lapse imaging. Both telomerase downregulation and phleomycin treatment led to an increased frequency of aberrant DNA segregations following G2/M cell cycle arrests. This frequency was higher in cells experiencing prolonged arrests. Elevated levels of Pds1 were observed in cells with extended checkpoint activation, whereas partial loss of Pds1 function reduced the frequency of abnormal segregations but increased the incidence of aneuploidy.

Analysis of mitotic spindle dynamics through time-lapse microscopy indicated that the spindle becomes unstable in cells undergoing prolonged cell cycle arrest. Furthermore, mutations that delayed cytokinesis decreased the occurrence of aberrant mitoses, while mutations promoting premature activation of MEN had the opposite effect, increasing aberrant mitoses.

Overall, the authors concluded that sustained activation of the DNA damage checkpoint leads to mitotic defects, aneuploidy, and cell death over a few divisions in cells that take too long to repair DNA damage. They proposed that this mechanism could eliminate cells with unstable genomes.

Major comments:

The data and the methods presented in such a way that they can be reproduced.

The experiments are adequately replicated and statistical analysis adequate.

Most of the key conclusions are convincing, particularly those concerning the relationship between prolonged cell cycle arrest and mitotic defects, the role of Pds1, and the coordination between chromosome segregation and mitotic exit/cytokinesis. However, while the authors propose that prolonged arrests and mitotic defects may occur in cells that take too long to repair DNA damage, they did not directly assess DNA damage persistence.

> *We added a dataset analysing mitoses in cells with unrepairable HO-induced DSBs where the damage foci are visualised with Rad52 (Figure 5G). While we understand that this is a somewhat different setting, it eliminates the repair factor (unrepairable break) and allows to see aberrant mitoses as a result of cell division in the presence of DNA damage.*

Another important point that, in my opinion, need further investigation is the impact of EST2 downregulation on cell growth kinetics and telomere integrity.

This is shown in Figure 1F-H as the increase in doubling time and large cells is a well-documented consequence of dysfunctional telomerese which are recognised as DSBs and trigger DNA damage response accompanied by a G2/M arrest.

The extent of telomerase downregulation, as well as the persistence of telomerase activity in TI strains, was not clearly evaluated.

We tried to evaluate the degree of downregulation by qRT-PCR but it looks like transcription in the EST2 locus is not straight forward to address – see the response to Reviewer 2 comments. The presence of telomerease activity in TI strains is evidenced by their preserved telomere structure which is different from the telomerase-deficient cells. We explained this more in the text for the readers not specialising in telomere research.

Additional experiments suggested:

1. I think that the effect of EST2 downregulation is not entirely clear. The authors showed that Est2 protein was undetectable by western blot, but telomeres from TI cells appear to be at least partially functional. However, the Southern blot in Fig. 1D lacks critical controls, such as est2Δ telomeres and type I and type II survivors, which are necessary for proper interpretation.

We added an example of a teloblot (telomere-specific Southern) for WT and Type I and Type II telomerase deficient survivors to Figure 1A, with the corresponding explanations in the figure legend. This should help reading the blots in Figure 1D-E.

2. -Most of the analyses were performed after 200 PD, but it is unclear what happens before this point. Do the cells experience growth defects or a senescence-like phenotype soon after TI? How do telomeres behave before reaching equilibrium?

Several independent clones were analysed by teloblot through the passaging and a representative blot is added to figure 1 (Figure 1C). The clones behaved the same way. The telomeres shortened in the first few passages, as expected, and then stayed short and stable for the rest of the passaging.

3. -Fig. 1C: at what time point were Est2 levels evaluated? Is there any change or increase in Est2 levels over time?

The levels were evaluated right after reducing the telomerase expression levels, i.e. the earliest after introducing the est2 mutations, in order to avoid the complications caused by the changes in cell cycle and cell morphology profiles in response to compromised telomeres – the cell cycle profile shifting to G2/M and the cell size increases, which are both likely to affect the ratio of nuclear proteins to actin in general. By avoiding this, one could compare the levels of Est2 protein in cells with TI to the WT levels more accurately. A sentence on this has been added to the figure legend.

4. -Figs. 1C-F: est2p-17 and est2p-30 cells show undetectable Est2 protein levels, similar telomere structures, and accumulation of large cells, yet they form colonies differently on agar plates. What explains these differences? Have the authors analyzed the growth kinetics in these strains?

The growth kinetics is shown in Fig 1F-H. As for the explanation in the colony size, the est2p-17 have a larger fraction of cells experiencing G2/M arrest (see Fig 2C in the revised version), a smaller fraction of cells with Normal Divisions than est2p-30 cells and also longer duration of the arrests on average (est2p-17 have a larger fraction of Class 2 cells than est2p-30 cells, which is an indirect representation of the duration of arrests: the longer the arrests are on average, the larger fraction of cells will be scored as arrested at the end of the video). These two factors result in the longer population doubling time (Figure 1H) and therefore a slower growth on agar plates (Fig 1F).

5. Finally, in the discussion, the authors claim that telomerase activity is present in TI cells, but they did not directly assess telomerase activity. This would require further clarification.

The telomerase activity is clearly present in the cells with TI because the telomeres maintain the short and stable structure (Fig 1C-D). This is different from the cells completely lacking telomerase activity where the switching to recombination-dependent telomere maintenance leads to very clear changes in telomere structure (Figure 1A right). The telomeres are short in the est2p mutants, which suggests that telomerase is less active, i.e. the telomere extension is limited. This is also consistent with the reduced levels of Est2 by Western blotting (even though we can't see the protein – it is lower than the detection threshold). Since Est2 is a well-established catalytic subunit of the telomerase enzyme, we think it is pretty straight forward to talk about telomerase activity in vivo, even though we don't directly measure it, but

rather <u>use in vivo telomere length as a readout</u> and rely on what has been established about Est2 as an enzyme carrying the telomerase catalytic activity.

I think these experiments are realistic and could be done in few months.

Minor comments:
Specific experimental issues that are easily addressable:
6. -Fig. 3C: RAD9 deletion leads to a reduction in both cell cycle prolongation and aberrant mitosis in TI cells. What is its impact on cell viability? Viability can be assessed either by colony formation assays or by time-lapse experiments.

The relevant data have been added as Figure 9C, not only for rad9 mutants but also for bfa1, lte1 and pds1-m9.

7. - How do other mutations that affect cell cycle arrest and mitotic defects in TI cells, such as the pds1 phosphorylation-defective allele or MEN mutations, impact cell viability?

The effects on the cell cycle arrest durations can be seen from the fraction of Class 2 – the higher the fraction the longer the arrests on average, or from ND fraction – the lower the ND fraction, the higher the fraction of cells undergoing G2/M arrests. The MEN mutations are presented in Figure 8B (lte1Δ extends the arrests in est2p-30) and 8C (bfa1Δ shortens the arrests in est2p-30). Also, Figure S4 further dissects how bfa1 is required for longer arrests in est2p-30. The relevant data for pds1-m9 are in figure 6A (pds1-m9 shortens the arrests in est2p-30). The viability data have been added in Figure 8D.

Prior studies referenced appropriately, but some review are quite old (e.g. Melo and Toczkski, 2002; Cesare and Reddel, 2010).

We replaced these with more recent reviews.

The text and figures are generally clear and accurate. Suggestions to improve the clarity and the readability of the figures:

We incorporated most of the suggestions below.

-Fig. 2B: Add estp-17 in the graph
-Legend strains of some graph are very small and difficult to read (e.g. Fig. 2B; 3A, C, D, E, ...)

- Suggestions that would help the authors improve the presentation of their data and conclusions:
-Page 2: Briefly describe the concept of cohesion to help readers unfamiliar with the term.
-Page 4: Clarify the difference between telomerase insufficiency and telomerase deficiency.
-Page 4: Consider moving the description of telomere structure to the Introduction for better context.
-Page 5: Specify how many population doublings (PD) correspond to "10 passages on YEPD plates."
-Page 6: Present the description of the different phenotypes observed by microscopy first, and then describe the behavior of the different strains. This structure would make the text clearer for readers.
-Page 12: Define here what is meant by "collapsed spindle."
-Page 13: Consider quantifying the frequency of collapsed spindles during the different mitotic events.
-Discussion: The discussion is too long and difficult to follow. Consider condensing and reorganizing it to improve readability.

Reviewer #3 (Significance (Required)):

This work advances our understanding of how cells respond to DNA damage. In particular, it provides insights into how individual cells within a population respond differently likely depending on the type and extent of damage they experience, thereby highlighting the heterogeneity of the DNA damage response. Such a response, on the one hand, coordinates cell cycle arrest with DNA repair, allowing cells sufficient time to remove the damage and subsequently progress through cell divisions with an intact genome. On the other hand, when this response persists, it promotes mitotic catastrophes and cell death, likely preventing the expansion of cells with genetic instability.

People working od DNA damage response will be interested in this work.

My expertise:
I work on the DNA damage response in yeast, with a specific focus on checkpoint signaling and the activation of checkpoint kinases. However, I do not have sufficient expertise to fully evaluate the contribution of MEN and cytokinesis, as I am not familiar

with all the components and the complex interconnections between the pathways that regulate mitotic exit.

July 29, 2026

Re: JCB manuscript #202511106R

Svetlana Makovets
University of Edinburgh

Dear Dr. Makovets,

Thank you for submitting your revised manuscript entitled "Extended DNA damage-induced G2/M arrests lead to aberrant mitoses through accumulation of securin." The manuscript has been evaluated by the original reviewers, whose full comments are appended below. The manuscript has been seen by the original reviewers whose full comments are appended below. While the reviewers continue to be overall positive about the work in terms of its suitability for JCB, some important issues remain.

After considering both the reviewers' comments and the revisions you have already made, I believe that many of the major concerns raised during the initial review have been addressed satisfactorily. In particular, I find your interpretation regarding the lack of detectable telomerase in the promoter mutants to be reasonable, given that the residual telomerase activity observed necessarily implies the presence of residual protein, even if the abundance falls below the detection threshold of the available antibody. Likewise, because Pds1 phosphorylation is known to regulate protein stability, the conclusion that altered Pds1 accumulation and delayed removal contribute to the observed phenotype appears to be well supported by the available data.

Nevertheless, the reviewers have requested additional clarification on these points and a few others. I therefore ask that you carefully address their remaining comments, particularly by clarifying the rationale for your interpretations where appropriate. Our general policy is that papers are considered through only one revision cycle; however, given that the suggested changes are relatively minor we are open to one additional short round of revision.

Please submit the final revision within one month, along with a cover letter that includes a point by point response to the remaining reviewer comments.

Thank you for this interesting contribution to Journal of Cell Biology. You can contact me or the scientific editor listed below at the journal office with any questions at cellbio@rockefeller.edu.

Sincerely,

Daniel Lew
Monitoring Editor
Journal of Cell Biology

Therese Gerbich
Scientific Editor
Journal of Cell Biology

Reviewer #1 (Comments to the Authors (Required)):

This manuscript investigates the consequences of prolonged DNA damage checkpoint activation in budding yeast. The authors combine live-cell imaging with genetic analyses to examine how DNA damage-induced extended G2/M arrest affects mitotic progression, chromosome segregation, and cell viability. The data indicate that prolonged G2/M arrest can lead to aberrant mitoses. These aberrant mitoses subsequently result in cell death following defective chromosome segregation, including failure of proper nucleolar segregation. The authors further provide evidence that aberrant mitoses depend on mitotic exit network activation (e.g., via Lte1) followed by cytokinesis, while elevated Pds1 levels prevent Cohesin cleavage and normal chromosome segregation.
The study addresses an important biological question and the revised manuscript is substantially strengthened by the additional experiments and clarifications. Nevertheless, I believe that several issues still require clarification before publication. In particular, the manuscript remains difficult to follow, aspects of the engineered promoter alleles require further explanation, and some mechanistic interpretations appear stronger than currently supported by the data.

Major comments
1. The strategy of reducing EST2 (and PDS1) expression by inserting antibiotic-resistance cassettes into their promoters is

central to the manuscript. How long is the inserted cassette, what is the position of the insertion relative to the transcription start site - rather than the translation start site? Can authors clarify these. Also, a comparison of results from complete gene deletion phenotypes or other promoter switch or a degron-strategies could be presented where available. These points becomes particularly relevant in light of the confusing RT-qPCR results.
2. Figure 4 nicely illustrates the relationship between passage number and the frequency of aberrant mitoses. How does passage number influence the frequency of aberrant mitoses in the telomerase insufficiency model used throughout the manuscript? It would be helpful if the authors clarified how passage number was controlled or standardized across experiments using the TI strains.
3. Since Class 3 events occur at relatively low frequencies (<10% in many analyses; e.g., Figs. 2C, 5C, 5D, 5G, 6A, 6B and 6E), it is sometimes difficult to judge the biological relevance of relatively small but statistically significant differences. Although I appreciate the large number of individual cells analyzed, it is not clear how much variability exists between independent microscopy experiments. Presenting biological replicates, or otherwise indicating experiment to experiment variability, would help assess the robustness of these findings. In addition, the frequency of Class 3 events among cells that experienced prolonged arrest (e.g., Figs. 2D and 5D) appears to provide a more informative and comparable representation of the data, which should be presented for all similar type of data.
4. Analyses of Pds1 dynamics showed that Pds1 is eventually degraded prior to aberrant mitosis. While the genetic evidence clearly supports a role for Pds1 in promoting these events, it is however less clear mechanistically how transiently elevated Pds1 levels predispose cells to chromosome segregation defects - not only from the perspective of sister chromatid cohesion but also from the perspective of FEAR and MEN networks. I therefore encourage the authors to present this aspect of the model somewhat more cautiously. Along the same lines, I suggest using the term "stopwatch" more cautiously, as it implies a mechanistic understanding that is not yet fully established by the data.
5. The authors employ several approaches to induce prolonged DNA damage checkpoint activation, including telomerase insufficiency (est2p-30), telomerase deletion (est2Δ), phleomycin treatment, and HO-induced double-strand breaks. Interestingly, all approaches except the subtelomeric HO-induced break generate aberrant mitoses. It would be helpful if the authors discussed how this observation fits with the overall conclusions and what distinguishes the subtelomeric break from the other models. In addition, DNA damage checkpoint dependence is demonstrated only in the est2p-30 model (e.g via rad9Δ). Clarifying whether the same requirement applies to the other damage models would strengthen the conclusion that aberrant mitoses arise through a common checkpoint-dependent mechanism rather than reflecting model-specific effects. Finally, because similar phenotypes are observed following multiple forms of DNA damage, the manuscript should place greater emphasis on prolonged DNA damage checkpoint activation as the common biological principle rather than focusing on telomerase insufficiency.
6. The observation that nucleolar segregation is defective in est2p-30 cells is particularly interesting. Is this phenotype unique to the telomerase insufficiency model, or is it also observed following phleomycin treatment or HO-induced DNA breaks? In addition, in the HO-induced DSB experiments, following Rad52 foci allows the analysis to focus specifically on cells experiencing DNA damage. Could a comparable strategy be applied in the telomerase insufficiency and phleomycin models to distinguish cells that have activated a DNA damage response from those that have not? Such an approach might reduce heterogeneity within the analyzed population and facilitate more direct comparisons between the different DNA damage models.

Minor comments
1. What is t0 in Figure 6C. Please specify. As this is a reference point and I suppose individual cell data are also aligned with respect to this time point - this should be explained.
2. Page 6 (lines 153-158): the text refers to Southern blot analysis of telomere length, but the corresponding figure is missing.
3. Page 6 (lines 167-168): the text refers to "Normal Divisions (ND; Fig. 2A-B)", but ND is not indicated in Figure 2B.
4. In Figures 6A and 6B, the reduction in Class 2 events is also apparent and could be mentioned in the text.
5. In Figure 2A, the examples of Classes 3.1-3.3 are difficult to interpret in the final time points, particularly because the bright-field image obscures the GFP signal. It is difficult to distinguish between complete absence of DNA and the presence of a very small DNA fragment.
6. In Figures 1H and 9C, several horizontal reference lines appear slightly tilted.

Reviewer #2 (Comments to the Authors (Required)):

This manuscript investigated the consequences of prolonged cell cycle blocks on mitosis and genome stability in Saccharomyces cerevisiae at a single-cell level. Cell cycle arrests are induced both by DNA damage and dysfunctional telomeres, obtained by downregulating telomerase expression. By following chromatin and nucleolar marks in time-laps microscopy, the authors define three classes of aberrant mitosis events and correlate the fraction of these events with the extent of cell cycle arrest, showing an increased frequency of aberrant DNA segregations after prolonged G2/M arrests. Cells experiencing aberrant mitosis have high levels of Pds1. The frequency of aberrant mitosis decreases in the absence of a functional checkpoint or in the presence of a phosphorylation-defective pds1 allele. It also decreases when mitotic exit is delayed by the lack of Lte1, while it increases when mitotic exit is anticipated by the lack of Bfa1. The authors conclude that DNA damage checkpoint-dependent accumulation of Pds1 and loss of coordination between chromosome segregation and mitotic exit cause aberrant mitosis in cells with prolonged cell cycle arrests and results in aneuploidy and cell death.
The manuscript is well written, and the rationale underlying the experimental design is clearly described and logically presented. The experiments are well done, the experimental data are convincing, and the conclusions are well supported by the presented results. This work advances our understanding of the cellular response to DNA damage by highlighting the heterogeneity of

individual cell responses.
Overall, I believe that this manuscript is suitable for publication in Journal of Cell Biology. However, before it can be accepted, some aspects of the experimental model used for telomerase downregulation should be clarified.
Specifically:
-The Est2 protein is undetectable in est2p-17 and est2p-30 mutants in western blot in fig. 1C but telomeres appear to be more stable than telomeres in est2Delta cells, suggesting that telomerase is still active in est2p mutants, as also the authors propose. Does overexposure of the gel reveal basal Est2 protein levels? Have the protein levels been assessed over time? Has the EST2 transcript level been assessed in the mutants?
-Does the est2p-30 mutant exhibit growth defects or a senescence-like phenotype? Figure 1F shows that est2p-30 cells are viable after 10 passages. Do these cells maintain viability without loss of fitness in earlier passages?

Minor comments:
-lines 117-122: a reference should be provided for the description of Type I and Type II survivors; the authors could also refer the reader to figure 1.
- include molecular weight (size) markers in the Southern blot in fig. 1A
- the kinetics of telomere shortening in est2p-17 and est2p-30 cells in Figure 1 should be compared with the kinetics observed in est2Delta cells in Figure 4.
- A duplicated parenthesis appears on line 299
-specify that loss of BFA1 "led to increased cell death" at line 406

Reviewer #3 (Comments to the Authors (Required)):

The study by Dubenko et al. highlights a new mechanism in which the accumulation of Pds1 acts as a molecular clock during prolonged G2/M cell cycle arrests, causing an increase in aneuploid cells and a dysregulation of cytokinesis, leading to abnormal mitoses. Using real-time microscopy with Pds1-tdTomato and mitotic markers, the authors demonstrate that longer arrests correlate with a delay in Pds1 degradation. Genetic manipulations (pds1-m9, lte1Δ, bfa1Δ) confirm the central role of Pds1.
Although the mechanistic approach is innovative, the real-time microscopy study is robust and the authors have made considerable efforts to improve image quality, the TI model requires clarification, and two main points need to be addressed. The current evidence for reduced telomerase activity in est2p strains is insufficient, as the RT-qPCR data provided to the reviewers were inconclusive and the Western blots show only undetectable levels of Est2 without directly confirming residual activity (it should be noted that p $(dN)_6$ to generate cDNA might have been preferable to oligodT primers).
I believe that the findings regarding Pds1, although supported by the revised version of this study - which examined a larger number of cells and employed appropriate statistical tests - show that when Pds1 expression is reduced, the suppression of aberrant mitoses decreases only very slightly (from 10.3% to 6.9%), and it is only when Pds1 phosphorylation is inhibited that mitotic divisions decrease from 8% to 0.1%, which indeed suggests that Pds1 phosphorylation could play an important role. The causal relationship between Pds1 expression levels and aberrant mitoses on the contrary does not appear to have been fully established, and the title therefore seems to go beyond what has been demonstrated experimentally.

Responses to the Reviewers' comments

We would like to thank all the reviewers for the comments and suggestions. Our responses to those are in blue below.

Reviewer #1 (Comments to the Authors (Required)):

This manuscript investigates the consequences of prolonged DNA damage checkpoint activation in budding yeast. The authors combine live-cell imaging with genetic analyses to examine how DNA damage-induced extended G2/M arrest affects mitotic progression, chromosome segregation, and cell viability. The data indicate that prolonged G2/M arrest can lead to aberrant mitoses. These aberrant mitoses subsequently result in cell death following defective chromosome segregation, including failure of proper nucleolar segregation. The authors further provide evidence that aberrant mitoses depend on mitotic exit network activation (e.g., via Lte1) followed by cytokinesis, while elevated Pds1 levels prevent Cohesin cleavage and normal chromosome segregation.
The study addresses an important biological question and the revised manuscript is substantially strengthened by the additional experiments and clarifications. Nevertheless, I believe that several issues still require clarification before publication. In particular, the manuscript remains difficult to follow, aspects of the engineered promoter alleles require further explanation, and some mechanistic interpretations appear stronger than currently supported by the data.

Major comments
1. The strategy of reducing EST2 (and PDS1) expression by inserting antibiotic-resistance cassettes into their promoters is central to the manuscript. How long is the inserted cassette, what is the position of the insertion relative to the transcription start site - rather than the translation start site? Can authors clarify these. Also, a comparison of results from complete gene deletion phenotypes or other promoter switch or a degron-strategies could be presented where available. These points becomes particularly relevant in light of the confusing RT-qPCR results.

*In response to this comment, we have added enough all the relevant information for anyone to be able to reconstruct the exact sequences of every EST2 locus mutation used to create TI phenotypes. In the Methods (see **Yeast strains, oligonucleotides and plasmids**), we referred to the published template HYG cassette sequences (pFA6a-hphMX6, see Table S3) and the long primers with the sequences provided in Table S2, which were used to amplify the HYG cassette and insert it into the EST2 promoter locus. The same can be done for the PDS1 promoter mutations as the sequences of the corresponding oligos are provided in Table S2 as well.*

We have not tried using the degron system for Est2 because it would have been necessary to tune the degron to a specific level of telomerase and keep it active all the time during the cell manipulations – even a short-term inactivation of the degron would result in telomere elongation and alleviation of the TI phenotype. Any slight

change in the activity of the degron due to a batch-to-batch variation in auxin or other degron inducer, media composition or growth conditions might have resulted in the changes in the Est2 activity. In addition, the degron tag itself might have affected Est2 in some unknown ways. Notice, that even though we used myc-tag to assay Est2 levels in response to the promoter mutations, the TI strains which were used for microscopy didn't have any tags fused to Est2, to keep the aa sequence native. These reasonings led us to deciding against using a degron system and in favour of manipulating the EST2 promoter instead.

2. Figure 4 nicely illustrates the relationship between passage number and the frequency of aberrant mitoses. How does passage number influence the frequency of aberrant mitoses in the telomerase insufficiency model used throughout the manuscript? It would be helpful if the authors clarified how passage number was controlled or standardized across experiments using the TI strains.

Because aberrant mitoses are caused by short telomeres, we would expect the increase in aberrant mitoses to proceed slightly slower with each early passage than in est2Δ cells and plateau at the levels characterised in the manuscript, following the telomere dynamics shown in Figure 1D.

*Passaging of yeast while their telomeres shorten is a very straight forward well-established method in the yeast telomere field: cells are passaged onto fresh agar media at the same time interval, normally 48 h for YPD, could be longer for minimal media or if other sugars are used in rich media as a source of energy. We added a section in the Methods called **Cell passaging** providing sufficient details to reproduce this method without going into the published literature.*

3. Since Class 3 events occur at relatively low frequencies (<10% in many analyses; e.g., Figs. 2C, 5C, 5D, 5G, 6A, 6B and 6E), it is sometimes difficult to judge the biological relevance of relatively small but statistically significant differences. Although I appreciate the large number of individual cells analyzed, it is not clear how much variability exists between independent microscopy experiments. Presenting biological replicates, or otherwise indicating experiment to experiment variability, would help assess the robustness of these findings. In addition, the frequency of Class 3 events among cells that experienced prolonged arrest (e.g., Figs. 2D and 5D) appears to provide a more informative and comparable representation of the data, which should be presented for all similar type of data.

We added an Excel file as part of Supplemental data, to list all the primary data we used for making plots in all the figures. The actual number of different mitotic events scored in every single repeat of every experiment in this publication can be tracked in this file. This would allow any sort of additional data analysis with the primary data.

4. Analyses of Pds1 dynamics showed that Pds1 is eventually degraded prior to aberrant mitosis. While the genetic evidence clearly supports a role for Pds1 in promoting these events, it is however less clear mechanistically how transiently

elevated Pds1 levels predispose cells to chromosome segregation defects - not only from the perspective of sister chromatid cohesion but also from the perspective of FEAR and MEN networks. I therefore encourage the authors to present this aspect of the model somewhat more cautiously. Along the same lines, I suggest using the term "stopwatch" more cautiously, as it implies a mechanistic understanding that is not yet fully established by the data.

We removed the term stopwatch with regard to Pds1 and only kept it referring to p53-53BP1-USP28 as published by the authors of this study.

5. The authors employ several approaches to induce prolonged DNA damage checkpoint activation, including telomerase insufficiency (est2p-30), telomerase deletion (est2Δ), phleomycin treatment, and HO-induced double-strand breaks. Interestingly, all approaches except the subtelomeric HO-induced break generate aberrant mitoses. It would be helpful if the authors discussed how this observation fits with the overall conclusions and what distinguishes the subtelomeric break from the other models.

We added the following sentence explaining the Results on the subtelomeric break: "This behaviour could be explained by weaker checkpoint activation from DNA resection generating significant stretches of ssDNA only on one side of the break: the DNA fragment on the other side of HO cut is very short and degraded completely."

The Discussion also contains an explanation of this difference in the behaviour of the cells with a subtelomeric break.

In addition, DNA damage checkpoint dependence is demonstrated only in the est2p-30 model (e.g via rad9Δ). Clarifying whether the same requirement applies to the other damage models would strengthen the conclusion that aberrant mitoses arise through a common checkpoint-dependent mechanism rather than reflecting model-specific effects. Finally, because similar phenotypes are observed following multiple forms of DNA damage, the manuscript should place greater emphasis on prolonged DNA damage checkpoint activation as the common biological principle rather than focusing on telomerase insufficiency.

We modified the first para of the Discussion by adding a sentence emphasizing this particular message as suggested by the reviewer.

We do stress this particular message starting from it being in the title of the manuscript. The rad9-dependence here is not as critical as the pds1-m9-dependence, which is shown for both TI and Phleomycin-induced arrests (Figure 6A-B). Because the Pds1 phosphorylation is known to be induced in response to DNA damage, then, indeed, the DNA damage per se rather than TI specifically, is responsible for the aberrant mitosis phenotype.

Our model in Figure 8 doesn't refer to TI either. Instead, it emphasises the G2/M arrests as the trigger of the downstream events leading to aberrant mitoses.

6. The observation that nucleolar segregation is defective in est2p-30 cells is particularly interesting. Is this phenotype unique to the telomerase insufficiency

model, or is it also observed following phleomycin treatment or HO-induced DNA breaks? In addition, in the HO-induced DSB experiments, following Rad52 foci allows the analysis to focus specifically on cells experiencing DNA damage. Could a comparable strategy be applied in the telomerase insufficiency and phleomycin models to distinguish cells that have activated a DNA damage response from those that have not? Such an approach might reduce heterogeneity within the analyzed population and facilitate more direct comparisons between the different DNA damage models.

Defective nucleolar segregation has been also observed in ageing mother cells (Crane et al., 2029) as mentioned in the Discussion.

Rad52 is not the best marker for DDR activation, Rad53-phosphorylation is and the system for monitoring it in live cells has been published. We looked into it at some point but chose to prioritise other experiments. Monitoring DDR activation at a single cell level in our already quite complicated system is something that may require a lot of optimising and perhaps could be done in further studies.

Minor comments

1. What is t0 in Figure 6C. Please specify. As this is a reference point and I suppose individual cell data are also aligned with respect to this time point - this should be explained.

In figures 6C-D, time 0 is defined as the maximum separation of the nuclei following the cell division in the previous cell cycle. This note has been added to the Figure 6C legend.

2. Page 6 (lines 153-158): the text refers to Southern blot analysis of telomere length, but the corresponding figure is missing.

Added figure reference.

3. Page 6 (lines 167-168): the text refers to "Normal Divisions (ND; Fig. 2A-B)", but ND is not indicated in Figure 2B.

Corrected the figure.

4. In Figures 6A and 6B, the reduction in Class 2 events is also apparent and could be mentioned in the text.

Edited accordingly.

5. In Figure 2A, the examples of Classes 3.1-3.3 are difficult to interpret in the final time points, particularly because the bright-field image obscures the GFP signal. It is difficult to distinguish between complete absence of DNA and the presence of a very small DNA fragment.

We added references to specific frames in the text (below is the edited para from the manuscript):

Depending on the observed nuclear dynamics during aberrant mitoses, we defined 3 subclasses in Class 3, from 3.1 to 3.3 (Figure 2A-B). Class 3.1 included mitotic events with either a completed or an attempted and reversed nuclear division within a single cell compartment (normally inside the bud, Figure 2A, Class 3.1, frame 4:05) resulting in one cell retaining the majority of the DNA in the form of a single nucleus or two nuclei, while the other cell inherited very little or no visible DNA. Class 3.2 divisions were characterised by a completely skipped nuclear division resulting in a cell with the majority of the duplicated genome, predominantly ending up in daughter cells, and an anuclear cell (Figure 2A, Class 3.2, frames 2:15 and 2:35). In both Class 3.1 and 3.2, the genome-containing daughters re-budded, arrested in the next cell cycle again (Figure 2A: Class 3.1 frames 4:40 and 5:10; Class 3.2 frames 3:15 and 8:25) and in a vast majority of cases died, whereas the mothers remained cadavers with little DNA content. Class 3.3 included any cell divisions resulting in cells with more than two nuclei generated during a single mitosis (Figure 2A, Class 3.3 frame 2:55).

In Class 3.1, the key time frames are 3-5 where the nuclear division inside a single cell compartment is observed. The following 2 frames are shown purely to see the cell re-budding, not the nuclear dynamics.

In Class 3.2, the key frame is 2:35 where the cell with GFP/chromatin is re-budding and the other cell from the previous cell cycle has very little if any GFP signal.

In the class 3.3, the last frame is critical, where 3 blobs of GFP are clearly seen within a single cell.

6. In Figures 1H and 9C, several horizontal reference lines appear slightly tilted.

These problems incurred while the system was converting our figures to pdf. Hopefully, they disappear when the journal handles the original figure files which don't have tilted lines.

Reviewer #2 (Comments to the Authors (Required)):

This manuscript investigated the consequences of prolonged cell cycle blocks on mitosis and genome stability in Saccharomyces cerevisiae at a single-cell level. Cell cycle arrests are induced both by DNA damage and dysfunctional telomeres, obtained by downregulating telomerase expression. By following chromatin and nucleolar marks in time-laps microscopy, the authors define three classes of aberrant mitosis events and correlate the fraction of these events with the extent of cell cycle arrest, showing an increased frequency of aberrant DNA segregations after prolonged G2/M arrests. Cells experiencing aberrant mitosis have high levels of Pds1. The frequency of aberrant mitosis decreases in the absence of a functional checkpoint or in the presence of a phosphorylation-defective pds1 allele. It also decreases when mitotic exit is delayed by the lack of Lte1, while it increases when mitotic exit is anticipated by the lack of Bfa1. The authors conclude that DNA damage checkpoint-dependent accumulation of Pds1 and loss of coordination between chromosome segregation and mitotic exit cause aberrant mitosis in cells with prolonged cell cycle arrests and results in aneuploidy and cell death.
The manuscript is well written, and the rationale underlying the experimental design is clearly described and logically presented. The experiments are well done, the experimental data are convincing, and the conclusions are well supported by the presented results. This work advances our understanding of the cellular response to DNA damage by highlighting the heterogeneity of individual cell responses.
Overall, I believe that this manuscript is suitable for publication in Journal of Cell Biology. However, before it can be accepted, some aspects of the experimental model used for telomerase downregulation should be clarified.
Specifically:
-The Est2 protein is undetectable in est2p-17 and est2p-30 mutants in western blot in fig. 1C but telomeres appear to be more stable than telomeres in est2Delta cells, suggesting that telomerase is still active in est2p mutants, as also the authors propose. Does overexposure of the gel reveal basal Est2 protein levels? Have the protein levels been assessed over time? Has the EST2 transcript level been assessed in the mutants?

The Western blot imaging was done using the Licor technology. Trying to adjust visual settings of the image scan didn't result in Est2 band appearance in the TI mutants. The protein levels were assessed immediately after the introduction of est2-TI alleles to avoid the effect of the mutations on cell fitness.

EST2 transcription is known to be low, even in the WT cells. For this reason, we think that using Northern blotting wouldn't have helped.

-Does the est2p-30 mutant exhibit growth defects or a senescence-like phenotype? Figure 1F shows that est2p-30 cells are viable after 10 passages. Do these cells maintain viability without loss of fitness in earlier passages?

The TI mutants lose the cell fitness after a few passages and then stay as a slower-growing population, as shown by the increased population doubling time (Figure 1H), but they don't go through the cell viability crisis characteristic of the cells with a complete loss of telomerase after a telomerase gene deletion. This is consistent with

the telomeres preserving their original structure as the cell viability crisis in the telomerase-deficient cells is always followed by a switch to a recombination-dependent mode of telomere maintenance, either Type I or Type II. The TI cells don't reach this stage, rather they manage to keep the telomeres short and stable enough to keep the population away from switching to recombination at telomeres.

Minor comments:
-lines 117-122: a reference should be provided for the description of Type I and Type II survivors; the authors could also refer the reader to figure 1.

Added a reference as suggested.

- include molecular weight (size) markers in the Southern blot in fig. 1A

The blot is figure 1A is provided purely for illustrative purposes to introduce the band patterns characteristic of WT, Type I and Type II telomeres. Both sides of the image were used for labelling different structural elements which are easily identifiable in the other Southern blots which do contain the size marker. Adding a size marker to this blot would clutter the labelling around it.

- the kinetics of telomere shortening in est2p-17 and est2p-30 cells in Figure 1 should be compared with the kinetics observed in est2Delta cells in Figure 4.

Added a sentence in the text comparing the two types of mutants.

- A duplicated parenthesis appears on line 299

Corrected here in elsewhere in the text.

-specify that loss of BFA1 "led to increased cell death" at line 406

Corrected as suggested.

Reviewer #3 (Comments to the Authors (Required)):

The study by Dubenko et al. highlights a new mechanism in which the accumulation of Pds1 acts as a molecular clock during prolonged G2/M cell cycle arrests, causing an increase in aneuploid cells and a dysregulation of cytokinesis, leading to abnormal mitoses. Using real-time microscopy with Pds1-tdTomato and mitotic markers, the authors demonstrate that longer arrests correlate with a delay in Pds1 degradation. Genetic manipulations (pds1-m9, lte1Δ, bfa1Δ) confirm the central role of Pds1.
Although the mechanistic approach is innovative, the real-time microscopy study is robust and the authors have made considerable efforts to improve image quality, the TI model requires clarification, and two main points need to be addressed.
The current evidence for reduced telomerase activity in est2p strains is insufficient,

as the RT-qPCR data provided to the reviewers were inconclusive and the Western blots show only undetectable levels of Est2 without directly confirming residual activity (it should be noted that p $(dN)_6$ to generate cDNA might have been preferable to oligodT primers).

As we responded after the first round of reviews, the phenotype of TI mutants, namely the lack of the switch to recombination at telomeres, strongly argues for the presence of the residual telomerase in the TI mutants, even though the Est2 levels are below the detection with the available antibody. In addition, using TI mutants is only a tool in this study, to create a cell population with a constitutive DNA damage. The manuscript contains other systems paralleling TI mutants – cell growth in the presence of phleomycin, single inducible DSB, telomerase deletion – each as a trigger of DNA damage response and G2/M arrest. For these reasons, we think further work into characterising TI per se would not add anything significant to the main message of this study.

I believe that the findings regarding Pds1, although supported by the revised version of this study - which examined a larger number of cells and employed appropriate statistical tests - show that when Pds1 expression is reduced, the suppression of aberrant mitoses decreases only very slightly (from 10.3% to 6.9%), and it is only when Pds1 phosphorylation is inhibited that mitotic divisions decrease from 8% to 0.1%, which indeed suggests that Pds1 phosphorylation could play an important role. The causal relationship between Pds1 expression levels and aberrant mitoses on the contrary does not appear to have been fully established, and the title therefore seems to go beyond what has been demonstrated experimentally.

The low degree of reduction of aberrant mitoses could be explained by the importance of the Pds1 phosphorylation, which might affect the protein levels to a higher degree than the Pds1 reduction we achieved by perturbing PDS1 promoter. Indeed, if one compares the western blots of Pds1-WT and Pds1-m9 in the arrested cells (Wang et al., 2001), it looks like the protein levels differ around 10-fold while the PDS1 promoter mutation affects the level only about 2-fold. However, it is important that lowering the Pds1 levels did affect aberrant mitoses in the way consistent with our hypothesis. Mutants with reduced expression of Pds1 can accumulate phosphorylated Pds1 slower and therefore partially rescue the phenotype of aberrant mitoses. It is noteworthy that when cells continuously expressed the phosphorylatable, but non-degradable Pds1-destruction-box mutants no anaphase or telophase cells were observed, but a small population of aploid cells emerged (Cohen-Fix et al., 1996). This was essentially the phenotype of aberrant mitoses, we have thoroughly investigated here.

August 21, 2026

RE: JCB Manuscript #202511106RR

Svetlana Makovets
University of Edinburgh

Dear Dr. Makovets:

Thank you for submitting your revised manuscript entitled "Extended DNA damage-induced G2/M arrests lead to aberrant mitoses through accumulation of securin". Based on your revisions, we would be happy to publish your paper in JCB pending final revisions necessary to meet our formatting guidelines (see details below). In your final revision, please be sure to include the support tables you reference which do not appear to have been uploaded to our system.

To avoid unnecessary delays in the acceptance and publication of your paper, please read the following information carefully.

A. MANUSCRIPT ORGANIZATION AND FORMATTING:

Full guidelines are available on our Instructions for Authors page, http://jcb.rupress.org/submission-guidelines#revised.
Submission of a paper that does not conform to JCB guidelines will delay the acceptance of your manuscript.

1) Text limits: Character count for Articles is < 40,000, not including spaces. Count includes abstract, introduction, results, discussion, and acknowledgments. Count does not include title page, figure legends, materials and methods, references, tables, or supplemental legends.

Currently ~42k, please try to reduce this slightly.

2) Figures limits: Articles may have up to 10 main text figures.

3) Figure formatting: Scale bars must be present on all microscopy images, including inset magnifications. Molecular weight or nucleic acid size markers must be included on all gel electrophoresis. Aspect ratios of images may not be altered.

In order to accommodate readers with red-green color blindness, we ask that you please change the red/green color scheme used in the graphs in all figures and supplemental figures using these colors. Please also be sure to include scale bars on Fig 3.

4) Statistical analysis: Error bars on graphic representations of numerical data must be clearly described in the figure legend. The number of independent data points (n) represented in a graph must be indicated in the legend. Statistical methods should be explained in full in the materials and methods. For figures presenting pooled data the statistical measure should be defined in the figure legends. Please also be sure to indicate the statistical tests used in each of your experiments (either in the figure legend itself or in a separate methods section) as well as the parameters of the test (for example, if you ran a t-test, please indicate if it was one- or two-sided, etc.). Also, if you used parametric tests, please indicate if the data distribution was tested for normality (and if so, how). If not, you must state something to the effect that "Data distribution was assumed to be normal but this was not formally tested."

Please be sure that any panel containing error bars has the description of what they represent in the figure legend as well as description of any statistical tests being done.

5) Abstract and title: The abstract should be no longer than 160 words and should communicate the significance of the paper for a general audience. The title should be less than 100 characters including spaces. Make the title concise but accessible to a general readership.

6) Materials and methods: Should be comprehensive and not simply reference a previous publication for details on how an experiment was performed. Please provide full descriptions in the text for readers who may not have access to referenced manuscripts.

7) All antibodies, cell lines, animals, and tools used in the manuscript should be described in full, including accession numbers for materials available in a public repository such as the Resource Identification Portal. Please be sure to provide the sequences for all of your primers/oligos and RNAi constructs in the materials and methods. You must also indicate in the methods the source, species, and catalog numbers (where appropriate) for all of your antibodies. Please also indicate the acquisition and quantification methods for immunoblotting/western blots.

8) Microscope image acquisition: The following information must be provided about the acquisition and processing of images:

a. Make and model of microscope
b. Type, magnification, and numerical aperture of the objective lenses
c. Temperature
d. Imaging medium
e. Fluorochromes
f. Camera make and model
g. Acquisition software
h. Any software used for image processing subsequent to data acquisition. Please include details and types of operations involved (e.g., type of deconvolution, 3D reconstitutions, surface or volume rendering, gamma adjustments, etc.).

9) References: There is no limit to the number of references cited in a manuscript. References should be cited parenthetically in the text by author and year of publication. Abbreviate the names of journals according to PubMed.

10) Supplemental materials: There are strict limits on the allowable amount of supplemental data. Articles may have up to 5 supplemental figures.

At the moment, you currently have 8 such items (5 figures and 3 tables). Please either reduce or combine these items in order to meet the figure count and be sure to change any callouts in the text to reflect these changes. Please also note that tables, like figures, should be provided as individual, editable files. A summary of all supplemental material should appear at the end of the Materials and methods section.

11) eTOC summary: A ~40-50-word summary that describes the context and significance of the findings for a general readership should be included on the title page. The statement should be written in the present tense and refer to the work in the third person.

12) Conflict of interest statement: JCB requires inclusion of a statement in the acknowledgements regarding competing financial interests. If no competing financial interests exist, please include the following statement: "The authors declare no competing financial interests." If competing interests are declared, please follow your statement of these competing interests with the following statement: "The authors declare no further competing financial interests."

13) ORCID IDs: ORCID IDs are unique identifiers allowing researchers to create a record of their various scholarly contributions in a single place. Please note that ORCID IDs are now *required* for all authors. At resubmission of your final files, please be sure to provide your ORCID ID and those of all co-authors.

14) A separate author contribution section following the Acknowledgments. All authors should be mentioned and designated by their full names. We encourage use of the CRediT nomenclature.

Full guidelines are available on our Instructions for Authors page, https://jcb.rupress.org/submission-guidelines#revised.
Submission of a paper that does not conform to JCB guidelines will delay the acceptance of your manuscript.

Please note that JCB now requires authors to submit Source Data used to generate figures containing gels and Western blots with all revised manuscripts. This Source Data consists of fully uncropped and unprocessed images for each gel/blot displayed in the main and supplemental figures. For assays performed using capillary electrophoresis and/or immunoassay-based detection, authors should instead provide the electropherogram graph(s) for each experiment, plotting fluorescence/chemiluminescence intensity vs. molecular weight/size. Please be sure to provide one Source Data file for each figure gels, blots, and/or capillary electrophoresis assays along with your revised manuscript files. File names for Source Data figures should be alphanumeric without any spaces or special characters (i.e., SourceDataF#, where F# refers to the associated main figure number or SourceDataFS# for those associated with Supplementary figures). For traditional gels and blots, the lanes of the gels/blots should be labeled as they are in the associated figure, the place where cropping was applied should be marked (with a box), and molecular weight/size standards should be labeled wherever possible. For capillary electrophoresis assays, each trace in the graph should be color-coded and labeled to indicate which protein, gene, or sample is being measured (please try to avoid red/green combinations to accommodate our color-blind readers).

Source Data files will be made available to reviewers during evaluation of revised manuscripts and, if your paper is eventually published in JCB, the files will be directly linked to specific figures in the published article.

Source Data Figures should be provided as individual PDF files (one file per figure). Authors should endeavor to retain a minimum resolution of 300 dpi or pixels per inch. Please review our instructions for export from Photoshop, Illustrator, and PowerPoint here: https://rupress.org/jcb/pages/submission-guidelines#revised

Journal of Cell Biology now requires a data availability statement for all research article submissions. These statements will be published in the article directly above the Acknowledgments. The statement should address all data underlying the research presented in the manuscript. Please visit the JCB instructions for authors for guidelines and examples of statements at (https://rupress.org/jcb/pages/editorial-policies#data-availability-statement).

B. FINAL FILES:

Please upload the following materials to our online submission system. These items are required prior to acceptance. If you have any questions, contact JCB's Managing Editor, Lindsey Hollander (lhollander@rockefeller.edu).

-- An editable version of the final text (.DOC or .DOCX) is needed for copyediting (no PDFs).

-- High-resolution figure and MP4 video files: See our detailed guidelines for preparing your production-ready images, https://jcb.rupress.org/fig-vid-guidelines.

-- Cover images: If you have any striking images related to this story, we would be happy to consider them for inclusion on the journal cover. Submitted images may also be chosen for highlighting on the journal table of contents or JCB homepage carousel. Images should be uploaded as TIFF or EPS files and must be at least 300 dpi resolution.

It is JCB policy that if requested, original data images must be made available to the editors. Failure to provide original images upon request will result in unavoidable delays in publication. Please ensure that you have access to all original data images prior to final submission.

The license to publish form must be signed before your manuscript can be sent to production. A link to the license to publish form will be sent to the corresponding author only. Please take a moment to check your funder requirements before choosing the appropriate license.

Additionally, JCB encourages authors to submit a short video summary of their work. These videos are intended to convey the main messages of the study to a non-specialist, scientific audience. Think of them as an extended version of your abstract, or a short poster presentation. We encourage first authors to present the results to increase their visibility. The videos will be shared on social media to promote your work. For more detailed guidelines and tips on preparing your video, please visit https://rupress.org/jcb/pages/submission-guidelines#videoSummaries.

Thank you for your attention to these final processing requirements. Please revise and format the manuscript and upload materials within 14 days. If you need an extension for whatever reason, please let us know and we can work with you to determine a suitable revision period.

Please contact the journal office with any questions at cellbio@rockefeller.edu.

Thank you for this interesting contribution, we look forward to publishing your paper in Journal of Cell Biology.

Sincerely,

Daniel Lew
Monitoring Editor
Journal of Cell Biology

Therese Gerbich
Scientific Editor
Journal of Cell Biology
