# Supplementary material for: Extended DNA damage–induced G2/M arrests lead to aberrant mitoses through accumulation of securin
Source: J Cell Biol. 2026 Nov 2;225(12):e202511106. doi: 10.1083/jcb.202511106 (PMC13726689; doi:10.1083/jcb.202511106)
Supplement: Supplementary file 2 [file jcb_202511106_sourcedataf1.pdf]

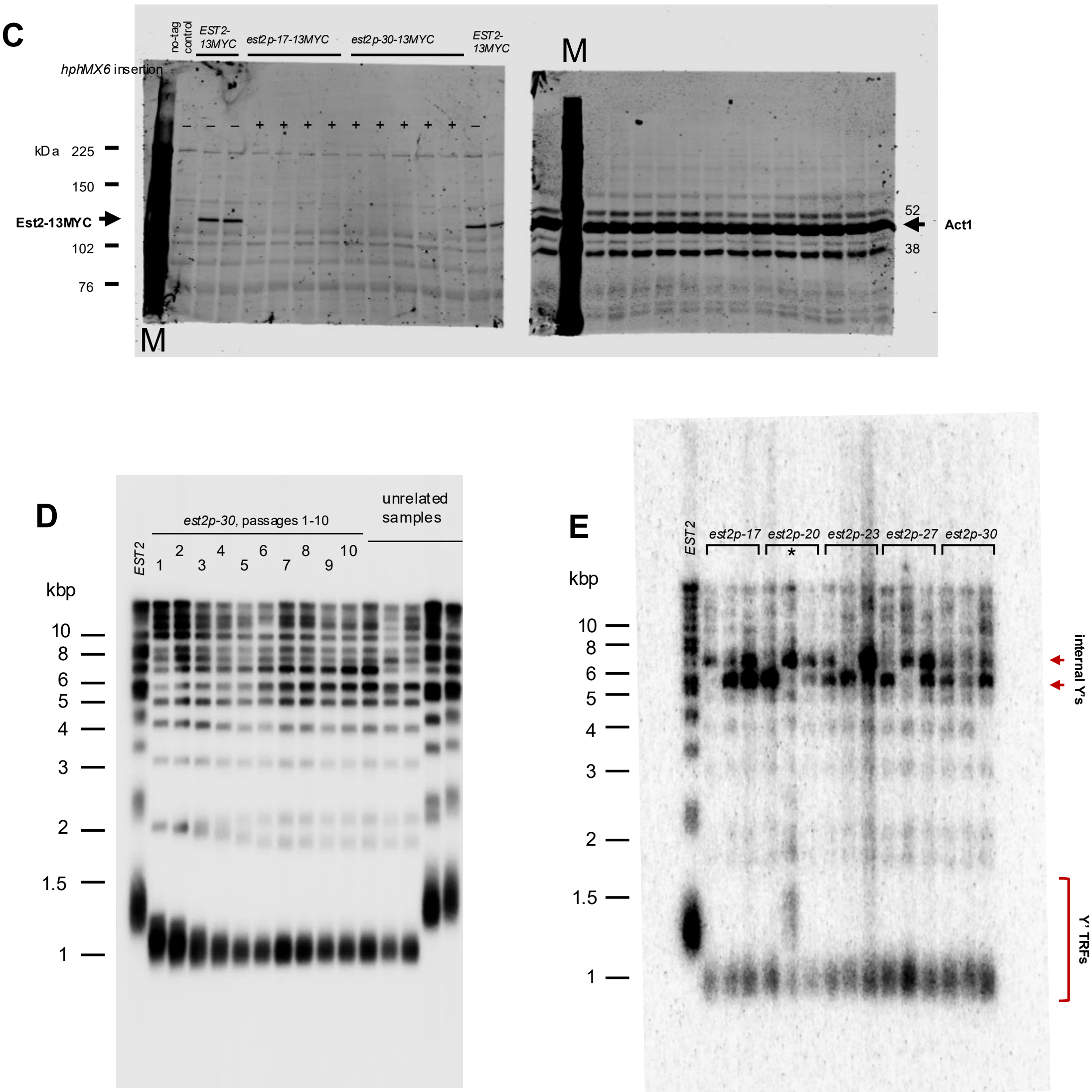

C
no-tag control
EST2-13MYC
est2p-17-13MYC
est2p-30-13MYC
EST2-13MYC
hphMX6 insertion
M
– – – + + + + + + + + + + –
kDa 225
150
Est2-13MYC
102
76
M
52
Act1
38
D
est2p-30, passages 1-10
unrelated samples
EST2
1 2 3 4 5 6 7 8 9 10
kbp
10
8
6
5
4
3
2
1.5
1
E
EST2
est2p-17
est2p-20
est2p-23
est2p-27
est2p-30
*
kbp
10
8
6
5
4
3
2
1.5
1
internal Y's
Y' TRFs
