# Supplementary material for: Extended DNA damage–induced G2/M arrests lead to aberrant mitoses through accumulation of securin
Source: J Cell Biol. 2026 Nov 2;225(12):e202511106. doi: 10.1083/jcb.202511106 (PMC13726689; doi:10.1083/jcb.202511106)
Supplement: Supplementary file 3 [file jcb_202511106_sourcedataf4.pdf]

A

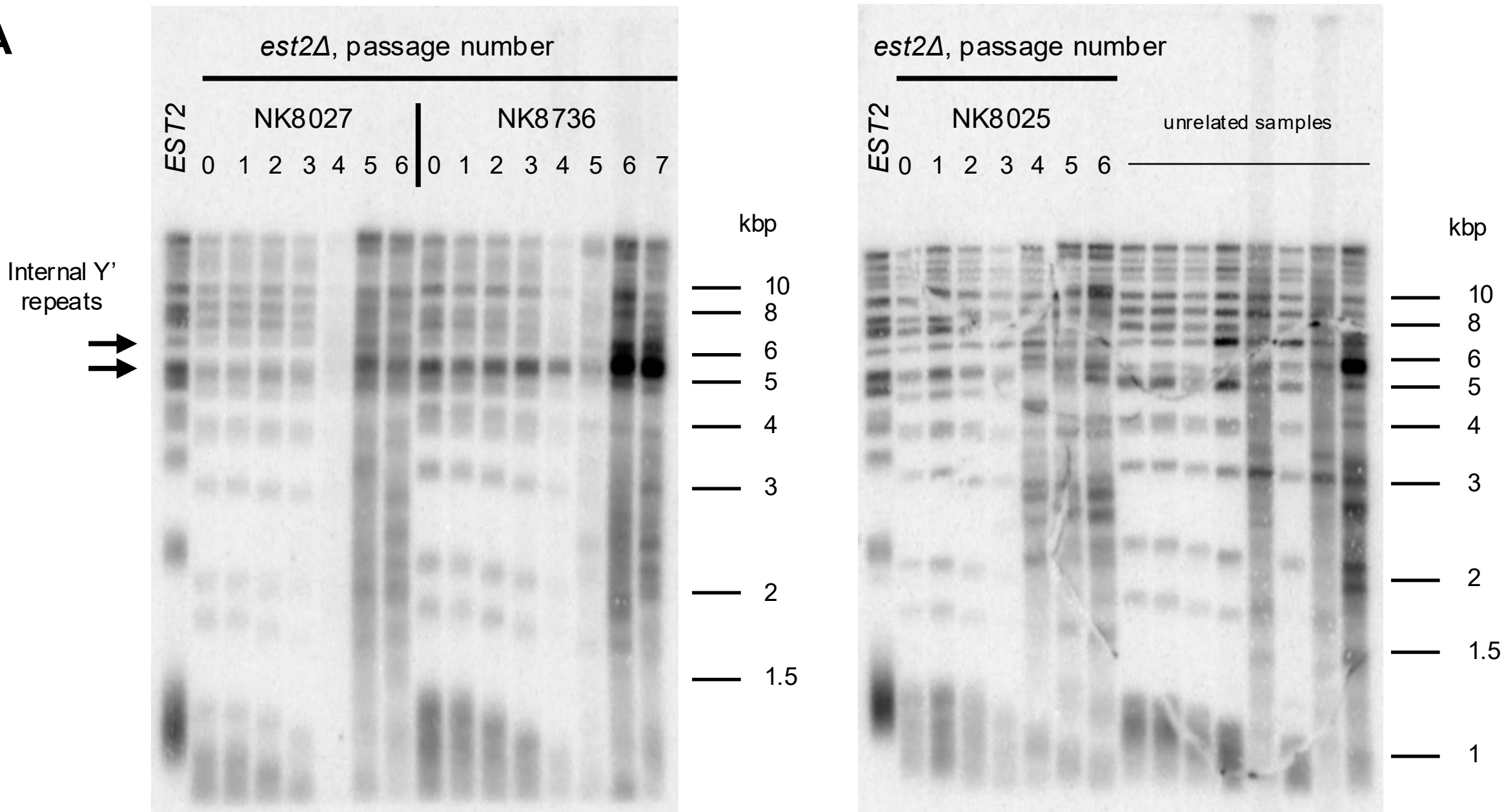

est2Δ, passage number
EST2
NK8027
NK8736
0 1 2 3 4 5 6 0 1 2 3 4 5 6 7
Internal Y' repeats
kbp
10
8
6
5
4
3
2
1.5
est2Δ, passage number
EST2
NK8025
unrelated samples
0 1 2 3 4 5 6
kbp
10
8
6
5
4
3
2
1.5
1
