# Supplementary material for: Extended DNA damage–induced G2/M arrests lead to aberrant mitoses through accumulation of securin
Source: J Cell Biol. 2026 Nov 2;225(12):e202511106. doi: 10.1083/jcb.202511106 (PMC13726689; doi:10.1083/jcb.202511106)
Supplement: Supplementary file 4 [file jcb_202511106_sourcedataf6.pdf]

E

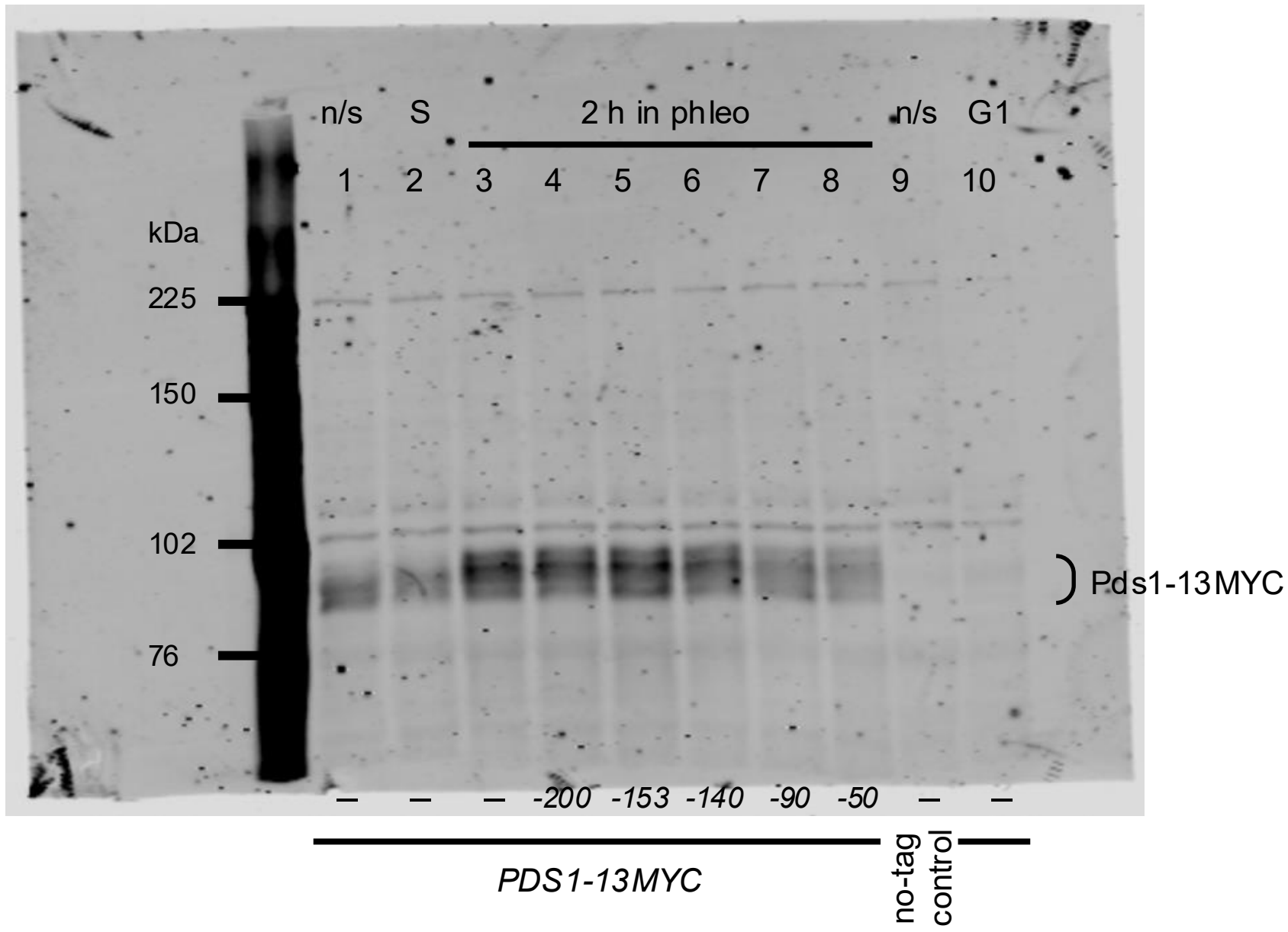

n/s
S
2 h in phleo
n/s
G1
1 2 3 4 5 6 7 8 9 10
kDa
225
150
102
76
Pds1-13MYC
– – – -200 -153 -140 -90 -50 – –
PDS1-13MYC
no-tag control
