# Supplementary material for: Extended DNA damage–induced G2/M arrests lead to aberrant mitoses through accumulation of securin
Source: J Cell Biol. 2026 Nov 2;225(12):e202511106. doi: 10.1083/jcb.202511106 (PMC13726689; doi:10.1083/jcb.202511106)

***MATa/α* 5FOA^R Met⁻**

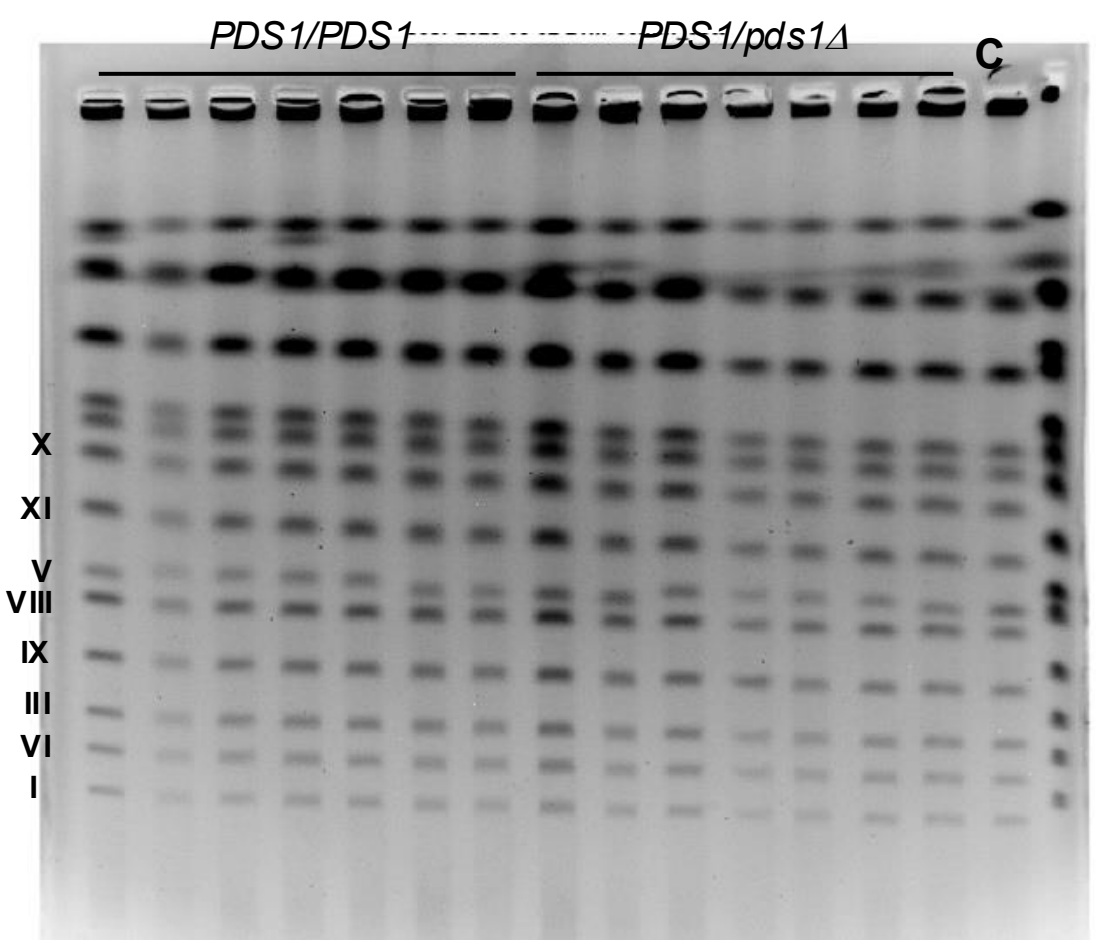

***MATa/α* Can^R Trp⁻**

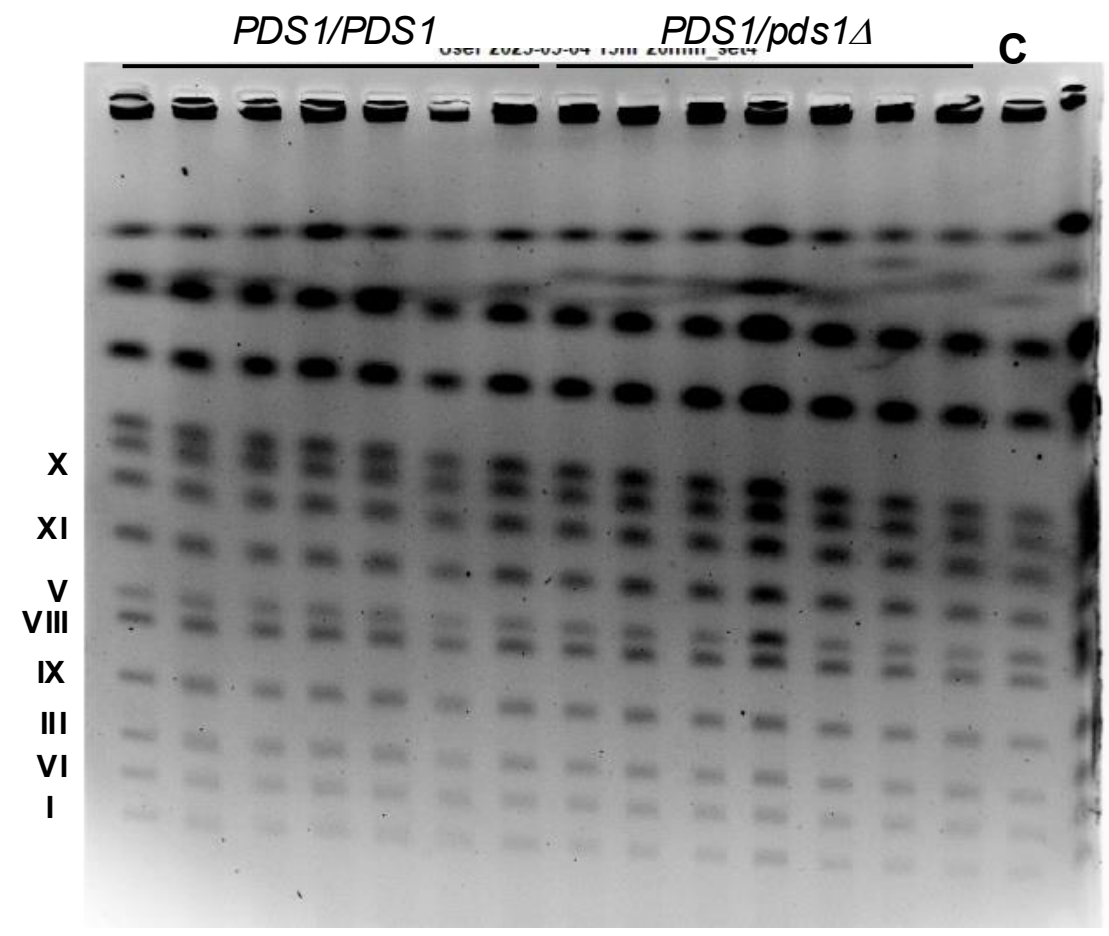

***MATΔ/α* 5FOA^R Met⁻**

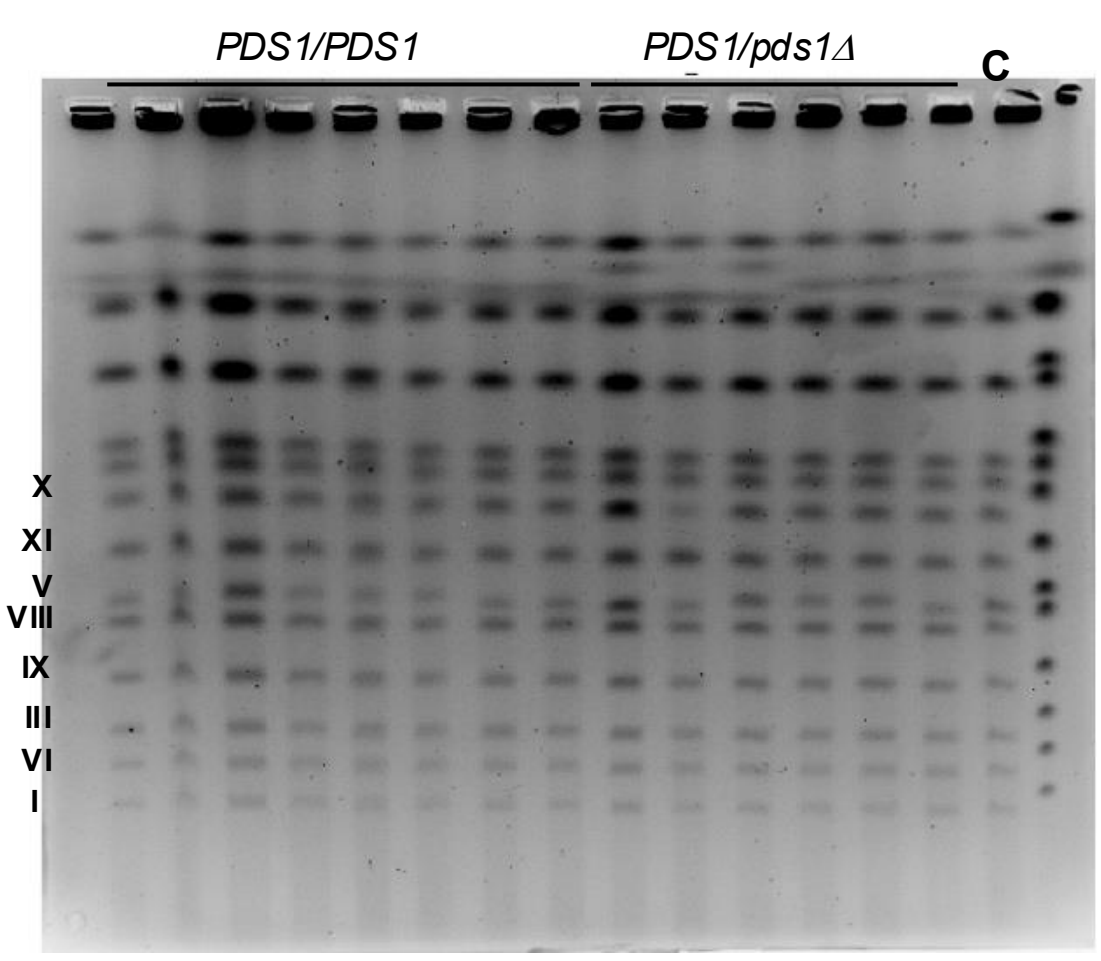

***MATΔ/α* Can^R Trp⁻**

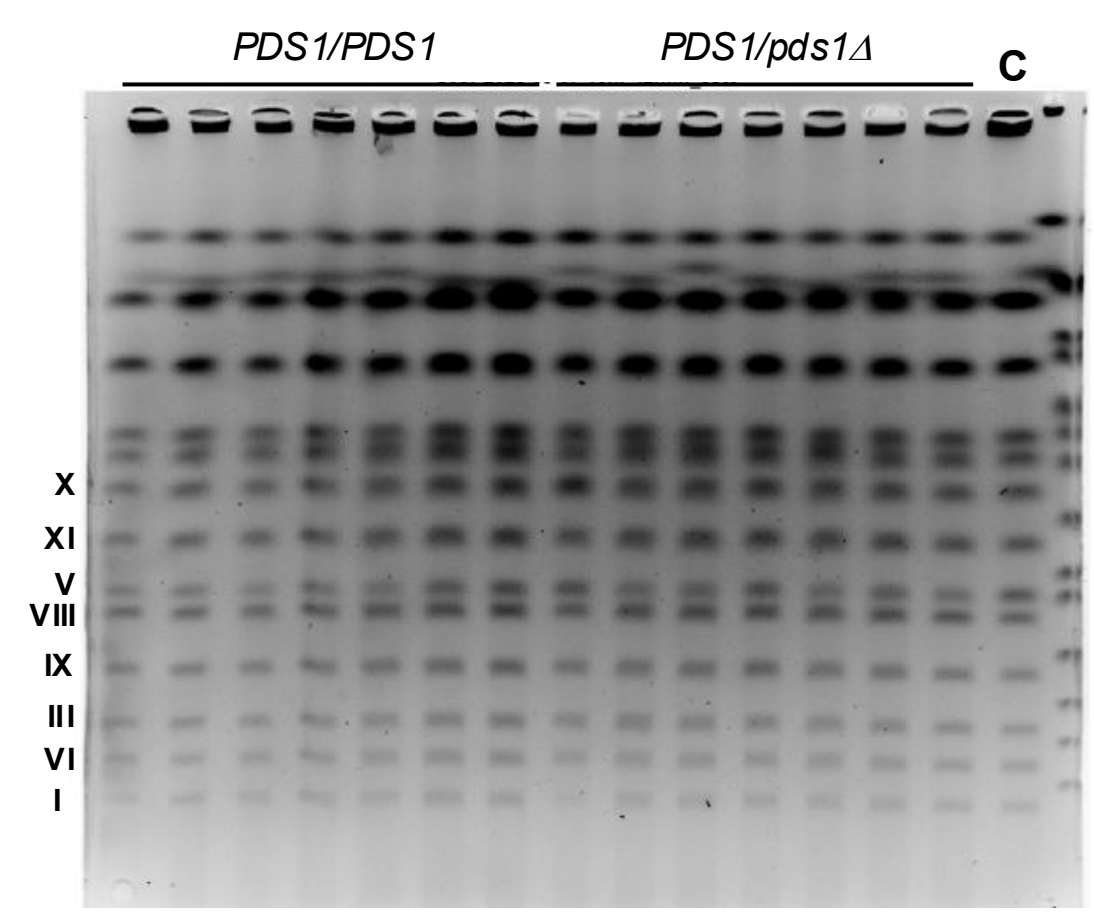

Supplement: Supplementary file 5 [file jcb_202511106_sourcedatafs3.pdf]
