# Supplementary material for: Extended DNA damage–induced G2/M arrests lead to aberrant mitoses through accumulation of securin
Source: J Cell Biol. 2026 Nov 2;225(12):e202511106. doi: 10.1083/jcb.202511106 (PMC13726689; doi:10.1083/jcb.202511106)
Supplement: Supplementary file 6 [file jcb_202511106_tables1.docx]

**Table S1. Yeast strains, oligonucleotides and plasmids used in this study.**

| A. **Yeast strains** | | | | |
| --- | --- | --- | --- | --- |
| Strain number | Genotype | | Origin/References | |
| ***Saccharomyces cerevisiae* S288c** | | | | |
| NK7300 | *MATa his3Δ1 leu2Δ0 met15Δ0 ura3Δ0 HTB2-GFP-HIS3* | | (Huh *et al.*, 2003) | |
| NK7320  NK7321  NK7322  NK7323  NK9191  NK9192  NK9193  NK9194  NK9195  NK9196  NK9197  NK9198  NK9231 | *MATa his3Δ1 leu2Δ0 met15Δ0 ura3Δ0 HTB2-GFP-HIS3 P_EST2_ Δ(-17 – -30)::HYG* | | NK7300 *P_EST2_ Δ(-17 – -30)::HYG* | |
| NK7324  NK7325  NK7326  NK7327 | *MATa his3Δ1 leu2Δ0 met15Δ0 ura3Δ0 HTB2-GFP-HIS3 P_EST2_ (-20-30)::HYG* | | NK7300 *P_EST2_ (-20-30)::HYG* | |
| NK7328  NK7329  NK7330 | *MATa his3Δ1 leu2Δ0 met15Δ0 ura3Δ0 HTB2-GFP-HIS3 P_EST2_ (-23-30)::HYG* | | NK7300 *P_EST2_ (-23-30)::HYG* | |
| NK7331  NK7332  NK7333  NK7334 | *MATa his3Δ1 leu2Δ0 met15Δ0 ura3Δ0 HTB2-GFP-HIS3 P_EST2_ (-27-30)::HYG* | | NK7300 *P_EST2_ (-27-30)::HYG* | |
| NK7335  NK7336  NK7337  NK9199  NK9200  NK9201  NK9202  NK9203  NK9204  NK9205  NK9206  NK9207 | *MATa his3Δ1 leu2Δ0 met15Δ0 ura3Δ0 HTB2-GFP-HIS3 P_EST2_ (-30 – -30)::HYG* | | NK7300 *P_EST2_ (-30 – -30)::HYG* | |
| NK8023 | *MATα his3Δ1 leu2Δ0 lys2Δ0 ura3Δ0* | | (Huh *et al.*, 2003) | |
| NK8024 | *MATa his3Δ1 leu2Δ0 met15Δ0 ura3Δ0* | | (Huh *et al.*, 2003) | |
| NK8025  NK8026  NK8027  NK8736 | *MATa his3Δ1 leu2Δ0 met15Δ0 ura3Δ0 HTB2-GFP-HIS3 est2::KAN* | | NK7300 *est2::KAN* | |
| NK8138  NK8139 | *MATa his3Δ1 leu2Δ0 met15Δ0 ura3Δ0 HTB2-GFP-HIS3 dnl4::KAN* | | NK7300 *dnl4::KAN* | |
| NK8140  NK8141 | *MATa his3Δ1 leu2Δ0 met15Δ0 ura3Δ0 HTB2-GFP-HIS3 P_EST2_ (-30 – -30)::HYG dnl4::KAN* | | NK7335 *dnl4::KAN* | |
| NK8142  NK8143  NK8144  NK8145 | *MATa his3Δ1 leu2Δ0 met15Δ0 ura3Δ0 HTB2-GFP-HIS3 P_EST2_ Δ(-17 – -30)::HYG dnl4::KAN* | | NK7322 *dnl4::KAN* | |
| NK8146 |  |  | NK7320 *dnl4::KAN* | |
| NK8224  NK8225  NK8226  NK8227  NK8228  NK8229  NK8230 | *MATa his3Δ1 leu2Δ0 met15Δ0 ura3Δ0 HTB2-GFP-HIS3 dnl4::KAN P_EST2_ Δ(-17 – -30)::HYG* | | NK8138 *P_EST2_ Δ(-17 – -30)::HYG* | |
| NK8231  NK8232  NK8233  NK8234 |  |  | NK8139 *P_EST2_ Δ(-17 – -30)::HYG* | |
| NK8235  NK8236  NK8237 | *MATa his3Δ1 leu2Δ0 met15Δ0 ura3Δ0 HTB2-GFP-HIS3 dnl4::KAN P_EST2_ (-30 – -30)::HYG* | | NK8138 *P_EST2_ (-30 – -30)::HYG* | |
| NK8238 |  |  | NK8139 *P_EST2_ (-30 – -30)::HYG* | |
| NK8755  NK8756  NK8757 | *MATa his3Δ1 leu2Δ0 met15Δ0 ura3Δ0 HTB2-GFP::HIS3 SPC42-tdTomato::NAT* | | NK7300 *SPC42-tdTomato::NAT* | |
| NK8768  NK8769  NK8770  NK8771 | *MATa his3Δ1 leu2Δ0 met15Δ0 ura3Δ0 HTB2-GFP::HIS3 SPC42-tdTomato::NAT P_EST2_ (-30 – -30)::HYG* | | NK8575 *P_EST2_ (-30 – -30)::HYG* | |
| NK8772  NK8773  NK8774  NK8775  NK8776 |  |  | NK8576 *P_EST2_ (-30 – -30)::HYG* | |
| NK8777  NK8778  NK8779  NK8780  NK8781 |  |  | NK8577 *P_EST2_ (-30 – -30)::HYG* | |
| NK8923  NK8924  NK8925  NK8926 | *MATa his3Δ1 leu2Δ0 met15Δ0 ura3Δ0 HTB2-GFP-HIS3 rad9::NAT* | | NK7300 *rad9::NAT* | |
| NK8931  NK8932  NK8933  NK8934 | *MATa his3Δ1 leu2Δ0 met15Δ0 ura3Δ0 HTB2-GFP-HIS3 rad9::NAT P_EST2_ Δ(-17 – -30)::HYG* | | NK8925 *P_EST2_ Δ(-17 – -30)::HYG* | |
| NK8935  NK8936  NK8937  NK8938 |  |  | NK8926 *P_EST2_ Δ(-17 – -30)::HYG* | |
| NK8939  NK8940  NK8941  NK8942  NK8943 | *MATa his3Δ1 leu2Δ0 met15Δ0 ura3Δ0 HTB2-GFP-HIS3 rad9::NAT P_EST2_ (-30 – -30)::HYG* | | NK8925 *P_EST2_ (-30 – -30)::HYG* | |
| NK8944  NK8945 |  |  | NK8926 *P_EST2_ (-30 – -30)::HYG* | |
| NK9290  NK9291  NK9292  NK9293 | *MATa his3Δ1 leu2Δ0 met15Δ0 ura3Δ0 HTB2-GFP-HIS3 rad9::NAT est2::KAN* | | NK8925 *est2::KAN* | |
| NK9294  NK9295 |  |  | NK8926 *est2::KAN* | |
| NK9629  NK9630  NK9631 | *MATa his3Δ1 leu2Δ0 met15Δ0 ura3Δ0 HTB2-GFP-HIS3 bfa1::KAN* | | NK7300 *bfa1::KAN* | |
| NK9632  NK9633  NK9634 | *MATa his3Δ1 leu2Δ0 met15Δ0 ura3Δ0 HTB2-GFP-HIS3 bfa1::URA3* | | NK7300 *bfa1::URA3* | |
| NK9652  NK9653  NK9654 | *MATa his3Δ1 leu2Δ0 met15Δ0 ura3Δ0 HTB2-GFP-HIS3 bfa1::KAN P_EST2_ Δ(-17 – -30)::HYG* | | NK9629 *P_EST2_ Δ(-17 – -30)::HYG* | |
| NK9659  NK9660  NK9661  NK9662 |  |  | NK9630 *P_EST2_ Δ(-17 – -30)::HYG* | |
| NK9741  NK9742  NK9743 |  |  | NK9631 *P_EST2_ Δ(-17 – -30)::HYG* | |
| NK9655  NK9656  NK9657  NK9658 | *MATa his3Δ1 leu2Δ0 met15Δ0 ura3Δ0 HTB2-GFP-HIS3 bfa1::KAN P_EST2_ (-30 – -30)::HYG* | | NK9629 *P_EST2_ (-30 – -30)::HYG* | |
| NK9663  NK9664  NK9665  NK9666 |  |  | NK9630 *P_EST2_ (-30 – -30)::HYG* | |
| NK9744  NK9745  NK9746  NK9747 |  |  | NK9631 *P_EST2_ (-30 – -30)::HYG* | |
| NK9687  NK9688  NK9689  NK9690 | *MATa his3Δ1 leu2Δ0 met15Δ0 ura3Δ0 HTB2-GFP::HIS3 TUB1-pHIS3-mRuby2-TUB1::URA3* | | NK7300 Addgene pl. 50639*/BsaBI* | |
| NK9730  NK9731  NK9732  NK9733 | *MATa his3Δ1 leu2Δ0 met15Δ0 ura3Δ0 HTB2-GFP::HIS3 TUB1-pHIS3-mRuby2-TUB1::URA3 P_EST2_ (-30 – -30)::HYG* | | NK9687 *P_EST2_ (-30 – -30)::HYG* | |
| NK9737  NK9738  NK9739  NK9740 |  |  | NK9688 *P_EST2_ (-30 – -30)::HYG* | |
| NK9775  NK9776  NK9777 | *MATa his3Δ1 leu2Δ0 met15Δ0 ura3Δ0 HTB2-GFP-HIS3 bfa1::URA3 P_EST2_ Δ(-17 – -30)::HYG* | | NK9632 *P_EST2_ Δ(-17 – -30)::HYG* | |
| NK9782  NK9783  NK9784 |  |  | NK9633 *P_EST2_ Δ(-17 – -30)::HYG* | |
| NK9778  NK9779  NK9780  NK9781 | *MATa his3Δ1 leu2Δ0 met15Δ0 ura3Δ0 HTB2-GFP-HIS3 bfa1::URA3 P_EST2_ (-30 – -30)::HYG* | | NK9632 *P_EST2_ (-30 – -30)::HYG* | |
| NK9785  NK9786  NK9787  NK9788 |  |  | NK9633 *P_EST2_ (-30 – -30)::HYG* | |
| NK10092  NK10093  NK10094 | *MATa his3Δ1 leu2Δ0 met15Δ0 ura3Δ0 HTB2-GFP-HIS3 trp1::URA3* | | NK7300 *trp1::URA3* | |
| NK10095  NK10109  NK10166 | *MATa his3Δ1 leu2Δ0 met15Δ0 ura3Δ0 MYO1-GFP-HIS3 trp1::URA3* | | NK7301 *trp1::URA3* | |
| NK10096  NK10097  NK10098 | *MATa his3Δ1 leu2Δ0 met15Δ0 ura3Δ0 HTB2-GFP-HIS3 MYO1-mCherry-KAN* | | NK7300 *MYO1-mCherry::KAN* | |
| NK10099  NK10100  NK10101 | *MATa his3Δ1 leu2Δ0 met15Δ0 ura3Δ0 HTB2-GFP::HIS3 NOP56-mCherry::KAN* | | NK7300 *NOP56-mCherry::KAN* | |
| NK10117  NK10118 | *MATa his3Δ1 leu2Δ0 met15Δ0 ura3Δ0 HTB2-GFP-HIS3 MYO1-mCherry-KAN P_EST2_ (-30 – -30)::HYG* | | NK10096 *P_EST2_ (-30 – -30)::HYG* | |
| NK10119  NK10120 |  |  | NK10097 *P_EST2_ (-30 – -30)::HYG* | |
| NK10121  NK10122 | *MATa his3Δ1 leu2Δ0 met15Δ0 ura3Δ0 HTB2-GFP::HIS3 NOP56-mCherry::KAN P_EST2_ (-30 – -30)::HYG* | | NK10099 *P_EST2_ (-30 – -30)::HYG* | |
| NK10123  NK10124 |  |  | NK10100 *P_EST2_ (-30 – -30)::HYG* | |
| NK10278  NK10279  NK10280  NK10281 | *MATa his3Δ1 leu2Δ0 met15Δ0 ura3Δ0 HTB2-GFP-HIS3 PDS1-tdTomato-NAT* | | NK7300 *PDS1-tdTomato::NAT* | |
| NK10295  NK10296 | *MATa his3Δ1 leu2Δ0 met15Δ0 ura3Δ0 HTB2-GFP-HIS3 PDS1-tdTomato-NAT P_EST2_ (-30 – -30)::HYG* | | NK10278 *P_EST2_ (-30 – -30)::HYG* | |
| NK10297  NK10298  NK10299  NK10300 |  |  | NK10279 *P_EST2_ (-30 – -30)::HYG* | |
| NK10337  NK10338  NK10339 | *MATa his3Δ1 leu2Δ0 met15Δ0 ura3Δ0 HTB2-GFP-HIS3 pds1-m9-URA3* | | NK7300 pYT1223/ClaI, SacI | |
| NK10344  NK10345  NK10346 | *MATa his3Δ1 leu2Δ0 met15Δ0 ura3Δ0 HTB2-GFP-HIS3 PDS1_URA3* | | NK7300 pYT1223/MfeI, SacI | |
| NK10392  NK10393  NK10394  NK10395  NK10396 | *MATa his3Δ1 leu2Δ0 met15Δ0 ura3Δ0 HTB2-GFP-HIS3 pds1-m9-URA3 P_EST2_ (-30 – -30)::HYG* | | NK10338 *P_EST2_ (-30 – -30)::HYG* | |
| NK10397  NK10398  NK10399  NK10400  NK10401 |  |  | NK10339 *P_EST2_ (-30 – -30)::HYG* | |
| NK10408  NK10409  NK10410 | *MATa his3Δ1 leu2Δ0 met15Δ0 ura3Δ0 HTB2-GFP-HIS3 PDS1-URA3 P_EST2_ (-30 – -30)::HYG* | | NK10344 *P_EST2_ (-30 – -30)::HYG* | |
| NK10411  NK10412  NK10413 |  |  | NK10345 *P_EST2_ (-30 – -30)::HYG* | |
| NK10507  NK10508  NK10509  NK10510 | *MATa his3Δ1 leu2Δ0 met15Δ0 ura3Δ0 HTB2-GFP-HIS3 lte1::URA3* | | NK7300 *lte1::URA3* | |
| NK10525  NK10526  NK10527  NK10528  NK10529  NK10530 | *MATa his3Δ1 leu2Δ0 met15Δ0 ura3Δ0 HTB2-GFP-HIS3 lte1::URA3 P_EST2_ (-30 – -30)::HYG* | | NK10507 *P_EST2_ (-30 – -30)::HYG* | |
| NK10531  NK10532 |  |  | NK10508 *P_EST2_ (-30 – -30)::HYG* | |
| NK10770  NK10771 | *MATa his3Δ1 leu2Δ0 met15Δ0 ura3Δ0 HTB2-GFP-HIS3 trp1::URA3 PDS1-13MYC::TRP1* | | NK10092 *PDS1-13MYC::TRP1* | |
| NK10772 |  |  | NK10093 *PDS1-13MYC::TRP1* | |
| NK10780  NK10781  NK10782 | *MATa his3Δ1 leu2Δ0 met15Δ0 ura3Δ0 HTB2-GFP-HIS3 EST2-13MYC::KAN* | | NK7300 *EST2-13MYC::KAN* | |
| NK10792  NK10793 | *MATa his3Δ1 leu2Δ0 met15Δ0 ura3Δ0 HTB2-GFP-HIS3 trp1::URA3 PDS1-13MYC-TRP1 P_PDS1_ Δ(-90 – -200)::KAN* | | NK10770 *P_PDS1_ Δ(-90 – -200)::KAN* | |
| NK10794 |  |  | NK10771 *P_PDS1_ Δ(-90 – -200)::KAN* | |
| NK10806  NK10807 | *MATa his3Δ1 leu2Δ0 met15Δ0 ura3Δ0 HTB2-GFP-HIS3 EST2-13MYC-KAN P_EST2_ Δ(-17 – -30)::HYG* | | NK10781 *P_EST2_ Δ(-17 – -30)::HYG* | |
| NK10808 |  |  | NK10782 *P_EST2_ Δ(-17 – -30)::HYG* | |
| NK10809  NK10810 | *MATa his3Δ1 leu2Δ0 met15Δ0 ura3Δ0 HTB2-GFP::HIS3 EST2-13MYC-KAN P_EST2_ (-30 – -30)::HYG* | | NK10781 *P_EST2_ (-30 – -30)::HYG* | |
| NK10811 |  |  | NK10782 *P_EST2_ (-30 – -30)::HYG* | |
| NK10867 NK10868 | *MATa his3Δ1 leu2Δ0 met15Δ0 ura3Δ0 HTB2-GFP::HIS3 trp1::URA3 PDS1-13MYC-TRP1 P_EST2_ (-30 – -30)::HYG* | | NK10770 *P_EST2_ (-30 – -30)::HYG* | |
| NK10869  NK10870 |  |  | NK10771 *P_EST2_ (-30 – -30)::HYG* | |
| NK10871  NK10872 | *MATa his3Δ1 leu2Δ0 met15Δ0 ura3Δ0 HTB2-GFP::HIS3 trp1::URA3 PDS1-13MYC-TRP1 P_PDS1_ Δ(-90 – -200)::KAN P_EST2_ (-30 – -30)::HYG* | | NK10794 *P_EST2_ (-30 – -30)::HYG* | |
| NK10873  NK10874 |  |  | NK10792 *P_EST2_ (-30 – -30)::HYG* | |
| ***Saccharomyces cerevisiae* A364a** | | | | |
| NK1267 | *MATa-inc trp1-289 ura3::NAT leu2::LEU2-P_GAL1_-HO MNT2::HOsite-URA3-STAR-TEL* | | (Makovets and Blackburn, 2009) | |
| NK1325 | *MATa-inc trp1-289 ura3::NAT leu2::LEU2-*  *P_GAL1_-HO HEM13::HOsite-URA3* | | (Makovets and Blackburn, 2009) | |
| NK7960 | *MATa-inc trp1-289 ura3::NAT leu2::LEU2-P_GAL1_-HO MNT2::HOsite-URA3-STAR-TEL RAD52-mNeonGreen::TRP1* | | NK1267 *RAD52-mNeonGreen::TRP1* | |
| NK7961 |  |  |  |  |
| NK7962 |  |  |  |  |
| NK7963 | *MATa-inc trp1-289 ura3::NAT leu2::LEU2-P_GAL1_-HO HEM13::HOsite-URA3 RAD52-mNeonGreen::TRP1* | | NK1325 *RAD52-mNeonGreen::TRP1* | |
| NK7964 |  |  |  |  |
| NK7965 |  |  |  |  |
| NK7966 |  |  |  |  |
| NK7967 |  |  |  |  |
| NK7968 |  |  |  |  |
| NK9714 | *MATa bar1::LEU2 trp1-289 ura3-5 leu2-3,112 HTB2-mCherry::KAN* | | NK1 *HTB2-mCherry::KAN* | |
| NK9715 |  |  |  |  |
| NK9770 | *MATa-inc trp1-289 ura3::NAT leu2::LEU2-P_GAL1_-HO MNT2::HOsite-URA3-STAR-TEL RAD52-mNeonGreen::TRP1 HTB2-mCherry::KAN* | | NK7960 *HTB2-mCherry::KAN* | |
| NK9797 |  |  |  |  |
| NK9798 |  |  |  |  |
| NK9771 |  |  | NK7961 *HTB2-mCherry::KAN* | |
| NK9772 |  |  |  |  |
| NK9773 | *MATa-inc trp1-289 ura3::NAT leu2::LEU2-*  *P_GAL1_-HO HEM13::HOsite-URA3 RAD52-mNeonGreen::TRP1 HTB2-mCherry::KAN* | | NK7963 *HTB2-mCherry::KAN* | |
| NK9774 |  |  |  |  |
| NK9779 |  |  | NK7964 *HTB2-mCherry::KAN* | |
| NK9780 |  |  |  |  |
| NK9781 |  |  |  |  |
| NK11307 | *MATa/α CAN1/can1Δ::URA3*  *MET6/ met6Δ::TRP1 PDS1/PDS1* | | *MATa* *can1Δ::URA3 x MATα met6Δ::TRP1* | |
| NK11308 |  |  |  |  |
| NK11364 |  |  |  |  |
| NK11365 |  |  |  |  |
| NK11352 | *MATa/α CAN1/can1Δ::URA3*  *MET6/ met6Δ::TRP1 PDS1/pds1Δ::KAN* | | NK11307 *pds1Δ::KAN* | |
| NK11354 |  |  | NK11308 *pds1Δ::KAN* | |
| NK11384 |  |  | NK11364 *pds1Δ::KAN* | |
| NK11386 |  |  | NK11365 *pds1Δ::KAN* | |
| NK11343 | *MATΔ/α CAN1/can1Δ::URA3*  *MET6/ met6Δ::TRP1 PDS1/PDS1* | | NK11307 *MATa∆::LEU2* | |
| NK11344 |  |  | NK11308 *MATa∆::LEU2* | |
| NK11366 |  |  | NK11364 *MATa∆::LEU2* | |
| NK11368 |  |  | NK11365 *MATa∆::LEU2* | |
| NK11356 | *MATΔ/α CAN1/can1Δ::URA3*  *MET6/ met6Δ::TRP1 PDS1/pds1Δ::KAN* | | NK11343 *pds1Δ::KAN* | |
| NK11359 |  |  | NK11344 *pds1Δ::KAN* | |
| NK11388 |  |  | NK11366 *pds1Δ::KAN* | |
| NK11390 |  |  | NK11368 *pds1Δ::KAN* | |
| B. **Oligonucleotides** | | | |  |
| Primer number | | Sequence (5′ to 3′) | Purpose |  |
| OSM32 | | CACCCACACCCACACCCACACCCA | Telomere-specific probe |  |
| OSM88 | | AGAAACGCCATAGAAAAGAGCATAGTGAGAAAATCTTCAACATCAGGGCTcggatccccgggttaattaa | *RAD9* deletion |  |
| OSM89 | | AATTTCATCTAACCTCAGAAATAGTGTTGTATATATCATTGTCCGTAATAgaattcgagctcgtttaaac | *RAD9* deletion |  |
| OSM166 | | AAAGTAAAGCATGGCAATGAATGACACAAGTGAAATAGAAAAGTGAAAAAcggatccccgggttaattaa | *EST2* deletion |  |
| OSM167 | | GCAATATTTTCCTTATCAGCATCATAAGCTGTCAGTATTTCATGTATTATTAGTACgaattcgagctcgtttaaac | *EST2* deletion |  |
| OSM294 | | AAGTTTGACGTAAAGAAAAAAGTTCGAAAACTTTTGTAGCAGTTGAAAGTTTTGTATGCTcggatccccgggttaattaa | *BFA1* deletion |  |
| OSM295 | | AAGAGAAATGAATGTACTCAAGATAACGGTAAAGAAACAGTTATAAGAAGGCTAAAGGGCgaattcgagctcgtttaaac | *BFA1* deletion |  |
| OSM910 | | TATTGAGCACGTGAGTATACGTGATTAAGCACACAAAGGCAGCTTGGAGTcggatccccgggttaattaa | *TRP1* deletion |  |
| OSM911 | | TGCAGGCAAGTGCACAAACAATACTTAAATAAATACTACTCAGTAATAACgaattcgagctcgtttaaac | *TRP1* deletion |  |
| OSM2693 | | GAACTCGAATAAGATTTTCATGAGTATAAATCAGTTTTTTTTTTTTTTTTTcggatccccgggttaattaa | Cassette insertion into the *EST2* promoter |  |
| OSM3068 | | GGAATGAAAAAAAGTAAAGCATGGCAATGAATGACACAAGTGAAATAGAAAAGTGAAAAATTAAAAAAAAaattcgagctcgtttaaac | Cassette insertion into the *EST2* promoter |  |
| OSM3069 | | AAGCTTGTCTTGAATGAACTCGAATAAGATTTTCATGAGTATAAATCAGTTTTTTTcggatccccgggttaattaa | Cassette insertion into the *EST2* promoter |  |
| OSM3168 | | AAGCTTGTCTTGAATGAACTCGAATAAGATTTTCATGAGTATAAATCAGTTTTcggatccccgggttaattaa | Cassette insertion into the *EST2* promoter |  |
| OSM3169 | | TTGTCTTGAATGAACTCGAATAAGATTTTCATGAGTATAAATCAGTTTTTTTTTTTcggatccccgggttaattaa | Cassette insertion into the *EST2* promoter |  |
| OSM3170 | | TTGTCTTGAATGAACTCGAATAAGATTTTCATGAGTATAAATCAGTTTTTTTTTTTTTTTcggatccccgggttaattaa | Cassette insertion into the *EST2* promoter |  |
| OSM3636 | | CTGAAAATAATATGTCAGAAACATTCGCAACTCCCACTCCCAATAATCGAcggatccccgggttaattaa | Tagging *SPC42* at the C-terminus |  |
| OSM3637 | | CACAGAACGCTTTAAGAATGCGCCATACTCCTTAACTGCTTTTTAAATCAgaattcgagctcgtttaaac | Tagging *SPC42* at the C-terminus |  |
| OSM3638 | | GTTAAGGTAAATCCGTCTGATGACG | To screen after tagging *SPC42* at the C-terminus |  |
| OSM3706 | | AAAAGAAAGATAAAAAGGAAAAGAAGGATAAAAAGAAGAAAAGTAAGGATcggatccccgggttaatt | Tagging *NOP56* at the C-terminus |  |
| OSM3707 | | AAAAGTACACAAAAAGATGGGATATACTTTATTTCGATTCATTGTTCCTTgaattcgagctcgtttaaac | Tagging *NOP56* at the C-terminus |  |
| OSM3708 | | ATAAGAGGGTACTTAAGCTCAAAATAAC | To screen after tagging *NOP56* at the C-terminus |  |
| OSM4103 | | TCTCCGAAGGTACTAGGGCTGTTACCAAATACTCCTCCTCTACTCAAGCCcggatccccgggttaattaa | Tagging *HTB2* at the C-terminus |  |
| OSM4104 | | GCCACTAATAAAAAGAAAACATGACTAAATCACAATACCTAGTGAGTGACgattcgagctcgtttaaac | Tagging *HTB2* at the C-terminus |  |
| OSM4313 | | TAGGCTCGAAAAATATTGATAGTAACAATGCACAGAGTAAAATTTTCAGTcggatccccgggttaattaa | Tagging *MYO1* at the C-terminus |  |
| OSM4314 | | GTTAATAATGCATATTCTCATTCTGTATATACAAAACATCTCATCATTATgaattcgagctcgtttaaac | Tagging *MYO1* at the C-terminus |  |
| OSM4315 | | AGGAAGCTACTTTGAAATATGAGGC | To screen after tagging *MYO1* at the C-terminus |  |
| OSM4319 | | TGTACAGCGAAGAAGGCCTCGATCCTGAAGAACTAGAGGACTTAGTTACTcggatccccgggttaattaa | Tagging *PDS1* at the C-terminus |  |
| OSM4320 | | ATCTGTATATACGTGTATATATGTTGTGTGTATGTGAATGAGCAGTGGATgaattcgagctcgtttaaac | Tagging *PDS1* at the C-terminus |  |
| OSM4321 | | CTTGGAAGACGAAGATGACACC | To screen after tagging *PDS1* at the C-terminus |  |
| OSM4369 | | TTGAACTCCACCAGGTAGTG | To screen C-terminal tagging by tdTomato |  |
| OSM4370 | | CTTTGATGACGGCCATGTTG | To screen C-terminal tagging by tdTomato |  |
| OSM4371 | | GCTTCAGCCTCTGCTTGATC | To screen C-terminal tagging by mCherry |  |
| OSM4397 | | ccccACTAGTTTTAAAAGATCTTGCAGATTCAGGTAAAAAT | To remove the 3HA sequence from the pHW310 (Wang *et al.*, 2001) |  |
| OSM4398 | | GCTCATTAAATAGTCCTAGGCCACC | To remove the 3HA sequence from the pHW310 (Wang *et al.*, 2001) |  |
| OSM4424 | | TGTTTGCTTTACATAGCCTCTAGCCATTCAAAGATTCGCTACCCTGAACCcggatccccgggttaattaa | *LTE1* deletion |  |
| OSM4425 | | AAGTTTTTTACAAGAGGAATTTGGGAACTGGAGGAAAGTGGCACAATACCgaattcgagctcgtttaaac | *LTE1* deletion |  |
| OSM4445 | | TCTCCGAAGGTACTAGGGCTGTTACCAAATACTCCTCCTCTACTCAAGCCggtgacggtgctggttta | Tagging *HTB2* at the C-terminus |  |
| OSM4446 | | GCCACTAATAAAAAGAAAACATGACTAAATCACAATACCTAGTGAGTGACtcgatgaattcgagctcg | Tagging *HTB2* at the C-terminus |  |
| OSM4529 | | GTTCTCTTTCTATTGGGCACACATATAATACCCAGCAAGTCAGCATCGGAcggatccccgggttaattaa | *TRP1* deletion |  |
| OSM4530 | | GAAGCATTTAATAGAACAGCATCGTAATATATGTGTACTTTGCAGTTATGgaattcgagctcgtttaaac | *TRP1* deletion |  |
| OSM4577 | | CACTTTCCGTGCGACGTGTGTATTTGTTAGCGTAGGTTGCTTATCGTTTTgaattcgagctcgtttaaac | Cassette insertion into the *PDS1* promoter |  |
| OSM4578 | | CTTTTTGACCTCTTACACGAGTTTTTTGACGCGTTTGAGATCTTTGTGTGcggatccccgggttaattaa | Cassette insertion into the *PDS1* promoter |  |
| OSM4579 | | GATTTAAAGCTGCATTTATTTTGTTTGTTTATTTTGTTTGTTTTTCTTTTTcggatccccgggttaattaa | Cassette insertion into the *PDS1* promoter |  |
| OSM4580 | | CTTCGCCGAATATTGATTTAAAGCTGCATTTATTTTGTTTGTTTATTTTGcggatccccgggttaattaa | Cassette insertion into the *PDS1* promoter |  |
| OSM4581 | | AGAAGTGTAATAGTATGTGAATGGGAAAAACCGCTAGCAACCGTTACTTGcggatccccgggttaattaa | Cassette insertion into the *PDS1* promoter |  |
| OSM4582 | | AAAGGTTTATTATCTAGCACTTAATCTAGCTTGGTACCGCAGAAGTGTAAcggatccccgggttaattaa | Cassette insertion into the *PDS1* promoter |  |
| OSM4591 | | CTAAGTAACTAAGTCCTCTAGTTCTTCAGGATCGAGGCCTTCTTCGCTGTcggatccccgggttaattaa | *PDS1* deletion |  |
| OSM4707 | | TCGTCAATCGAAAGTTTATTTCAGAGTTCTTCAGACTTCTTAACTCCTGTttgaaatttttttgattcgg | *can1::URA3* |  |
| OSM4708 | | AGAATGCGAAATGGCGTGGAAATGTGATCAAAGGTAATAAAACGTCATATggtaataactgatataatt | *can1::URA3* |  |
| OSM4718 | | ATGTTCTTTGCAAACCAACTTTCTAGTATTCGGACATTTTCTTTTGTAAAGGATAATTATACTCTATTTCTC | *MATa::LEU2* |  |
| OSM4719 | | AACCCGACTATGCTATTTTAATCATTGAAAACGAATTTATTTAGATCTCACGTTGAGCCATTAGTATCAATTTG | *MATa::LEU2* |  |
| OSM4722 | | GTACATATCAAAAATGGTTCAATCTGCTGTCTTAGGGTTCCCAAGAATCGGggatccccgggttaattaa | *MET6* deletion |  |
| OSM4723 | | ACCTTAATTCTTGTATTGTTCACGGAAGTACTTGGCGGCTTCGACCATATgaattcgagctcgtttaaac | *MET6* deletion |  |
| C. **Plasmids** | | | |  |
| Plasmid | | Description | Origin/References |  |
| pFA6- kanMX6 | | To amplify a cassette for gene deletion with *KAN* marker | (Longtine *et al.*, 1998) |  |
| pFA6a-natMX6 | | To amplify a cassette for gene deletion with *NAT* marker | (Sato et al., 2005) |  |
| pFA6a-hphMX6 | | To amplify a cassette for gene deletion with *HYG* marker | (Sato et al., 2005) |  |
| pFA6a-mCherry-kanMX6 | | To amplify a cassette for C-terminal tagging with mCherry | Received from Prof Kenneth Sawin lab |  |
| pFA6a-13myc-kanMX6 | | To amplify a cassette for C-terminal tagging with Myc | (Longtine *et al.*, 1998) |  |
| Addgene pl. 50639 | | *pHIS3p:mRuby2-TUB1+3'UTR::URA3*.  To integrate a second copy of mRuby2-Tub1 | (Markus *et al.*, 2015) |  |
| AMp751 | | *pFA6a-tdTomato-NATMX6.* To amplify a cassette for C-terminal tagging with tdTomato | Received from Prof Adele Marston lab |  |
| pYT811 | | *pFA6a-URA3*.  To amplify a cassette for gene deletion with *URA3* marker | Makovets lab plasmid collection |  |
| pHW310 | | pUC19-pds1-HA-m9::URA3.  To replace endogenous *PDS1* with the mutated version *pds1-HA-m9* | (Wang *et al.*, 2001)  Received from Prof Stephen Elledge lab |  |
| pYT1223 | | *pUC19-pds1-m9::URA3.*  To replace endogenous *PDS1* with the mutated version *pds1-m9* | The PCR product (OSM4397 + OSM4398 on NK7300 genomic DNA) was digested with SpeI and AvrII and ligated into pHW310 digested with SpeI and AvrII. The result was confirmed by sequencing. |  |

**Supplemental References**

Huh, W.K., J. Falvo, L. Gerke, A.S. Carroll, R.W. Howson, J.S. Weissman and E.K. O'Shea. 2003. Global analysis of protein localization in budding yeast. *Nature.* 425, 686–691.

# Longtine, M.S., A. McKenzie 3rd, D.J. Demarini, N.G. Shah, A. Wach, A. Brachat, P. Philippsen, J.R. Pringle. 1998. Additional modules for versatile and economical PCR-based gene deletion and modification in Saccharomyces cerevisiae. Yeast. 14:953-961.

Makovets, S., and E.H. Blackburn. 2009. DNA damage signalling prevents deleterious telomere addition at DNA breaks. *Nat Cell Biol*. 11:1383-1386.

Markus, S.M., S. Omer, K. Baranowski, and W.L. Lee. 2015. **Improved Plasmids for Fluorescent Protein Tagging of Microtubules in *Saccharomyces cerevisiae***. *Traffic.* 16:773-786.

# Sato, M., S. Dhut, and T. Toda 2005. New drug-resistant cassettes for gene disruption and epitope tagging in *Schizosaccharomyces pombe*. *Yeast.* 22(7):583-591.

Wang, H., D. Liu, Y. Wang, J. Qin, and S.J. Elledge. 2001. Pds1 phosphorylation in response to DNA damage is essential for its DNA damage checkpoint function. *Genes Dev*. 15:1361-1372.
